# Genetic patterning for child psychopathology is distinct from that for adults and implicates fetal cerebellar development

**DOI:** 10.1038/s41593-023-01321-8

**Published:** 2023-05-18

**Authors:** Dylan E. Hughes, Keiko Kunitoki, Safia Elyounssi, Mannan Luo, Oren M. Bazer, Casey E. Hopkinson, Kevin F. Dowling, Alysa E. Doyle, Erin C. Dunn, Hamdi Eryilmaz, Jodi M. Gilman, Daphne J. Holt, Eve M. Valera, Jordan W. Smoller, Charlotte A. M. Cecil, Henning Tiemeier, Phil H. Lee, Joshua L. Roffman

**Affiliations:** 1 Department of Psychiatry, Massachusetts General Hospital, Boston, MA, USA; 2 Department of Psychiatry, Massachusetts General Hospital and Harvard Medical School, Boston, MA, USA; 3 Department of Psychology, Education and Child Studies, Erasmus University Rotterdam, Rotterdam, the Netherlands; 4 Generation R Study Group, Erasmus MC, University Medical Center Rotterdam, Rotterdam, the Netherlands; 5 Translational Neuroscience Program, Department of Psychiatry, University of Pittsburgh School of Medicine, Pittsburgh, PA, USA; 6 Medical Scientist Training Program, University of Pittsburgh and Carnegie Mellon University, Pittsburgh, PA, USA; 7 Center for Genomic Medicine, Massachusetts General Hospital, Boston, MA, USA; 8 Psychiatric and Neurodevelopmental Genetics Unit, Center for Genomic Medicine, Massachusetts General Hospital, Boston, MA, USA; 9 Stanley Center for Psychiatric Research, The Broad Institute of Harvard and MIT, Cambridge, MA, USA; 10 Center on the Developing Child at Harvard University, Cambridge, MA, USA; 11 MGH/HST Athinoula A. Martinos Center for Biomedical Imaging, Department of Radiology, Massachusetts General Hospital, Charlestown, MA, USA; 12 Center for Precision Psychiatry, Massachusetts General Hospital, Boston, MA, USA; 13 Department of Child and Adolescent Psychiatry/Psychology, Erasmus MC-Sophia, Rotterdam, the Netherlands; 14 Department of Epidemiology, Erasmus MC, Rotterdam, the Netherlands; 15 Molecular Epidemiology, Department of Biomedical Data Sciences, Leiden University Medical Center, Leiden, the Netherlands; 16 Department of Social and Behavioral Sciences, Harvard T.H. Chan School of Public Health, Boston, MA, USA

## Abstract

Childhood psychiatric symptoms are often diffuse but can coalesce into discrete mental illnesses during late adolescence. We leveraged polygenic scores (PGSs) to parse genomic risk for childhood symptoms and to uncover related neurodevelopmental mechanisms with transcriptomic and neuroimaging data. In independent samples (Adolescent Brain Cognitive Development, Generation R) a narrow cross-disorder neurodevelopmental PGS, reflecting risk for attention deficit hyperactivity disorder, autism, depression and Tourette syndrome, predicted psychiatric symptoms through early adolescence with greater sensitivity than broad cross-disorder PGSs reflecting shared risk across eight psychiatric disorders, the disorder-specific PGS individually or two other narrow cross-disorder (Compulsive, Mood-Psychotic) scores. Neurodevelopmental PGS-associated genes were preferentially expressed in the cerebellum, where their expression peaked prenatally. Further, lower gray matter volumes in cerebellum and functionally coupled cortical regions associated with psychiatric symptoms in mid-childhood. These findings demonstrate that the genetic underpinnings of pediatric psychiatric symptoms differ from those of adult illness, and implicate fetal cerebellar developmental processes that endure through childhood.

Risk for psychiatric disorders arises early in life, reflecting in part the cumulative effects of thousands of common genetic variants^1,2^. Data from ongoing genome-wide association studies (GWASs) provide updated templates to calculate individual risk for psychiatric disorders such as schizophrenia (SCZ), bipolar disorder (BIP) and autism spectrum disorder (ASD) through PGSs^3–5^. Along with gene expression data, PGS data have also provided new insights into the biological origins of psychiatric illness, supporting an essential role for synaptic organization^6,7^. Studies of PGS may ultimately lead to the development of clinically useful biomarkers that predict the occurrence of psychiatric illness, including in children who have yet to develop full-fledged illness and who may benefit from early intervention^8,9^.

Despite great promise, several factors currently limit the potential clinical application of PGSs in children. First, the studies used to derive PGSs, such as those conducted by the Psychiatric Genomics Consortium (PGC) and other large-scale efforts, have largely–and in many cases exclusively–enrolled adult participants^10^, even when disorders usually diagnosed in childhood, including ASD^4^ and attention deficit hyperactivity disorder (ADHD)^11,12^, are examined. However, the clinical relevance of genetic loading in children for disorders that usually present in adulthood remains uncertain. Moreover, even for disorders usually first diagnosed in children, these diagnoses may not persist or may change in adulthood^13^, potentially complicating the application of PGSs across development.

Second, even more so than in adults^14^, psychiatric symptoms in children tend to be poorly differentiated and often do not conform with discrete diagnostic categories^15,16^. Because established disease-specific PGSs were derived from case–control studies, it remains largely unclear whether these PGSs capture more subtle psychopathology in children. In research studies, such psychopathology is often assessed using dimensional measures that are continuous across healthy and disease populations, and are not bound by conventional, threshold-based diagnostic categories. This approach enables the identification of clinical features that may associate with genetic loading differently in children than in adults. For example, an emerging pattern for ADHD PGS suggests that increased PGS values in children are linked to a range of externalizing symptoms beyond inattention and hyperactivity, including aggression^17^. In contrast, studies relating SCZ PGS to psychotic symptoms in children have been inconsistent^18,19^, perhaps reflecting important differences in how psychosis is measured or experienced in children versus adults.

Third, given such clinical heterogeneity, the substantial overlap in PGSs across different psychiatric conditions^20–22^ further complicates the search for parsimonious relationships between polygenic risk indices and clinical syndromes in children. Cross-disorder PGSs account for genomic risk that overlaps across psychiatric conditions^20,22^, but again may not capture fluid relationships between clusters of genetic risk and emerging psychopathology in children. Further, uncertainty persists about when during neurodevelopment, and where within the brain, polygenic loading lays the foundation for psychiatric risk.

We leveraged genomic data and measures of psychopathology from the population-based Adolescent Brain Cognitive Development (ABCD) Study, and also from the Generation R Study as a replication cohort, to evaluate the relationships of disease-specific and cross-disorder PGSs to dimensional psychopathology in mid-childhood. In each cohort, we found that a latent neurodevelopmental factor (termed NDV) PGS, identified from the latest PGC cross-disorders study^21^, captured variance in dimensional psychopathology across numerous domains with greater sensitivity than any disease-specific or other cross-disorder PGS. Among eight neuropsychiatric disorders examined in the PGC cross-disorder study, the NDV factor represented genetic risk primarily shared among early NDV disorders, such as ASD, ADHD and Tourette syndrome, along with major depression^21^. Using data from postmortem gene expression atlases, we also found that NDV effects converged on synaptic organization within the fetal cerebellum, a pattern echoed by an association between cerebellar volumes derived from magnetic resonance imaging (MRI) and psychopathology within the ABCD sample.

## Results

### Participants

Data from two developmental cohort studies, ABCD and Generation R, were included in the clinical and genomic analyses; in addition, structural MRI data were analyzed from ABCD. The ABCD Study enrolled 11,875 children, aged 9-10, across 22 US sites. For the current analysis, we used complete data from baseline assessments (ages 9-10; *N* = 11,852; 47.8% female; 24.6% self-reported nonwhite) as well as all clinical follow-up data from year 2 (ages 11–12; *N* = 8,076; 47.7% female; 21.5% self-reported nonwhite) that were available in June 2022 (ABCD Data Release 4.0). The Generation R Study is a European prospective birth cohort study which follows the offspring of 9,778 mothers from fetal life to adulthood; we used all data from children at ages 9 (*N* = 1,850; 50.4% female) and 13 (*N* = 1,791; 50.3% female) that were available in June 2022.

### Dimensional psychopathology and PGSs

**Fig. 1:**
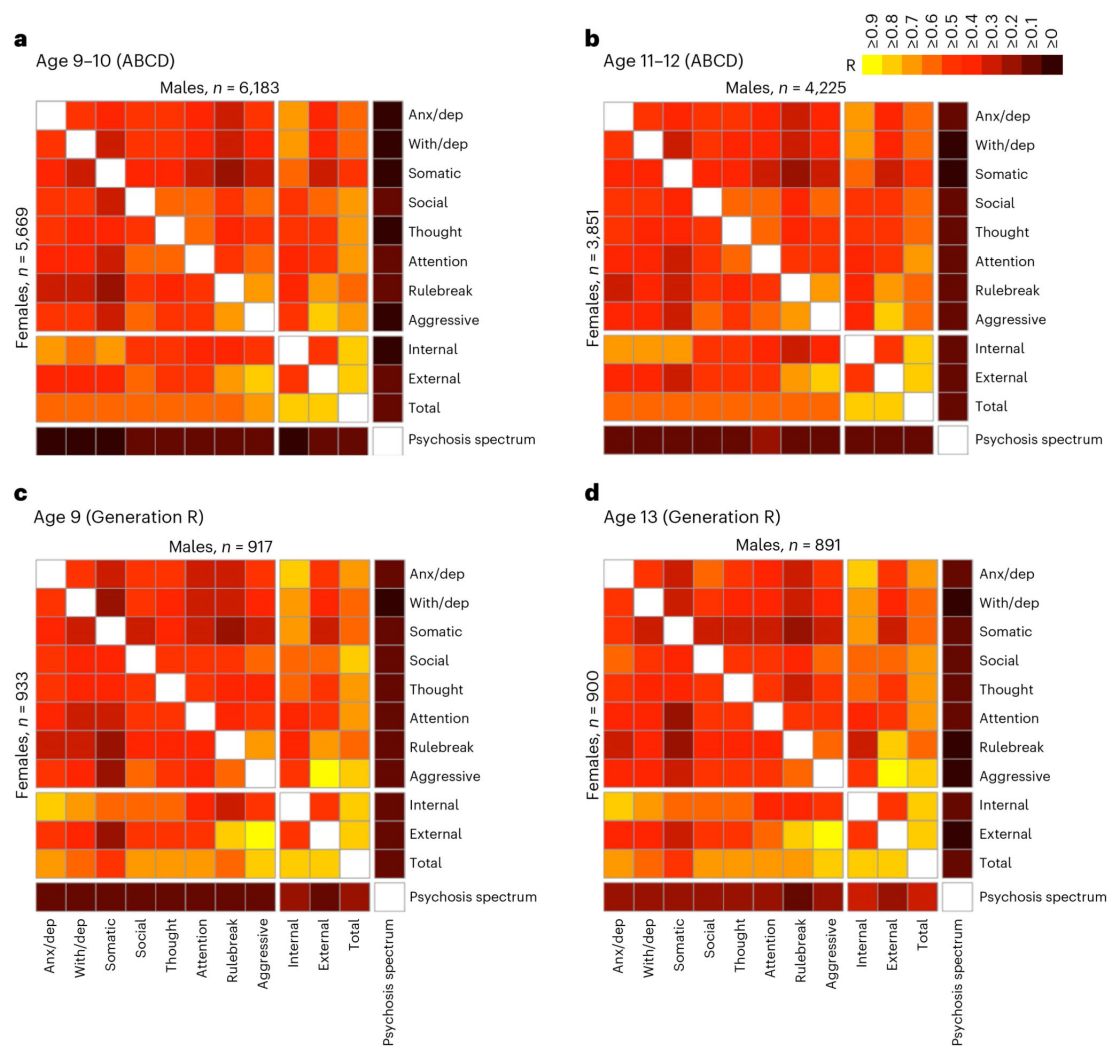
Pearson correlations among dimensional psychopathology measures in ABCD and Generation R cohorts (11 CBCL scales, and PQ-BC distress scores), stratified by sex. **a**,**b**, Correlation matrix of psychopathology in ABCD at ages 9-10 (*n* = 11,852) (**a**) and 11-12 (*n* = 8,076) (**b**). **c**,**d**, Correlation matrix of psychopathology in Generation R at ages 9 (*n* = 1,850) (**c**) and 13 (*n* = 1,791) (**d**). Males are represented in the top right of the matrices, and females in the bottom left. Colors represent strength of correlation coefficients (Pearson *r*) between respective variables (see legend). All correlations are statistically significant after correction for multiple comparisons using the FDR (*Q* < 0.05). Anx/dep, anxious/depressed; With/dep, withdrawn/depressed.

Dimensions of psychopathology in ABCD were measured using the Child Behavior Checklist (CBCL) and Prodromal Questionnaire–Brief Child Version (PQ-BC). CBCL items are organized into eight individual syndrome scales (anxious/depressed, withdrawn/depressed, somatic complaints, social problems, thought problems, attention problems, rule-breaking behavior and aggressive behavior) as well as three broader scales (Internalizing, Externalizing and Total symptoms). Consistent with earlier reports^16^, dimensions of psychopathology across the individual syndrome and broader scales demonstrated moderate-to-strong bivariate correlations both at baseline (*N* = 11,852; Pearson *r* values, 0.29–0.85; *P* values < 9.62 × 10^-227^) and at year 2 (*N* = 8,076; Pearson *r* values, 0.30–0.84; *P* values < 5.63 × 10^-167^; Fig. 1). Measurements within individuals tended to be stable over time (CBCL Total symptoms, *r* = 0.71; other CBCL scales, *r* values 0.53–0.69). In contrast, prodromal psychosis symptoms correlated comparatively weakly with CBCL domains at baseline (Pearson *r* values, 0.07–0.14; *P* values < 5.26 × 10^-14^) and year 2 (Pearson *r* values, 0.11–0.19; *P* values < 1.29 × 10^-22^), and were less consistent over time (*r* = 0.33). To replicate our findings, we leveraged data from the Generation R Study, a prospective birth cohort study which follows the offspring of 9,778 mothers from fetal life to adulthood. In this sample, the structure of psychopathology at age 9 (*N* = 1,850) and age 13 (*N* = 1,791) was comparable to that of the ABCD sample. Correlations among CBCL syndrome and broader scales were moderate to strong, underscoring the lack of differentiation of child psychopathology into discrete subtypes (age 9: Pearson *r* values, 0.25–0.98; *P* values < 1.32 × 10^-28^; age 13: Pearson *r* values, 0.26–0.97; *P* values < 4.07 × 10^-29^; Fig. 1). Although psychosis spectrum symptoms were measured using a different scale in Generation R (ref. 23) than in ABCD, they similarly showed a relatively weak correlation with CBCL scores (age 9: Pearson *r* values, 0.11–0.19; *P* values < 1.02 × 10^-06^; age 13: Pearson *r* values, 0.11–0.24; *P* values < 9.64 × 10^-06^).

**Extended Data Fig. 1:**
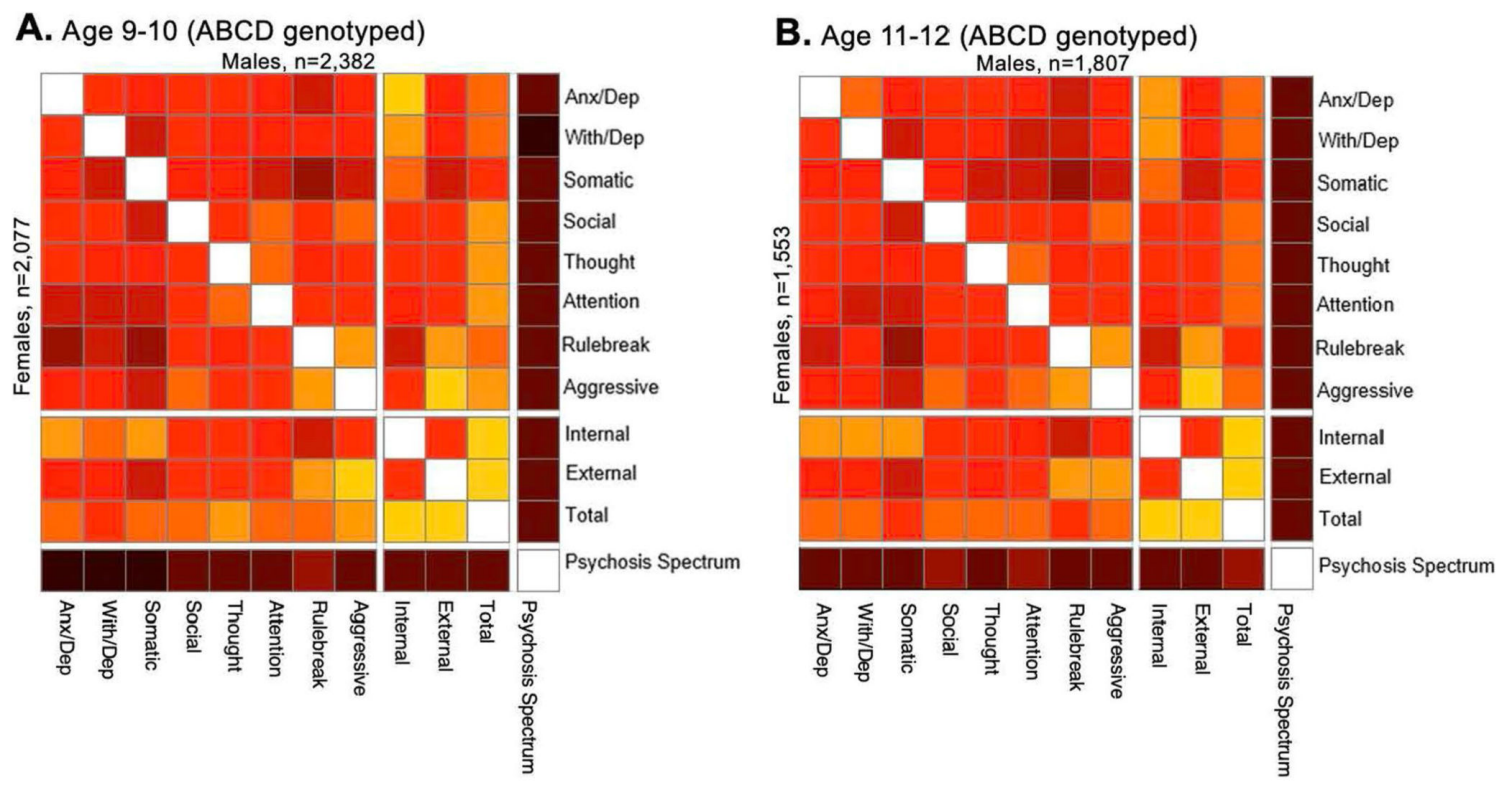
Pearson correlations among dimensional psychopathology measures in ABCD genotyped subjects only. (**a, b**) correlation matrix of psychopathology in genotyped males (top right of matrices) and females (bottom left of matrices) at ages 9-10 (*n* = 4,459; **A**) and 11-12 (*n* = 3,360; **B**).

**Fig. 2:**
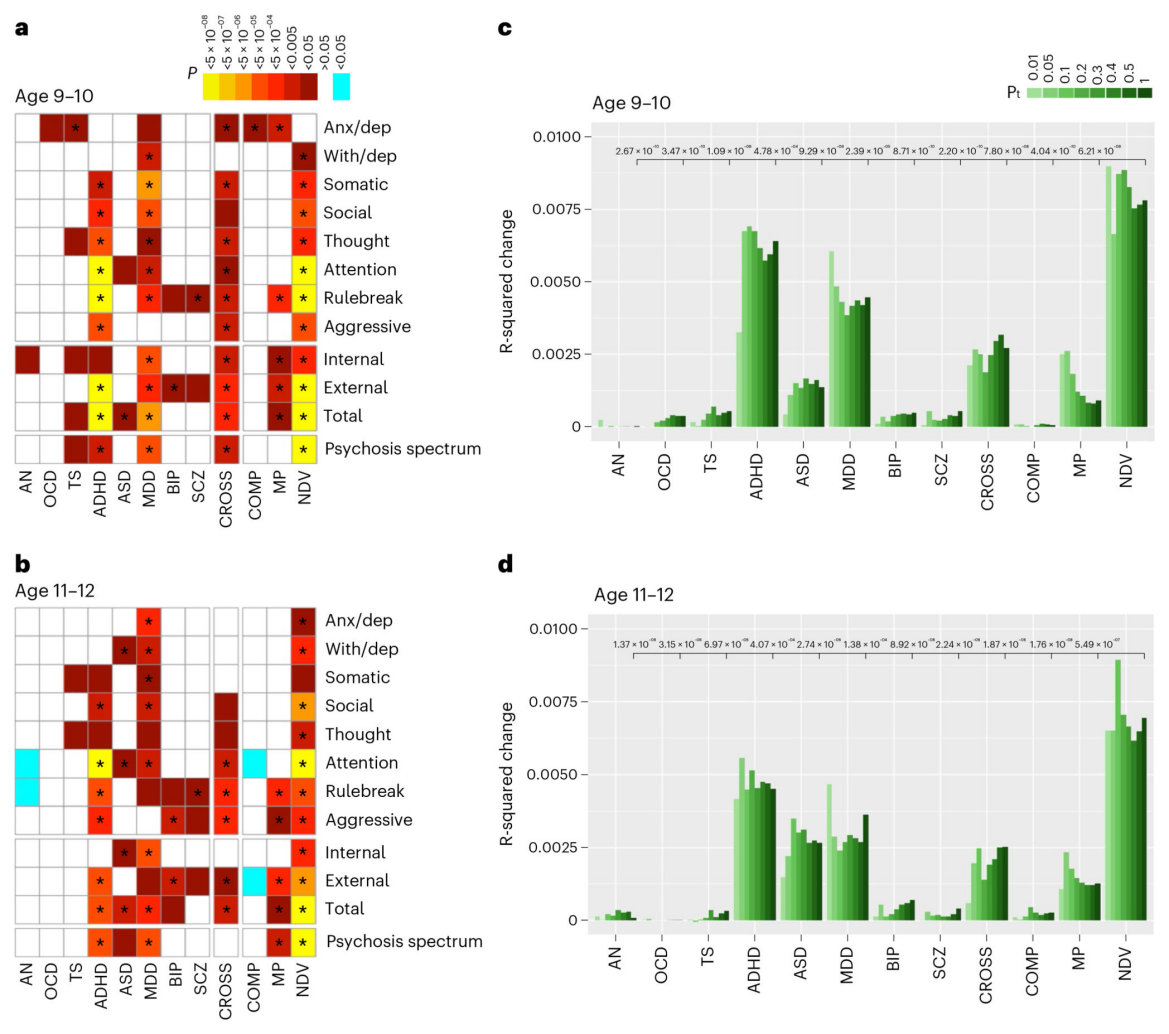
Prediction of dimensional psychopathology in unrelated young adolescents of European ancestry by disorder-specific and gSEM-derived PGSs. **a**, Prediction of dimensional psychopathology by PGSs of eight syndrome-specific and three broadband CBCL scores, and PQ-BC scores at age 9–10 (*n* = 4,459), with warm (red/yellow) colors indicating positive relationships and cool (blue) colors indicating negative relationships with PGS. White boxes indicate nonsignificant relationships (*P* > 0.05). Statistical significance and effect (coefficient) estimates are derived from linear mixed models regressing psychopathology on PGS covarying for age, sex and the top five genetic ancestry principal components as fixed effects and study site as a random effect. *P* values shown are uncorrected. Stars indicate tests that were significant after correcting for multiple comparisons using the FDR, *Q* < 0.05. **b**, Repeated analyses in the same participants at 11-12 (*n* = 3,360). **c**, Variance in total dimensional psychopathology (CBCL Total) explained by disorder-specific, cross-disorder and gSEM-derived PGSs in the ABCD sample at ages 9–10 (*n* = 4,459), with color shades reflecting SNP inclusion thresholds (Pt). Uncorrected *P* values (shown within the figure in black text near the *y*-max) represent the significance of the *R*^2^ change after adding NDV PGSs to base models that included each other PGS and nuisance covariates (see above), at the broadest SNP inclusion threshold (Pt = 1). **d**, Repeated analyses in the same participants at ages 11-12 (*n* = 3,360). All *P* values were two-sided. AN, anorexia nervosa; TS, Tourette syndrome.

Genotype data from 4,462 unrelated ABCD youths of European ancestry were used to generate individual participants’ PGSs for eight psychiatric illnesses (anorexia nervosa (AN), obsessive-compulsive disorder (OCD), Tourette syndrome (TS), ADHD, ASD, major depressive disorder (MDD), BIP, SCZ), plus a broad index of cross-disorder risk (CROSS) across the aforementioned eight disorders, using summary statistics from the PGC^2–4,11,21,24–27^. The symptom correlation matrix of genotyped participants closely resembled that of the entire sample (Extended Data Fig. 1). Consistent with previous reports using ABCD baseline data^28^ and other PGS studies of psychopathology within comparable age groups^28–30^, among all disorder-specific PGSs, ADHD and MDD most strongly predicted dimensional psychopathology scores at ages 9–10. Additionally, CROSS significantly predicted a broad range of symptom categories, including psychotic spectrum symptoms (Fig. 2a). This overall pattern was largely unchanged at the year 2 follow-up (ages 11–12; Fig. 2b).

Next, we used a recently reported method^31^ to detect latent clustering of cross-disorder genomic data through genomic structural equation modeling (gSEM). As per Lee et al.^21^, gSEM of the aforementioned eight-psychiatric-disorder GWAS identified three factors: NDV, which reflected loading of ADHD, ASD, MDD and Tourette syndrome PGSs; Compulsive (COMP), which reflected loading of anorexia nervosa, OCD and Tourette syndrome PGSs; and Mood-Psychotic (MP), which reflected loading of BIP, MDD and SCZ PGSs (Supplementary Table 1). Of these three gSEM-derived scores, NDV scores predicted the widest range of psychopathology (Fig. 2a,b and Supplementary Tables 2 and 3). COMP and MP PGSs contributed minimally to variance in CBCL Total at ages 9–10, and even less so at ages 11–12. Further, direct comparison of NDV versus all other individual PGSs, including disease-specific indices, indicated that NDV PGS accounted for significantly more variance in CBCL broader scales and psychosis spectrum symptoms at both timepoints, with the exception of MDD as a predictor of internalizing symptoms (Fig. 2c,d, Supplementary Fig. 1 and Supplementary Table 4). In a sensitivity analysis, we repeated baseline analyses using a different method for calculating PGS, PRS-CS, a Bayesian approach to PGS generation^32^, and found similar results (Supplementary Table 5).

**Extended Data Fig. 2:**
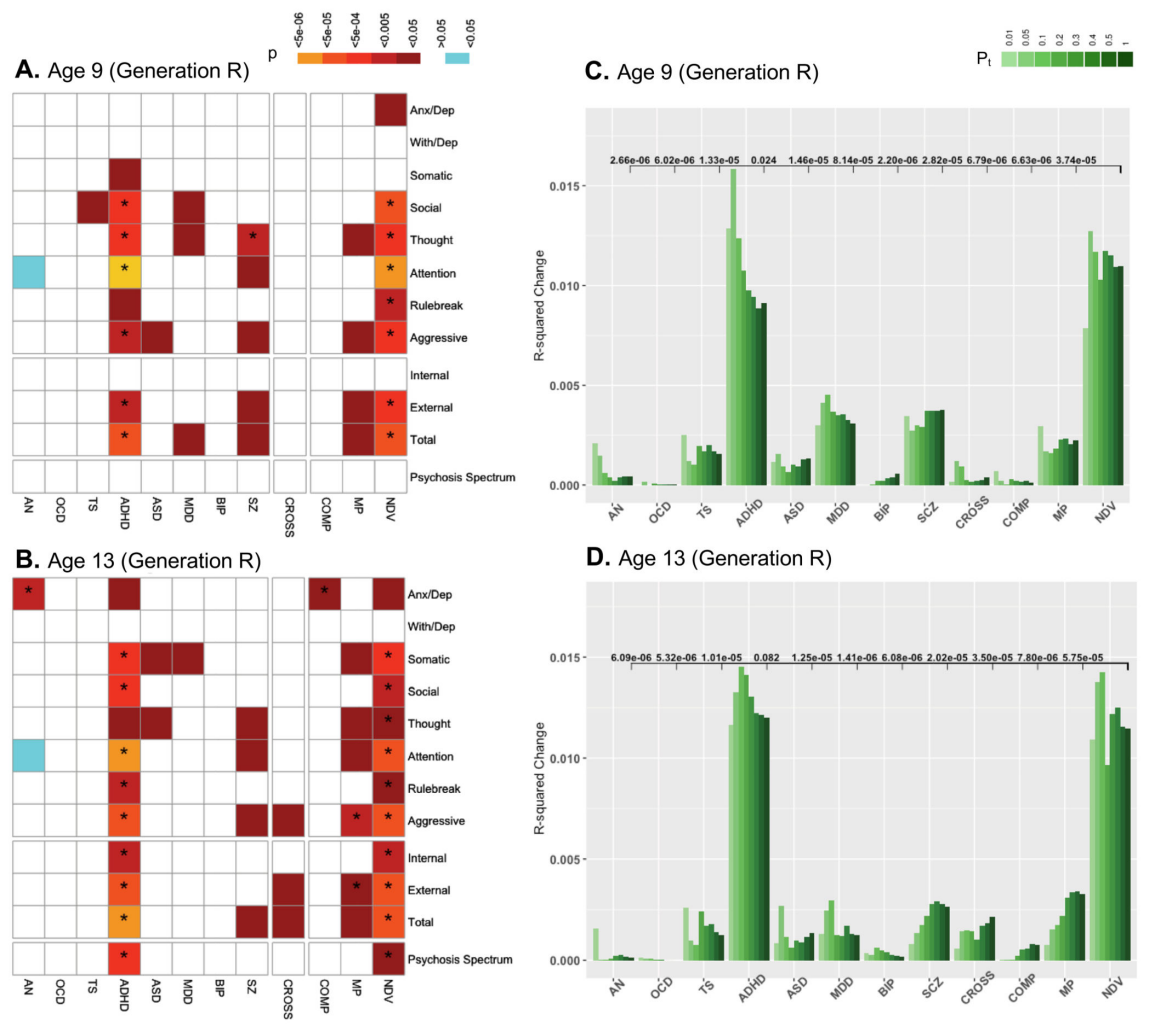
Relationship between gSEM-derived PGS and psychopathology in the Generation R cohort. (**a, b**) Heatmaps showing uncorrected p-values from linear regression models regressing psychopathology on PGS covarying for age, sex, and top 5 principal components at age 9 (*n* = 1,850; **A**) and 13 (*n* = 1,791; **B**). Asterisks indicate p<0.05 after False Discovery Rate correction for 36 comparisons (3 PGS x 12 measures of psychopathology). (**c, d**) Variance in CBCL Total accounted for by each gSEM-derived PGS. Uncorrected p-values (shown within the figure in black text near the y-max) represent the significance of the R^2^ change after adding NDV scores to base linear regression models including the respective PGS while covarying for age, sex, and top 5 principal components (Pt=1). All regressions represented are two-sided.

Independent analyses with both disorder-specific and gSEM-derived factors of CBCL Total symptoms in Generation R showed similar results. At both timepoints (ages 9 and 13), NDV PGS was associated with the widest spectrum of psychopathology, although differences between NDV and ADHD narrowed at age 13 (Extended Data Fig. 2a,b and Supplementary Tables 6 and 7). NDV PGS again had greater predictive power than all other PGSs for CBCL Total and Externalizing symptoms at age 9, although no PGSs predicted Internalizing or Psychosis Spectrum symptoms within the smaller Generation R cohort at that age. At age 13, NDV PGS outperformed all PGSs except for ADHD in predicting CBCL Total, Externalizing, Internalizing and Psychosis Spectrum symptoms (Extended Data Fig. 2c,d, Supplementary Fig. 2 and Supplementary Table 8).

The substantial overlap in scores among CBCL syndrome-specific scales reflects in part a shared general factor of psychopathology (‘p’), which has been parsed from residual (orthogonal) variance in more specific measures using bifactor models of baseline ABCD CBCL data^33^. Applying PGSs, we determined the extent to which genetic mapping onto multiple specific symptoms reflected associations of PGSs with ‘p’ in ABCD. Among PGSs, only NDV, ADHD and MDD genetic loading associated significantly with ‘p’, although NDV effects were significantly stronger than the others (*P* values ≤ 0.012). However, none of the ‘p’-residualized factors derived from either three-factor (‘p’, internalizing, externalizing) or nine-factor (‘p’ and the eight syndrome-specific factors) models significantly associated with NDV or any other PGS (Supplementary Table 9).

**Extended Data Fig. 3:**
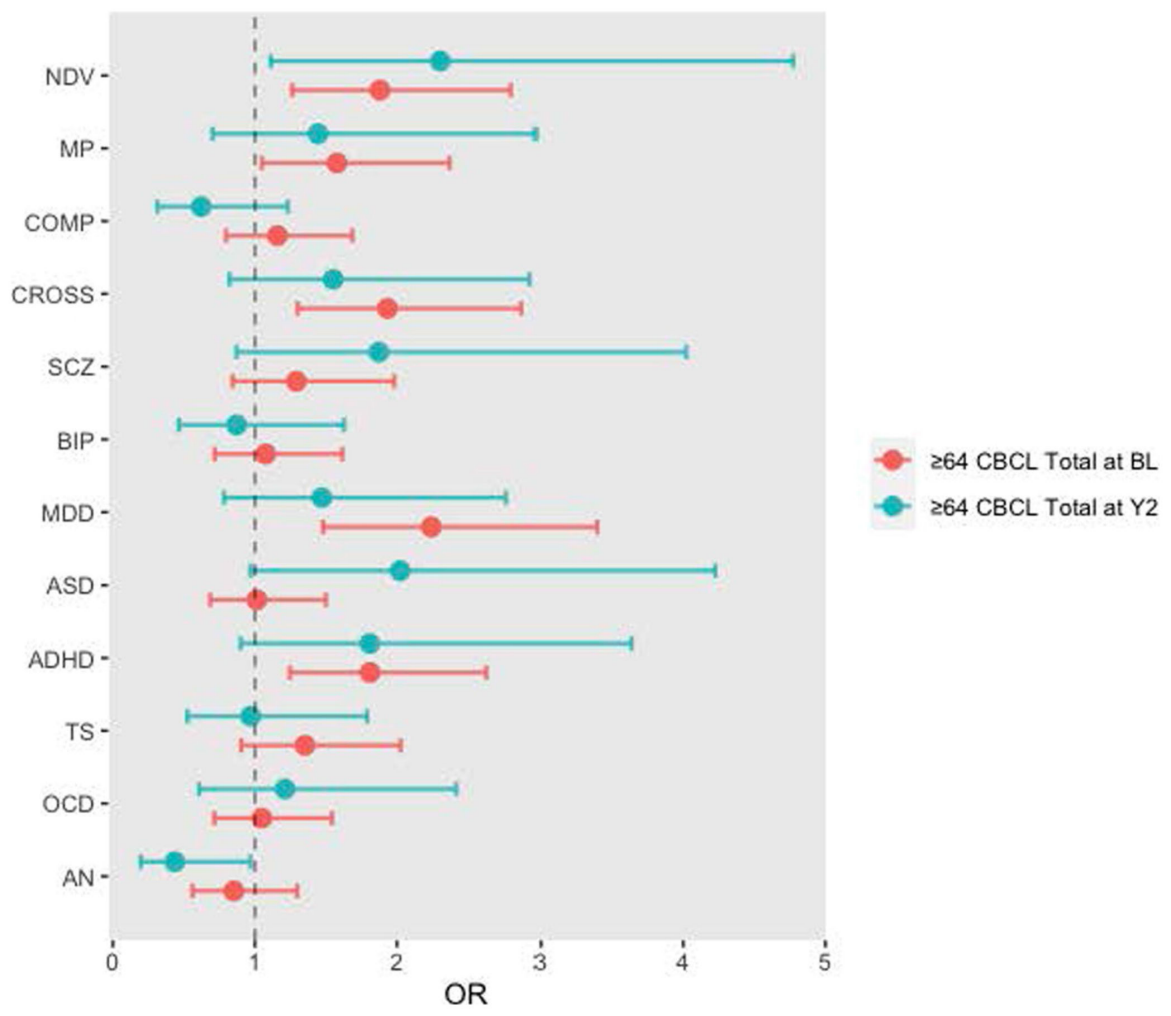
Odds of clinical-range psychopathology (CBCL Total score ≥64) comparing the top to the bottom quintiles of PGS. Red represents odds of clinical-range psychopathology scores at baseline (age 9-10; n = 4,462). Blue represents odds of clinical-range psychopathology scores at year 2 (age 11-12) but not baseline (age 9-10; n = 3,152). Linear mixed effects regressions (two-sided) are adjusted for age, sex, and the top 5 genetic PCs as fixed effects, and site as a random effect. Points represent estimated odds ratios and error bars indicate 95% confidence intervals around those estimates.

Further, given the associations of NDV PGS with continuous measures of psychopathology, we also tested the relative strength of NDV PGS in predicting psychopathology that is strong enough to fall within the clinical range in the ABCD cohort. Of all disorder-specific and cross-disorder PGSs, when comparing the top with bottom quintiles, NDV PGS was the only measure of genetic risk that was consistently associated with higher odds of endorsing both psychopathology within the clinical range (CBCL Total score ≥ 64 (ref. 34)) at baseline (odds ratio (OR) = 1.88; 95% confidence interval (95% CI), 1.26–2.80; *P* = 0.002) and newly emergent clinical-range psychopathology at age 11–12 (OR = 2.30; 95% CI, 1.11–4.77; *P* = 0.02), although the age 11–12 result would not survive false discovery rate (FDR) correction (Extended Data Fig. 3).

### NDV gene ontology and spatiotemporal expression in brain tissue

**Fig. 3:**
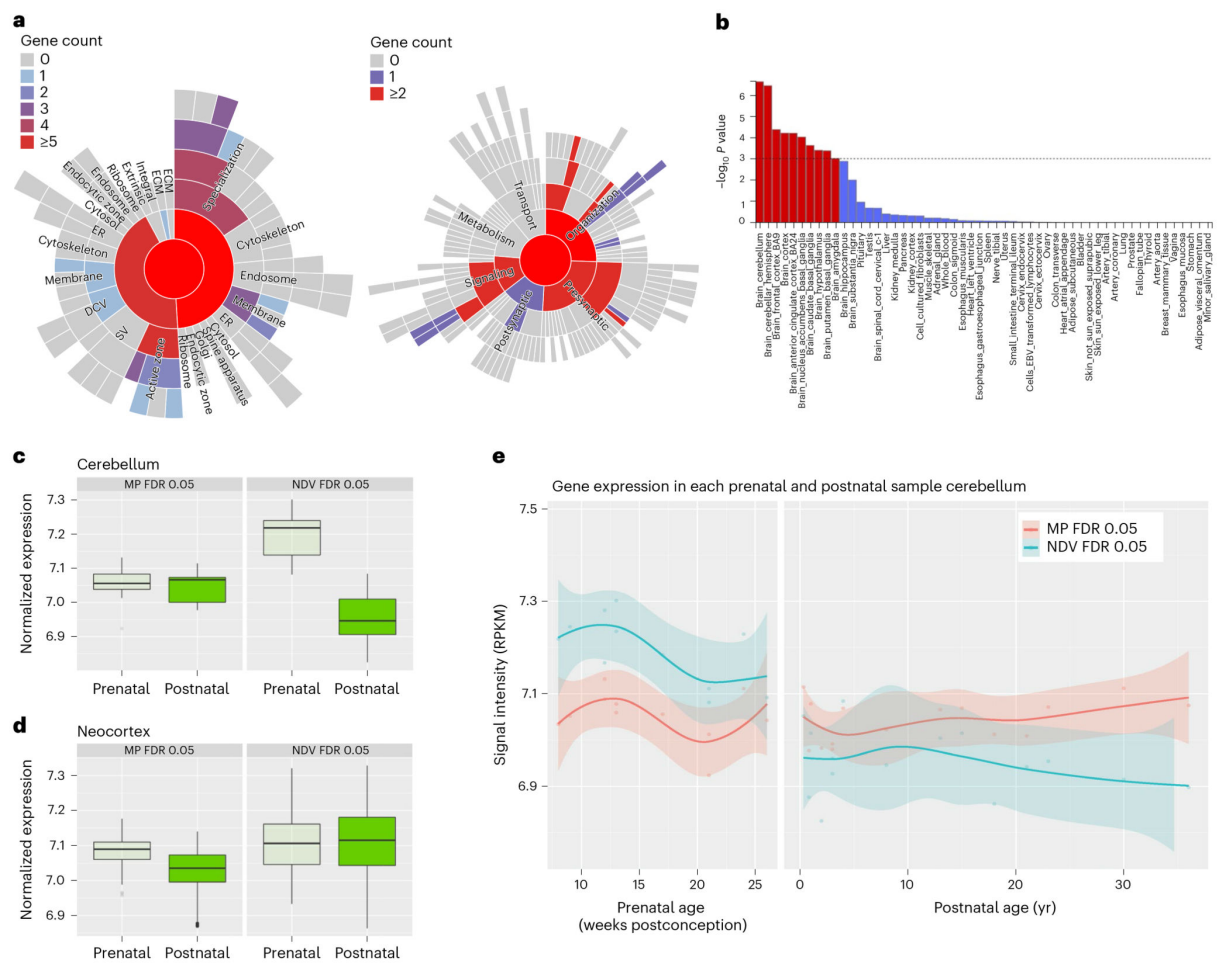
Spatial and temporal NDV gene expression. **a**, SynGO expression profile of NDV genes indicating enrichment for synaptic (and primarily presynaptic) neuronal processes. **b**, Plot of results from MAGMA gene property regression (one-sided) analysis showing significance levels (uncorrected, log-transformed, *y* axis) of each region tested (*x* axis; *n* = 17,265 genes across 53 tissue types). Horizontal dashed line indicates Bonferroni-corrected significance threshold (0.05/53). **c**,**d**, Boxplots comparing prenatal with postnatal expression of FDR-significant (*Q* <0.05) MP (left; *n* = 2,751 genes) and NDV (right; *n* = 68 genes) genes in the cerebellum (**c**) and neocortex (**d**). Boxes represent the interquartile range (IQR), lines within the boxes the median, whiskers the IQR × 1.5 and points the outliers. **e**, Spline graphs comparing NDV (blue) and MP (red) gene expression across the lifespan in the cerebellum. Shaded regions represent standard error. DCV, neuronal dense core vesicle; ECM, extracellular matrix of the synpatic cleft; ER, presynaptic endoplasmic reticulum; RPKM, reads per kilobase million; SV, synaptic vesicle.

We next leveraged gene ontology (GO) and postmortem gene expression databases to explore biological pathways through which NDV genes could impart risk for childhood psychopathology. After annotation^35^ of NDV single nucleotide polymorphisms (SNPs) to nearby genes, of 19,052 genes tested, 68 genes were significantly enriched for NDV SNPs after FDR correction (*Q* < 0.05; Supplementary Table 10). Although GO analyses of these 68 significant genes yielded no FDR-significant GO terms, GO cellular component analysis of the top 5% most significant genes (*P* < 0.014, *Q* < 0.291, *N* = 952) indicated enrichment for synaptic processes, mostly localized in dendritic and neuron spines (Supplementary Table 11). Similar results were seen in sensitivity analyses that included the top 2% and top 10% most significant genes (Supplementary Table 12). Follow-up GO analyses focusing specifically on synaptic processes (SynGO)^36^ most strongly implicated presynaptic terms (Fig. 3a). In contrast, among 2,571 genes that were significantly (*Q* < 0.05) enriched for MP SNPs, SynGO revealed a preponderance of postsynaptic terms (Supplementary Fig. 3a). Only eight genes mapped onto COMP-associated SNPs (*Q* < 0.05), likely reflecting smaller sample sizes in PGC GWASs that load onto this factor. As such, subsequent analyses focused on NDV and MP genes.

We next used FUMA (Functional Mapping and Annotation of GWAS)^37^ in conjunction with Genotype-Tissue Expression (GTEx) v8 (ref. 38) gene expression data to compare tissue-specific expression of NDV and MP genes. Genes harboring NDV SNPs were most strongly expressed in the cerebellum (*P* = 2.14 × 10^-7^), followed by cerebral cortical and subcortical regions (Fig. 3b). In contrast, genes harboring MP SNPs were most strongly expressed in the cerebral cortex, followed by cerebellar and subcortical regions (Supplementary Fig. 3b).

**Extended Data Fig. 4:**
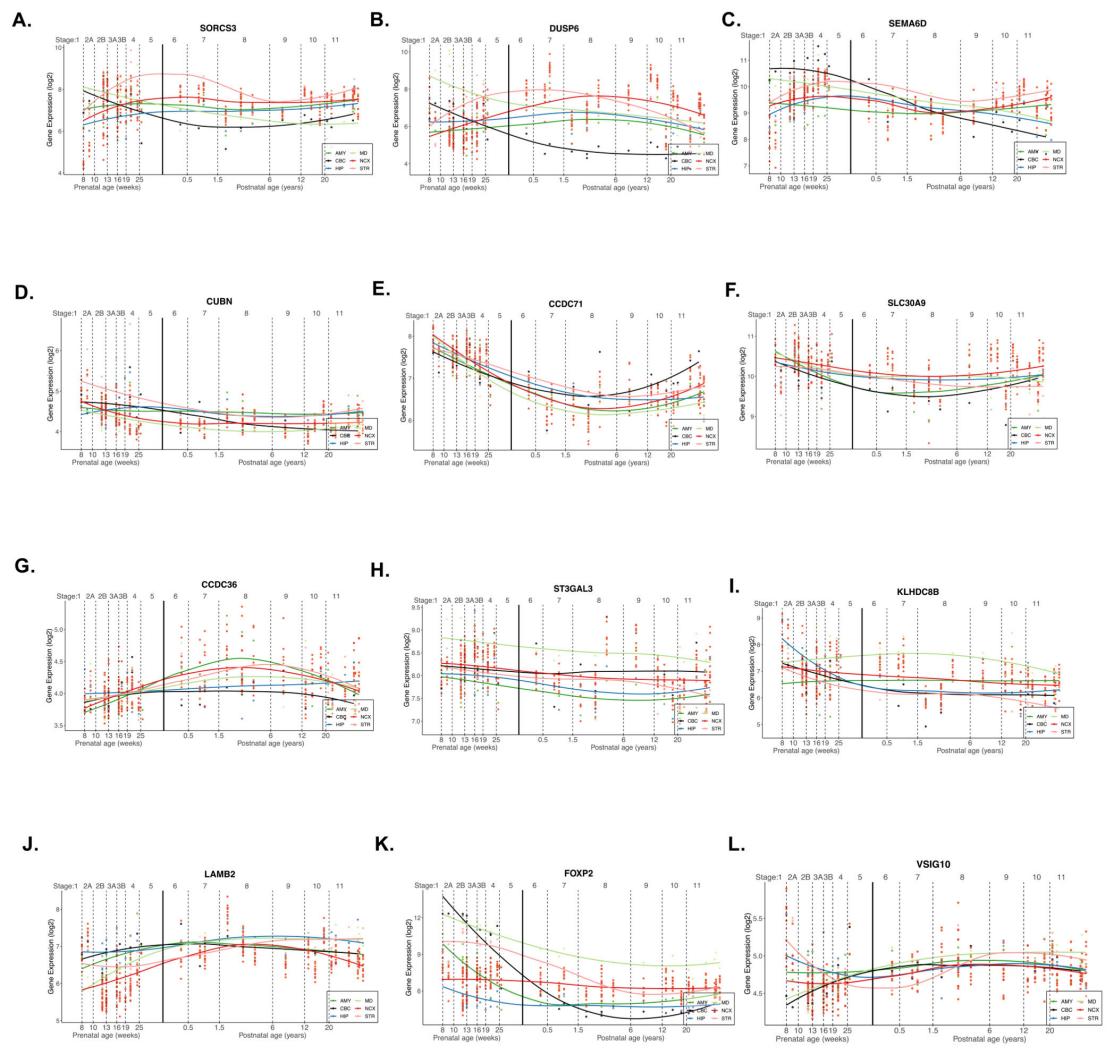
Regional gene expression patterns across the lifespan. Depicted are expression patterns of 12 of the most significant NDV genes (q < 0.009) using gene expression data from BrainSpan. Each plotted line represents expression across the lifetime within 1 of 6 regions (one color per region; black represents expression in the cerebellum). Vertical black line represents the delineation between prenatal and postnatal timepoints. Abbreviations: AMY, amygdala; CBC, cerebellar cortex; HP, hippocampus; MD, mediodorsal thalamus; NCX, neocortex; STR, striatum. (**a**, *SORCS3*; **b**, *DUSP6*; **c**, *SEMA6D*; **d**, *CUBN*; **e**, *CCDC71*; **f**, *SLC30A9*; **g**, *CCDC36*; **h**, *STGAL3*; **i**, *KLHDC8B*; **j**, *LAMB2*; **k**, *FOXP2*; **l**, *VSIG10*).

To assess temporal patterns of NDV and MP gene expression within the cerebellum, and to conduct exploratory analyses in other brain regions, we used BrainSpan^39^ data, contrasting tissue obtained postmortem from fetal brain versus postnatal brain tissue in six brain regions. Within the cerebellum, FDR-significant NDV genes (*N* = 68) were expressed significantly more strongly before birth than after birth (*P* = 8.68 × 10^-08^; Fig. 3c,e). Across five other cortical and subcortical regions, expression levels of NDV genes also differed between pre- and postnatal timepoints in the mediodorsal nucleus of the thalamus and the striatum (Supplementary Table 13). Conversely, MP genes were more highly expressed before birth than after birth in all regions assessed (for example, neocortex; Fig. 3d) except for the cerebellum (Fig. 3c) after correcting for multiple comparisons (uncorrected *P* values < 0.001; *P*_cerebellum_ = 0.56) (Supplementary Table 13). Additionally, we inspected regional expression patterns of individual genes across the lifespan for top NDV genes (Extended Data Fig. 4). Two genes, *SEMA6D* and *FOXP2*, which have been implicated in the etiologies of neuropsychiatric disorders^11,40^, show marked differences in pre- and postnatal expression in the cerebellum (Extended Data Fig. 4).

### Associations of gene expression patterns with symptoms

**Fig. 4:**
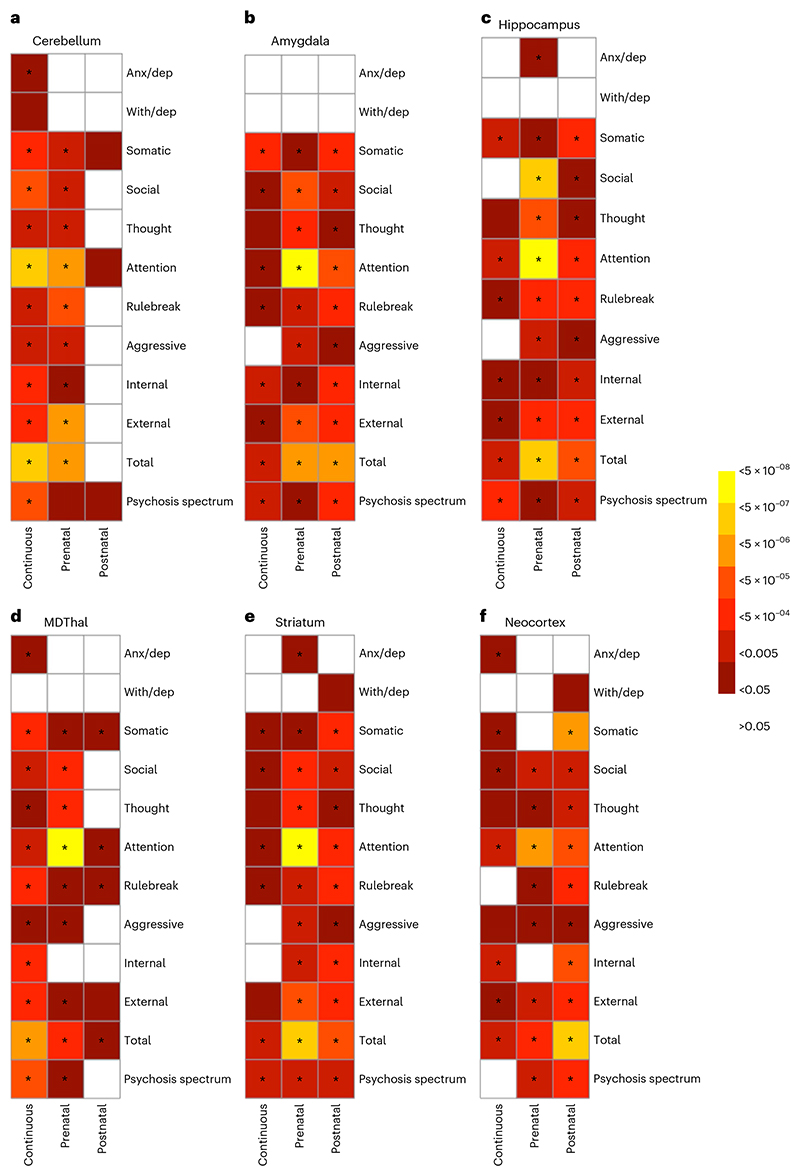
Tissue-specific effects of NDV pPGS, based on gene sets with prenatal peak, postnatal peak or continuous gene expression, on dimensional psychopathology. Linear mixed effects regressions are adjusted for age, sex and the top five genetic principal components (PCs) as fixed effects, and site as a random effect. *P*values shown are two-sided and uncorrected. Stars indicate *P* <0.05 after FDR correction for 36 comparisons (3 pPGSs × 12 measures of psychopathology). White boxes represent nonsignificant relationships (*P*>0.05). Panels represent NDV pPGS partitioned based on expression in the cerebellum (**a**), amygdala (**b**), hippocampus (**c**), medial dorsal thalamus (**d**), striatum (**e**) and neocortex (**f**).

We next determined whether relationships between variation in NDV genes and dimensional symptoms were conditional on the developmental timing of cerebellar gene expression. First, we parsed all genes with available expression data into three groups based on expression levels in the cerebellum: those that show FDR-significant peaks in expression (1) before birth (*N* = 3,506 genes) and (2) after birth (*N* = 4,025 genes), and (3) those that do not significantly differ in expression between pre- and postnatal timepoints (*N* = 10,073 genes). Then, we generated partitioned NDV PGSs (pPGSs) from each of these sets of genes and tested their association with psychopathology. Genes that were primarily expressed before birth in the cerebellum associated significantly with various CBCL scores (*Q* values 2.55 × 10^-05^ to 0.019); genes that showed comparable pre- and postnatal expression levels exhibited similar associations with CBCL as well as PQ-BC (*Q* values 2.75 × 10^-06^ to 0.030; Fig. 4a and Supplementary Table 14). Conversely, PGSs calculated with genes expressed after birth in the cerebellum did not significantly predict CBCL scores (*Q* values > 0.05). In contrast, cumulative effects of postnatal NDV genes on CBCL were comparable to those of prenatal NDV genes within other subcortical structures and the neocortex (Fig. 4b–f).

### Relationship of CBCL scores to cerebellar volumes

**Extended Data Fig. 5:**
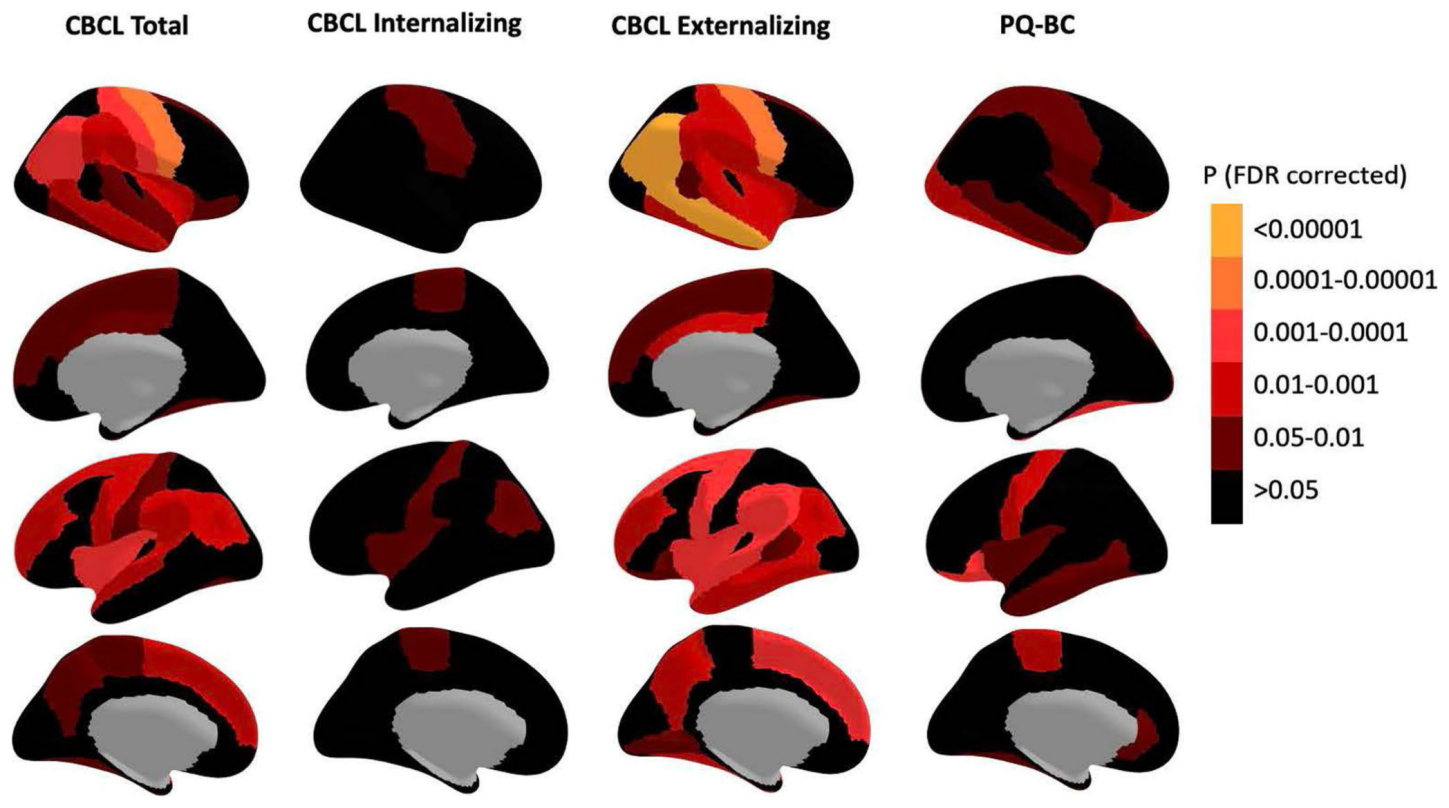
Effects of cortical ROI volumes on dimensions of psychopathology. Linear mixed effects regressions (two-sided) are adjusted for age, sex, intracranial volume, and Euler number as fixed effects, and site, scanner, and family ID as random effects. Warmer colors represent more significant associations. P-values are corrected at the False Discovery Rate (number of comparisons = 272 [68 regions × 4 scales]).

**Fig. 5:**
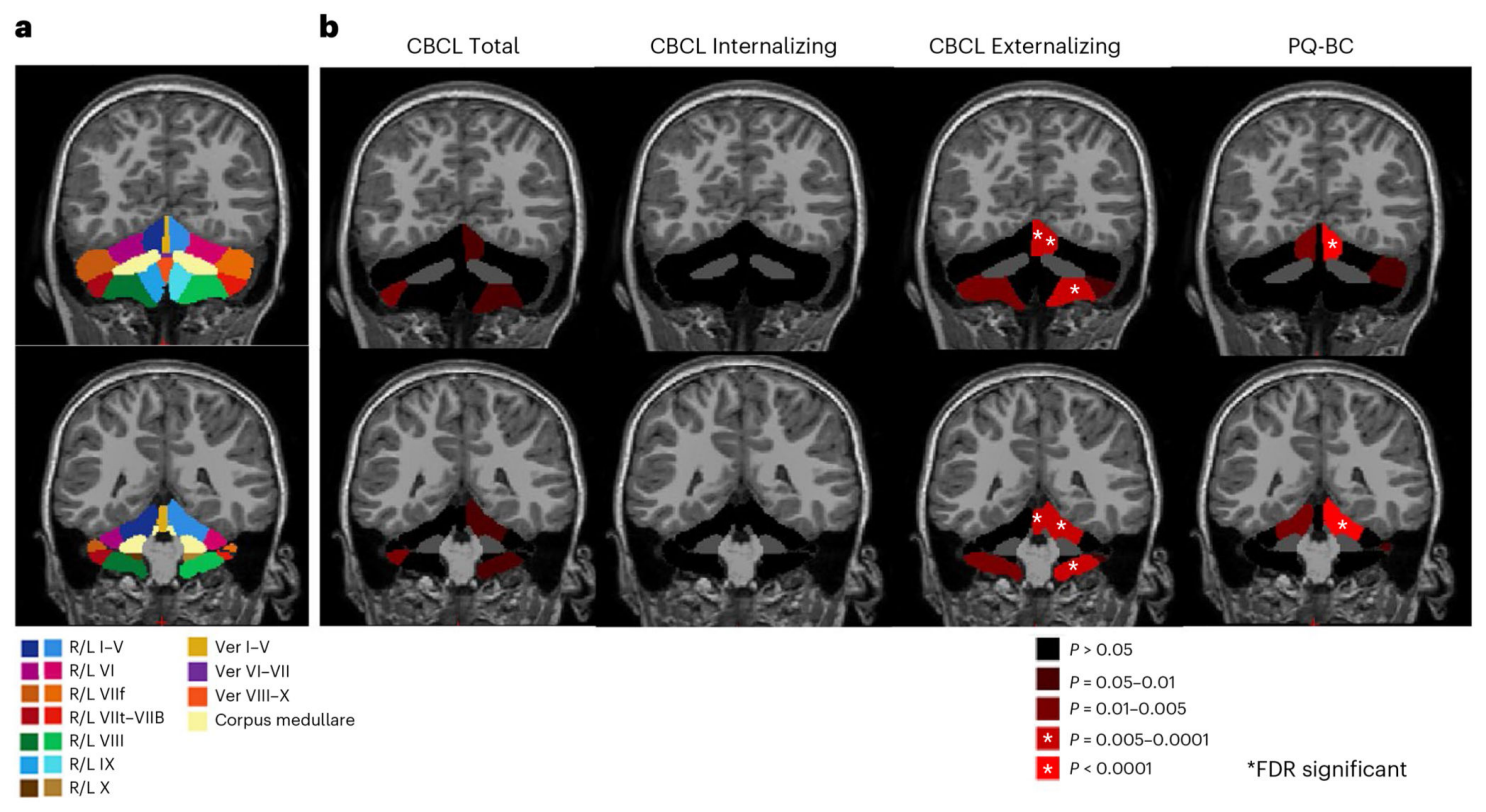
Association between cerebellar volumes and dimensional psychopathology. **a**, Cerebellar lobule map with legend for reference. Each lobule is represented by a different color (see key below). **b**, Effects of cerebellar volume on dimensional psychopathology: from left to right, CBCL Total, Internalizing, Externalizing and PQ-BC. Brighter reds indicate more significant associations with stars indicating regions that showed statistical significance after correction for multiple comparisons (FDR). Linear mixed effects models were adjusted for age, sex, intracranial volume and Euler number as fixed effects, and site, scanner and family ID as random effects. R/L, right/left; Ver, vermis.

Baseline T1 MRI scans from all ABCD participants underwent rigorous visual quality control, resulting in retention of 3,878 scans from the unrelated European ancestry group, and 10,076 scans overall. Total cerebellar and cerebellar subregion volumes were determined after segmentation using Automatic Cerebellum Anatomical Parcellation Using U-Net with Locally Constrained Optimization (ACAPULCO) software, which has been validated in previous pediatric cohorts^41^. We then examined relationships among NDV PGSs, cerebellar volumes, and broader CBCL scores and PQ-BC. In the European ancestry group, neither NDV PGS nor NDV pPGS associated significantly with global brain volume measures (total brain, cortical, subcortical and cerebellar gray matter volumes; Supplementary Table 15). However, in the larger group (that is, not restricted to participants of European ancestry), total cerebellar gray matter volume was negatively associated with CBCL Total (*P* = 0.012) and Externalizing (*P* = 1.23 × 10^-4^) scores (Supplementary Table 16). Among specific cerebellar lobule volumes, right lobules VIIt–VIIB had the strongest inverse association with CBCL Total scores, although this relationship did not survive multiple testing correction (*β* (beta coefficient estimate) = -0.035, *P* = 0.006, *Q* > 0.05). Volumes of the left lobules I–V (*β* = -0.43, *P* = 6.25 × 10^-4^, *Q* < 0.05) and VIII (*β* = -0.46, *P* = 1.88 × 10^-04^, *Q* < 0.05) and Vermis I–V (*β* = -0.38, *P* = 0.002, *Q* < 0.05), which are anterior regions that show functional coupling to somatomotor and association cortex^42^, exhibited the strongest inverse associations with CBCL Externalizing score (Fig. 5, Supplementary Fig. 4 and Supplementary Table 17). Exploratory analyses testing for associations between dimensional psychopathology and cortical and subcortical volumes outside of the cerebellum revealed significant inverse correlations in a number of cortical regions, including somatomotor and association cortex, and subcortical regions, including brain stem, thalamus and hippocampus (Extended Data Fig. 5 and Supplementary Tables 18 and 19). Results from sensitivity analyses including subjects with the highest quality scans (that is, those rated as ‘1’ or ‘2’ per MRI data quality control in the Methods, *N* = 8,658) are reported in Supplementary Table 20.

## Discussion

In this study we found that dimensional psychopathology in children is most strongly related to an NDV PGS comprising overlapping genetic variants across ADHD, ASD, MDD and Tourette syndrome. NDV scores explained more variance across the spectrum of psychopathology than any other disorder-specific or cross-disorder measure of genetic risk. Longitudinal data demonstrate stable and replicable effects of NDV PGS on psychopathology in early adolescence. Further, convergent data from complementary GO, gene expression and MRI datasets link presynaptic effects of NDV genes in the fetal cerebellum to downstream clinical effects. Collectively, these findings suggest a mechanism through which altered fetal cerebellar development instantiates risk for a wide range of childhood psychopathology.

These findings are consistent with growing evidence for the dimensional underpinnings of psychopathology and of the heritable, developmental origins of neuropsychiatric illness. Research studies of child psychopathology increasingly rely on dimensional scales reflecting deviation from age-related norms. As such, PGSs representing shared risk among psychiatric disorders may be well-suited to track with risk for emergent psychiatric illness. Previous studies, including an analysis of age 9–10 ABCD data, have reported significant, but weaker, effects of disorder-specific PGSs on dimensional psychopathology in mid-childhood^17,18,28,43^. The present results indicate that polygenic risk models accounting for overlapping risk among NDV disorders are better suited to capturing psychopathology that occurs in childhood. For example, while psychosis spectrum symptoms were unrelated to SCZ PGS in both the ABCD and Generation R cohorts (consistent with some previous studies of children and adolescents^18,44^), they were significantly predicted by NDV PGS (at both ages in ABCD, and at age 13 in Generation R). This pattern contrasts with that seen in adults, where disorder-specific PGSs most strongly predict risk for their respective clinical syndromes despite extensive pleiotropy observed across major psychiatric disorders^45^. As participants in the ABCD Study approach adulthood, and psychopathology becomes further differentiated, it will be of interest to follow whether disease-specific polygenic models account for more variance in symptoms than they do at the outset of adolescence. Prospectively observing when, and in whom, clinical sequelae of generalized (NDV) loading are supplanted by disorder-specific PGSs may ultimately lead to refined predictive algorithms for youth who show nonspecific early symptoms.

NDV PGSs were derived empirically from pooled GWAS data that cover eight psychiatric disorders, and represent overlapping genetic risk among ADHD, ASD, Tourette syndrome and MDD. While the first three of these disorders are typically diagnosed in childhood, MDD also presents frequently in children, with approximately one-fifth of children aged 12–17 reporting a major depressive episode^46^. Compared with later in adolescence, early childhood depression reflects stronger genetic overlap with ADHD, and more frequently co-occurs with language and communication traits seen in autism^47^. Further, MDD PGSs derived from adult-sample GWASs consistently associate not only with internalizing symptoms, but also with ADHD, social problems and overall psychopathology in other large cohorts of children^29^. GWASs of MDD also implicate NDV processes^48^. However, in the present results, NDV PGS predicted more variance in dimensional depression scores than did MDD PGS, and overall predicted more variance in most aspects of dimensional psychopathology compared with disorder-specific PGSs. Additional analyses suggested that NDV likely contributes to this range of symptoms in early adolescence through its effects on ‘p’, that is, a general factor of psychopathology, rather than through direct effects on differentiated symptoms— and thus suggest the possibility that these symptoms reflect common biological substrates that act downstream of NDV genes.

While genes annotated to NDV-associated variants are expressed throughout the brain, they are most strongly expressed within the fetal cerebellum. The role of the cerebellum in the emergence of psychopathology has received increased attention over the past two decades^49–53^. To our knowledge, relationships between cerebellar volumes and dimensional psychopathology have not previously been examined in the ABCD Study. Of note, the relatively high spatial variability of small cerebellar lobules across individuals necessitates the use of specialized, probabilistic atlases to resolve lobular volume artifacts^54^. For the present analysis, we used a cerebellar atlas that has been validated in children. Also, robust visual quality control of all individual MRI scans (Methods) enabled the elimination of scans with substantial artifacts that were not detected by standard quality control measures in Freesurfer, as well as inclusion of individual scan quality ratings for those images that were deemed sufficient for inclusion.

That NDV genes were preferentially expressed in the cerebellum echoes previous, smaller studies that found associations between cerebellar gray matter morphology and general psychopathology, norm-violating behaviors and psychosis through late adolescence^51^. In the present analysis, NDV scores predicted the same three measures of psychopathology above all other measures of genetic risk, and in the larger ABCD sample, more strongly predicted externalizing psychopathology and psychosis than internalizing psychopathology at both timepoints. Further, while cerebellar morphometry negatively predicted total psychopathology, replicating findings of several studies^51,52^, the present analyses also demonstrate that volumes within specific cerebellar subregions more strongly predicted externalizing and psychotic symptoms compared with total symptoms.

Altered cerebellar development has been linked to numerous neuropsychiatric syndromes in children^55^, but as in other previous work^56^, in the present study structural variation in extracerebellar cortical and subcortical regions also associated with psychopathology scores, as did variation in NDV genes expressed in these regions. Effects of fetally expressed NDV genes within the cerebellum may propagate over space and time, including via extracerebellar regions that contribute to psychopathology risk. The cerebellum is synaptically and functionally coupled to all major brain networks via cerebellum– thalamic– cortical loops^42^ and modulates gain for motor, cognitive and emotional function^57^. Disruption in cerebellar connectivity to cortical regions has been implicated in SCZ and autism^58,59^. It has been proposed that fetal cerebellar development influences postnatal maturation of multiple cortical regions^60^. As such, disrupted fetal cerebellar development may exert downstream effects on cortical maturation that are relevant to psychiatric symptoms^60,61^. In support of this idea, we found that reduced volumes in somatomotor regions of the cerebellum (for example, lobules I–V, VIII), as well as in networked regions within the sensorimotor cortex, associated with increased externalizing symptoms, a pattern consistent with previous findings^62^. The present results implicate NDV genes in this process, in particular to the extent that their expression in fetal cerebellum influences externalizing symptoms through downstream effects on cortical development. Longitudinal follow-up of ABCD participants may identify structural and functional variations in cerebellar and networked regions that associate with, and potentially precede, changes in symptoms over time.

The present analyses did not identify relationships between NDV scores and gray matter volumes, despite the statistically robust relationships between NDV PGSs and dimensional psychopathology measures. Recent large-scale MRI studies have highlighted the need for very large sample sizes to avoid type I and type II error in relating psychopathology to anatomical and functional brain variation^63,64^. Likewise, very large samples—possibly larger than ABCD—may be needed to link MRI indices and psychopathology with underlying genomic risk. Alternatively, as NDV genes tend to show peak cerebellar expression during fetal life, PGSs arising from these genes may track more closely with cerebellar volumes during fetal development than in childhood. However, the finding that cerebellar gray matter volumes cross-sectionally predicted psychopathology scores in early adolescence suggests the possibility that NDV gene expression during fetal life exerts developmentally downstream effects on cerebellar structure that become relevant to emerging psychopathology after birth. Future studies that provide detailed spatiotemporal gene expression data from human cerebellum, drawing from recent studies in mice^65^, may enable closer triangulation among intracerebellar NDV expression, cerebellar lobule volumes and emergent pediatric psychopathology.

Several limitations of the current study may be addressed in future analyses. First, the largest gene expression dataset used in our analysis (GTEx v8), which we used to identify the cerebellum as a region-of-interest, is derived largely from adults^38^. Therefore, although NDV genes are likely relevant for cerebellar function, we are unable to determine with the same confidence their importance to cerebellar development. However, as described above, top (FDR-significant) NDV genes are expressed significantly more during the prenatal period than after birth, suggesting that they have some relevance to early cerebellar development. Related to this issue, with the two available gene expression datasets (GTEx v8 and BrainSpan), we are limited in the number of brain regions in which we can investigate the expression patterns of genes. Thus, we are unable to systematically assess the relative importance of NDV gene expression in the cerebellum compared with other regions of the brain. Although the ABCD Study itself is designed to reproduce the diversity of the US population, our current genomic analyses were restricted to non-Hispanic participants of European descent. This approach was necessitated by the risk of population stratification artifacts when pooling data from participants of mixed ancestry. The field of psychiatric genomics is making strides towards greater diversity^66^, but sample sizes of both non-European discovery GWAS datasets and related analyses in ABCD are likely underpowered at present. As such, the generalizability of the present genomic findings to non-European ancestry individuals remains limited. Finally, the data reported here are limited to early adolescence, rely to some extent on parent-reported data and exclude children with serious mental illness (for example, SCZ, severe autism). Our analysis of 2-yr follow-up data (ages 11–12) showed similar patterns of association with PGSs to those seen in ages 9–10. Nevertheless, the next several years of life, characterized by substantial biological and social changes, will likely bring about further phenotypic differentiation. Continued observation of the ABCD participants will enable a fuller view of the dynamics of psychopathology over adolescence, and a more complete understanding of how emergent psychopathology tracks with genetic variation and neuroanatomical development.

### Online content

Any methods, additional references, Nature Portfolio reporting summaries, source data, extended data, supplementary information, acknowledgements, peer review information; details of author contributions and competing interests; and statements of data and code availability are available at https://doi.org/10.1038/s41593-023-01321-8.

## Methods

### ABCD Study

The ABCD Study includes data from 11,875 children aged 9–10 at baseline with the intention of following them through adolescence. All data were obtained from the National Institute of Mental Health (NIMH) Data Archive (NDA), Curated Annual Release 4.0 using the NDA Download Manager Beta (v.1.39.0). General inclusion and exclusion criteria for the ABCD Study are described elsewhere^67,68^. In brief, 9–10-yr-old children were recruited from the community, had no contraindications to MRI scanning and were excluded if they: were not fluent in English; had a history of major neurological disorders, traumatic brain injury or extreme prematurity; or carried a diagnosis of SCZ, moderate-to-severe ASD, intellectual disability or substance use disorder. Institutional Review Board (IRB) approval for the ABCD Study is described by Auchter et al.^69^. Most ABCD research sites cede approval to a central IRB at the University of California, San Diego, with the remainder obtaining local IRB approval. All parents provided written, informed consent and all youth provided assent. Unless explicitly noted, all of the below methods describe analyses performed with ABCD data. For instructions on gaining access to ABCD data, refer to this page: https://nda.nih.gov/nda/access-data-info.html. To request access to Generation R data, researchers can email: datamanagementgenr@erasmusmc.nl.

### Measures of psychopathology

Data from the CBCL and distress scores from the PQ-BC were analyzed to assess psychopathology at baseline (ages 9–10) and year 2 follow-up (ages 11–12). The CBCL is reported by parents and consists of 11 scales, three of which capture broader psychopathology—Total, Internalizing and Externalizing— and eight of which capture more specific syndromes of psychopathology—anxious/depressed, withdrawn/depressed, somatic complaints, social problems, thought problems, attention problems, rule-breaking behavior and aggressive behavior. The PQ-BC is a modified version of the Prodromal Questionnaire Brief and establishes the presence or absence of symptoms of prodromal psychosis as reported by the child. A rating of distress for each endorsed item is also recorded on a scale of 1–5. For the present analysis, total distress scores were calculated by summing the distress and endorsement scores for all 21 questions for each individual. In Generation R, comparable measures of psychopathology were used including the same eight syndrome and three broadband CBCL scales. Because the PQ-BC was not collected in Generation R, consistent with a previous study^23^, psychotic experiences were evaluated using two items on auditory and visual hallucinations from the Youth Self-Report^34^: (1) ‘I hear sounds or voices that are not there according to other people’ and (2) ‘I see things that other people think are not there’. Children responded on a three-point scale: not at all (0), a bit (1) or clearly (2). The sum score of the two hallucination items was calculated, and children were grouped into three different categories: no symptoms (0 points), mild symptoms (score of 1 point on at least one of the items) and moderate-to-severe symptoms (score of 2 points on at least one of the items).

### Bifactor analyses of CBCL

Bifactor analyses followed from Clark and colleagues^33^, who analyzed baseline CBCL data from the ABCD Study. Factor loadings from two models, GFP-2 and GFP-3, were used to provide scores for nine-factor (‘p’ as well as residualized eight CBCL syndrome-specific scales) and three-factor (‘p’ as well as residualized CBCL Internalizing and Externalizing scales) models, respectively, for each ABCD participant based on their age 9–10 individual item data.

### Quality control and imputation of genetic data

Genotype data from nontwin individuals of self-reported European ancestry were retained for analysis. To minimize family-level confounders, one child was randomly selected for analysis from every sibling pair. All subsequent pre- and postimputation quality control analyses were conducted using PLINK v.1.9. SNPs with a minor allele frequency (MAF) of less than 1%, with missingness greater than 5% and with Hardy–Weinberg equilibrium less than 1 × 10^-5^, were filtered. Variants in linkage disequilibrium were pruned using a window size of 50 kilobases (kb), a step size of 5 kb and an *R*^2^ threshold of 0.5. A principal components analysis was then run to calculate the first four principal components and to filter individuals who fell outside 4 s.d. from the mean of each of the four principal components, calculated in a European reference population via the 1000 Genomes Project^70^. Individuals were removed who had an identity-by-descent value greater than 0.125, a sex mismatch or who were missing more than 5% of their data. Shapeit v.2 was used for prephasing, with genotyping data from the 1000 Genomes project used as a reference panel. IMPUTE v.2 was used for imputation.

### Polygenic scoring analysis using ABCD data

After imputation, variants were filtered for an INFO score of less than 0.9, a MAF < 0.01 and missingness >0.05. PGSs were calculated by summing the loci associated with risk of a particular trait weighted by their effect size on that trait. Using PRSice-2 (ref. 71) and an *R*^2^ threshold of 0.1 to clump SNPs in linkage disequilibrium, PGSs were calculated for eight specific psychiatric disorders—ADHD, ASD, anorexia nervosa, BIP, MDD, OCD, SCZ and Tourette syndrome—the summary statistics of which were obtained from the PGC and can be found here: https://www.med.unc.edu/pgc/download-results/. These disorders were selected in line with the recent PGC Cross-Disorder Group (CDG) paper, which identified cross-trait risk loci across these eight disorders^21^. Following up on further analyses from the CDG, PGSs were also derived from three latent factors that together accounted for 59% of the genetic variation among the eight neuropsychiatric disorders. A liberal *P* value inclusion threshold of 1.0 was selected for subsequent PGS analyses to include all SNP effects and to be consistent across the dimensions of psychopathology tested; however, predictive power (represented as *R*^2^ change) at each inclusion threshold can be seen in Fig. 2, Supplementary Figs. 1 and 2 and Extended Data Fig. 2c,d. To determine whether PGS effects on psychopathology were driven by GWAS discovery sample sizes, we plotted each GWAS sample size on the corresponding PGS effect estimate from models regressing CBCL Total on PGS (Supplementary Fig. 5).

### Sensitivity analysis using PGSs calculated with PRS-CS

As a sensitivity analysis, we regenerated PRSs using a Bayesian approach (PRS-CS^32^). Due to the relatively small sample sizes (<200,000) and high polygenicity of the tested psychiatric traits, *ϕ*, the global shrinkage parameter, was set at 0.02 for all traits except CROSS, for which *ϕ* was learned from the data. The remainder of the parameters were kept at the default setting. In PLINK, the resultant posterior effect sizes were applied to individual-level genotype data to generate PGSs for each subject. Results from these analyses are reported in Supplementary Table 5.

### GenomicSEM analysis to infer latent factors of major psychiatric disorders

The GenomicSEM package in R was used for factor analyses and subsequent calculations of summary statistics of each factor. To verify the genetic architecture outlined by the CDG, a genetic covariance matrix (*S*) and sampling covariance matrix (*V*) were calculated for the eight neuropsychiatric disorders by the linkage disequilibrium score regression (LDSC) method of the GenomicSEM packages. The *S* matrix was then used for an exploratory factor analysis with three factors and promax rotation. Excluding factor loadings of less than 0.2, a latent structure was identified that accounted for 59% of the genetic variation and fell into three categories as previously defined by the CDG: an NDV factor comprising ADHD, ASD, MDD and Tourette syndrome; an obsessive/compulsive factor (COMP) comprising anorexia nervosa, OCD and Tourette syndrome; and an MP factor comprising BIP, MDD and SCZ. A follow-up confirmatory factor analysis with three correlated factors suggested a good model fit with *χ*^2^(15) = 78.88, Akaike information criteria (AIC) = 120.88, comparative fit index (CFI) = 0.940, standard root mean square residual (SRMR) = 0.077. With three latent structures having been identified in three clusters of psychiatric disorders, common factors of each cluster were regressed onto each SNP via the commonfactorGWAS function. Thus, a set of summary statistics, representing SNP effects on their respective factors, were generated for the NDV, COMP and MP factors separately. Before generating factor summary statistics, all PGC summary statistics were standardized and preprocessed via the sumstats function.

### Gene-based association analyses

Gene-based association analyses were performed through MAGMA^35^ via FUMA’s^37^ pipeline, which is a web-based software. SNPs within a window of 35 kb upstream and 10 kb downstream of a gene were annotated to their corresponding genes. With the European 1000 Genomes Project Phase 3 data as a reference panel and *P* values from summary statistics of each of the three gSEM factors, the effect and statistical significance of each gene on its corresponding phenotype were calculated using an SNP-wise mean model, which uses the mean *χ*^2^ statistic of SNPs in a gene to calculate the effect of the gene^35^. Gene expression data from GTEx v.8 were used for tissue specificity analyses.

### GO analyses

Using PANTHER^72^, a web-based software, GO analyses were performed on two sets of NDV genes: (1) FDR-significant genes (*N* = 68) and (2) top 5% most significant genes (*N* = 952, *Q* < 0.3). Specifically, we used PANTHER’s overrepresentation test^73^ with Fisher’s exact test for *P* value calculation to test which GO terms were overrepresented in each set of genes. *P* values were corrected for multiple comparisons with FDR. Three classes of GO terms were tested: cellular components, which identifies cellular locations in which products of genes of interest are active; molecular function, which defines the biochemical activity of gene products; and biological process, which refers to the process to which a gene product contributes^74^. The FDR-significant set of genes (*N* = 68) yielded no significant results, and thus we explored a larger set of genes (*N* = 952) using PANTHER. In addition, given the likely role of synaptic processes in the etiology of psychopathology^6,7,75,76^, we investigated the initial set of 68 FDR-significant genes using SynGO, a web-based GO software with gene annotations specifically related to synapse biology^36^.

### Developmental gene expression trajectories of psychiatric risk genes

Using data from the BrainSpan Atlas of the Developing Brain, expression levels were assessed for genes that were identified by the MAGMA annotation analyses and that statistically significantly contributed risk to the gSEM-derived latent variable (*Q* < 0.05). For top NDV (*N* = 68) and MP (*N* =2,751) genes, mixed models were used to identify structures that showed differential gene expression patterns before and after birth. Given within-gene dependence of expression, each gene was allowed its own intercept; likewise, each donor was allowed to have their own intercept to preserve within-donor relationships. The main effect of developmental time window (postnatal = 0, prenatal = 1) on expression was used to determine pre- and postnatal differential expression within each brain structure. See the formula below modeling the effect of developmental time window on gene expression of gene *i* within donor *j*:

$$\begin{array}{c} {\text{exp}}_{ij}\;=\;\beta_{0ij}+\beta_{1}\text{postnatal}+\varepsilon_{ij}\\ \beta_{0ij}=\gamma_{00}+\mu_{0ij} \end{array}$$

Given results from MAGMA’s gene property analysis via the FUMA pipeline pointing to the importance and relevance of the cerebellum to NDV genes, the main structure of interest was the cerebellum (CBC). In addition, five other brain structures were tested: amygdala (AMY), medial dorsal thalamus (MDTHAL), striatum (STR), hippocampus (HIP) and neocortex (NCX), which was an aggregate of regions: primary auditory cortex (A1C), dorsolateral prefrontal cortex (DFC), inferior parietal cortex (IPC), inferolateral temporal cortex (ITC), primary motor cortex (M1C), rostral medial prefrontal cortex (MFC), orbital frontal cortex (OFC), primary somatosensory cortex (S1C), caudal superior temporal cortex (STC), primary visual cortex (V1C) and ventrolateral prefrontal cortex (VFC), in addition to occipital neocortex (Ocx), parietal neocortex (PCx), temporal neocortex (TCx) and primary motor-sensory cortex (M1C-S1C) as defined on the Allen Brain Atlas site (atlas.brain-map.org). BrainSpan data can be downloaded here: https://www.brainspan.org/static/download.html.

### pPGSs

All genes (*N* = 17,604) were partitioned into those with prenatal peak expression, postnatal peak expression and continuous expression (showing no expression differences between pre- and postnatal timepoints), using independent samples *t*-tests that contrasted mean pre- versus postnatal expression. After correction for multiple comparisons with FDR (number of comparisons = 17,604), genes with a significant positive estimate were classified as prenatal genes, with a significant negative estimate as postnatal genes and with an insignificant positive or negative estimate as continuous. These tests were performed within each of the aforementioned six regions to identify prenatally, postnatally and continuously expressed genes within each brain region. Using MAGMA’s SNP-to-gene annotation data to convert gene-level data back to SNP-level data, SNPs were classified in the same way (prenatal, postnatal and continuous within six regions). These data were then used to generate subsets of NDV summary statistics and subsequently PGSs which represent the additive effects of SNPs that confer risk to the NDV and which are also associated with genes that are expressed differentially or non-differentially between fetal and child/adult timepoints. These analyses resulted in 18 new PGSs (3 (pre-, post-, continuous) × 6 regions (CBC, AMY, MDTHAL, STR, HIP, NCX)). Psychopathology scores were then regressed onto these PGSs, allowing for an inspection of the importance of timing of expression on emergent psychopathology in pre-adolescence.

### Polygenic scoring analysis using Generation R data

Using imputed genotype data from previous Generation R studies^77^ and summary statistics from the gSEM output generated from the present study, PGSs were calculated for each of the three gSEM factors.

### MRI data processing-FreeSurfer

In the ABCD Study, structural T1 images were acquired on 3T scanners (1 × 1 × 1-mm^3^ resolution)^78^. To correct low frequency intensity nonuniformity, also known as a bias field, we used the N4 bias field correction algorithm^79^. Whole brain processing and analyses, including generation of global and region-of-interest (ROI) volumes, were conducted using FreeSurfer v.7 (http://surfer.nmr.mgh.harvard.edu/).

### MRI data quality control

Of 11,875 participants, 160 did not have T1-weighted images available to download. A total of 11,715 images were downloaded, 451 of which were flagged to receive clinical consultation and thus were excluded from visual quality control, and one of which failed FreeSurfer preprocessing. The remaining 11,263 images (4,242 from unrelated European participants (‘uEur’)) were individually assessed and given a rating from a scale of 1–5. The rating criterion was created based on degree of manual edits needed. A rating of 1 was given to scans that required only minor manual edits that could be completed within approximately 0.5 hours (*n* = 4,630 total, 1,610 uEur). A rating of 2 was given to scans that required several manual edits but could still be completed within approximately 1–2 h (*n* = 4,063 total, 1,636 uEur). A rating of 3 was given to scans with a larger number of manual edits needed that would take more than 3–4 h (*n* = 1,383 total, 632 uEur). A rating of 4 was given to scans with severe motion and other types of artifacts that might not be possible to fix with manual edits (*n* = 219, 48 uEur). The remainder of the scans were unusable, had gross anatomical abnormalities or had cysts > 1 cm^3^ (*n* = 968 total, 87 uEur). Only images that were rated as 1, 2 or 3 (*n* = 10,076 total, *n* = 3,878 uEur) were used in subsequent analyses. For cerebellar subregion analyses, subjects who fell outside of 4 s.d. of the mean total cerebellum volume (*n* = 19) were excluded from analyses. Furthermore, to apply as stringent quality control as possible, we included a Freesurfer-generated measure of scan quality in all neuroimaging models: Euler number^80^, which indexes the number of topological defects, or surface holes, in Freesurfer’s reconstruction of the cortical surface and which has been shown previously to act as a metric of scan quality^81^. The number of surface holes was used as a fixed effect in all imaging analyses.

### Cerebellum segmentation with ACAPULCO

We used ACAPULCO for cerebellum subregion analysis^41^. ACAPULCO was selected among other cerebellum parcellation software due to its previous validation in pediatric cohorts^82^. With ACAPULCO, FreeSurfer-preprocessed T1 images automatically went through N4 bias field correction, Montreal Neurological Institute (MNI) registration, cerebellum parcellation (the program first predicts a bounding box around the cerebellum, crops out the cerebellum with this bounding box, and uses a modified U-Net to parcellate it into subregions) and transform back to the original space. Then, volume for each subregion was calculated.

### Statistical analyses

All statistical analyses were performed for ABCD data with R 3.6.3 and for Generation R data with SPSS. All statistical tests were two-sided.

#### Correlation between measures of psychopathology

Pearson *r* correlation coefficients were calculated for relationships between the 12 measures of psychopathology (11 CBCL scales and PQ-BC) and presented as a correlation matrix (Fig. 1). The same was done in Generation R, although a different metric of psychosis spectrum symptoms was included instead of the PQ-BC, which was not collected in Generation R.

#### Associations between PGS and dimensional psychopathology

Due to variance both within and between the 22 ABCD Study sites, linear mixed effects models (lme4 package) were used to adjust for site as a random effect. In models investigating the effects of eight disorder-specific, one broad cross-disorder and three gSEM-derived PGSs on indices of psychopathology, age at baseline, sex and the top five principal components were included as fixed effects and site as a random effect. In Generation R, because data were collected from one site only, simple linear regressions (that is, without random effects) were used to calculate main effects of the three gSEM-derived PGSs, controlling for age, sex and the top five principal components.

#### PGS associations with baseline and emergent clinically meaningful psychopathology

To determine the relevance of NDV polygenic risk on the emergence of psychopathology relative to other disorder-specific and cross-disorder scores, subjects were partitioned into quintiles based on their PGSs and coded according to presence of psychopathology above the clinical cutoff for CBCL Total (≥64)^34^. In baseline models (Extended Data Fig. 3), two groups were identified: one with CBCL Total scores at or above the clinical cutoff at baseline and another with scores below. In year 2 models, two groups were identified: one with CBCL Total scores at or above the clinical cutoff at year 2, but not baseline; another with scores below the cutoff at both baseline and year 2. Logistic regression models were used to determine the odds of having clinical-range psychopathology (CBCL Total ≥ 64) given membership in the top versus bottom PGS quintile.

#### Associations between PGS, psychopathology and brain structure

In models including imaging and genomics data from unrelated participants of European ancestry with scan ratings of 1, 2 or 3, site and scanner type were included as random effects and additionally the number of surface holes (Euler number) was included as a fixed effect, as were age, sex and intracranial volume. In the broader imaging set, which included participants of multiple ancestry groups and also 2,820 sibling participants, PGSs and principal components were omitted, but family ID was included as an additional random effect, as well as Euler number as a fixed effect. The FDR was used to correct for multiple comparisons within each set of models, which were treated hierarchically. First, four global measures of gray matter volume (total gray matter, cerebellum total gray matter, cortical gray matter and subcortical gray matter) were each tested for association with four clinical scales (CBCL Total, Internalizing and Externalizing, as well as PQ-BC), and FDR was used to correct for 16 comparisons (Supplementary Table 16). For global gray matter volumes that showed significant associations with any clinical scale, follow-up tests were conducted that corrected for the total number of subregions and clinical scales (that is, 17 cerebellar subregions × 4 clinical scales = 68 comparisons, Supplementary Table 17; 68 cortical regions × 4 clinical scales = 272 comparisons, Supplementary Table 18; 17 subcortical regions × 4 clinical scales = 68 comparisons, Supplementary Table 19). As a sensitivity analysis, subjects with a structural quality control rating of 3 were excluded from models, and thus associations between brain volumes and psychopathology were measured only in subjects with the highest quality scans (*N* = 8,658).

#### Proportion of variance explained by PGS

To determine the predictive power of each PGS, *R*^2^ changes were calculated for models predicting broadband CBCL scales and PQ-BC. First, changes in *R*^2^ at each PGS threshold were calculated by building an initial model consisting of covariates (without PGS of interest) and then a second model that included PGS as a predictor. The *R*^2^ values of the initial models were subtracted from those of the second models. Although the main PGSs of interest were at P-threshold (Pt) 1.0, these analyses were performed at each Pt. Next, to determine the relative predictive power of NDV scores compared with other disorder-specific and cross-disorder PGSs, two more models per PGS were built: the initial model included a PGS at Pt 1.0 and covariates; the second model added NDV PGS at Pt 1.0. The significance associated with the addition of NDV scores (that is, the *P* value of the NDV term in the model) is reported in Fig. 2c,d, Supplementary Fig. 1 and Supplementary Table 4. These same analyses were performed in Generation R (Supplementary Fig. 2, Extended Data Fig. 2c,d and Supplementary Table 8).

### Reporting summary

Further information on research design is available in the Nature Portfolio Reporting Summary linked to this article.

## Extended Data

## Supplementary Material

Supplementary Material

## Data Availability

All ABCD data are available via the NIMH Data Archive. For instructions on gaining access to ABCD data within this repository, refer to this page: https://nda.nih.gov/nda/access-data-info.html. ABCD data created in the current study can also be downloaded from the NDA (https://doi.org/10.15154/1528597). For access to the Generation R dataset, requests can be sent to datamanagementgenr@erasmusmc.nl. BrainSpan Atlas of the Developing Brain gene expression data are available through their website (https://www.brainspan.org/static/download.html); 1000 Genomes phase 3 data are available through this site: https://www.internationalgenome.org/data-portal/data-collection; and summary statistics from the Psychiatric Genomics Consortium can be downloaded here: https://www.med.unc.edu/pgc/download-results/. GTEx v.8 RNA-seq data can be analyzed through FUMA’s pipeline (https://fuma.ctglab.nl/) and the raw data downloaded here: https://gtexportal.org/home/datasets.

## Code availability

Code for generation of polygenic scores, spatiotemporal gene expression analyses, imaging analyses and PGS-psychopathology analyses is available on GitHub (https://github.com/hughesdy/ABCD-NDV-CBC).
