# Supplementary material for: Genetic patterning for child psychopathology is distinct from that for adults and implicates fetal cerebellar development
Source: Nat Neurosci. Author manuscript; Available in PMC 2023 Jul 12. (PMC7614744; doi:10.1038/s41593-023-01321-8)
Supplement: Supplementary Material [file EMS178360-supplement-Supplementary_Material.pdf]

nature neuroscience

Article

https://doi.org/10.1038/s41593-023-01321-8

# Genetic patterning for child psychopathology is distinct from that for adults and implicates fetal cerebellar development

In the format provided by the authors and unedited

**Supplemental Figure 1. Variance in psychopathology explained by 8 disorder specific and 4 cross-disorder indices of genetic risk in the ABCD cohort**. Uncorrected p-values (shown within the figure in black text near the y-max) represent the significance of the $R^2$ change when adding NDV to base mixed effects regressions including respective PGS and age, sex, and top 5 principal components as fixed effects and site as a random effect ($P_t$ =1). Details can be found in **Supplemental Table 4**. (**A**) CBCL Externalizing Symptoms at age 9-10; (**B**) CBCL Externalizing Symptoms at age 11-12; (**C**) CBCL Internalizing Symptoms at age 9-10; (**D**) CBCL Internalizing Symptoms at age 11-12; (**E**) PQ-BC at age 9-10; (**F**) PQ-BC at age 11-12. $N_{\text{Baseline}}$ = 4,459, $N_{\text{Year2}}$ = 3,360. All regressions represented are two-sided.

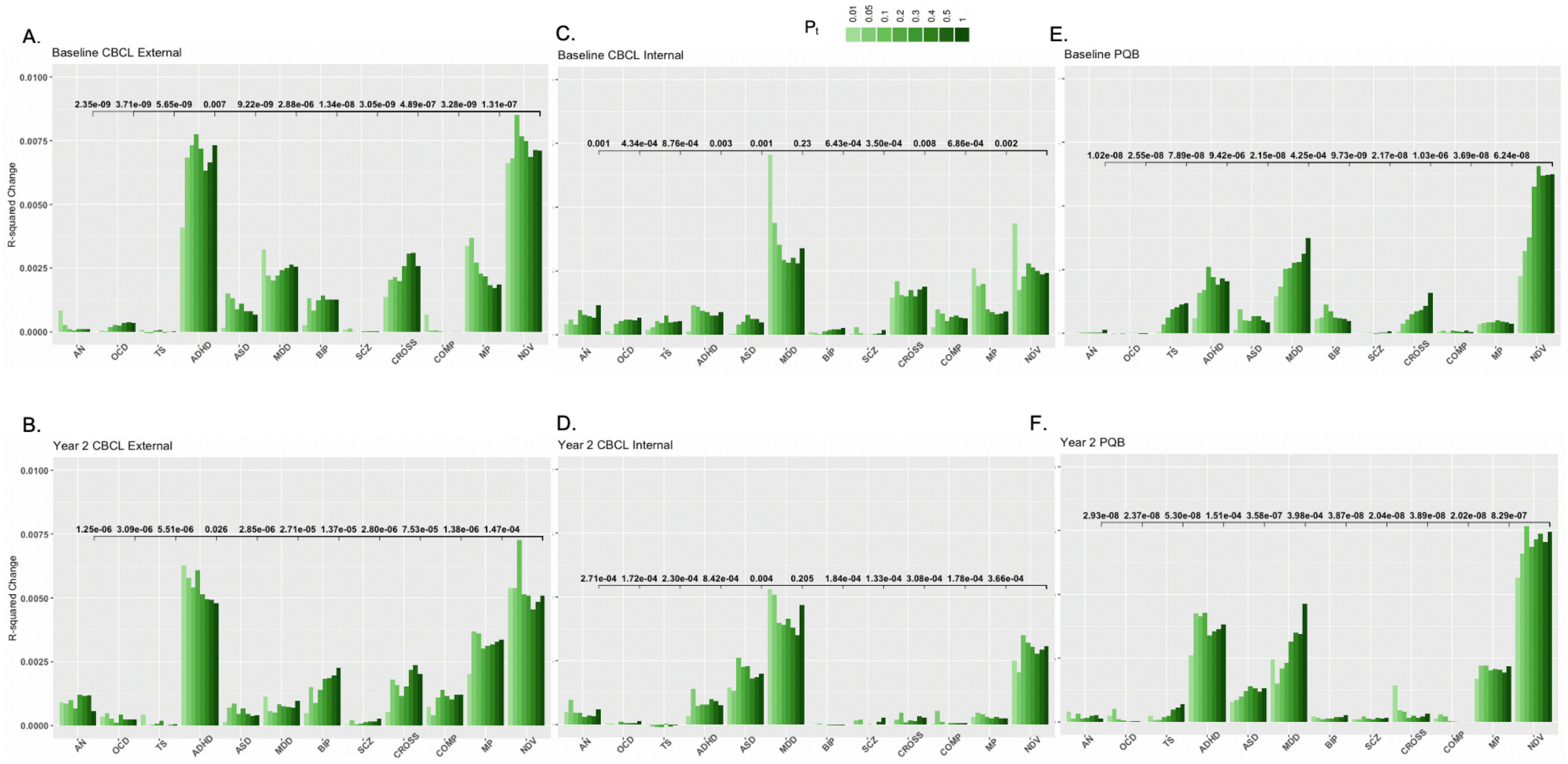

**Supplemental Figure 2. Variance in psychopathology explained by the 3 gSEM-derived PGS in the Generation R cohort.** Uncorrected p-values (shown within the figure in black text near the y-max) represent the significance of the $R^2$ change when adding NDV to base mixed effects regressions including respective PGS and age, sex, and top 5 principal components ($P_t$ =1). (**A**) CBCL Externalizing Symptoms at age 9; (**B**) CBCL Externalizing Symptoms at age 13; (**C**) CBCL Internalizing Symptoms at age 9; (**D**) CBCL Internalizing Symptoms at age 13; (**E**) psychosis spectrum at age 9; (**F**) psychosis spectrum at age 13. $N_{Age9}$ = 1,850, $N_{Age13}$ = 1,791. All regressions represented are two-sided.

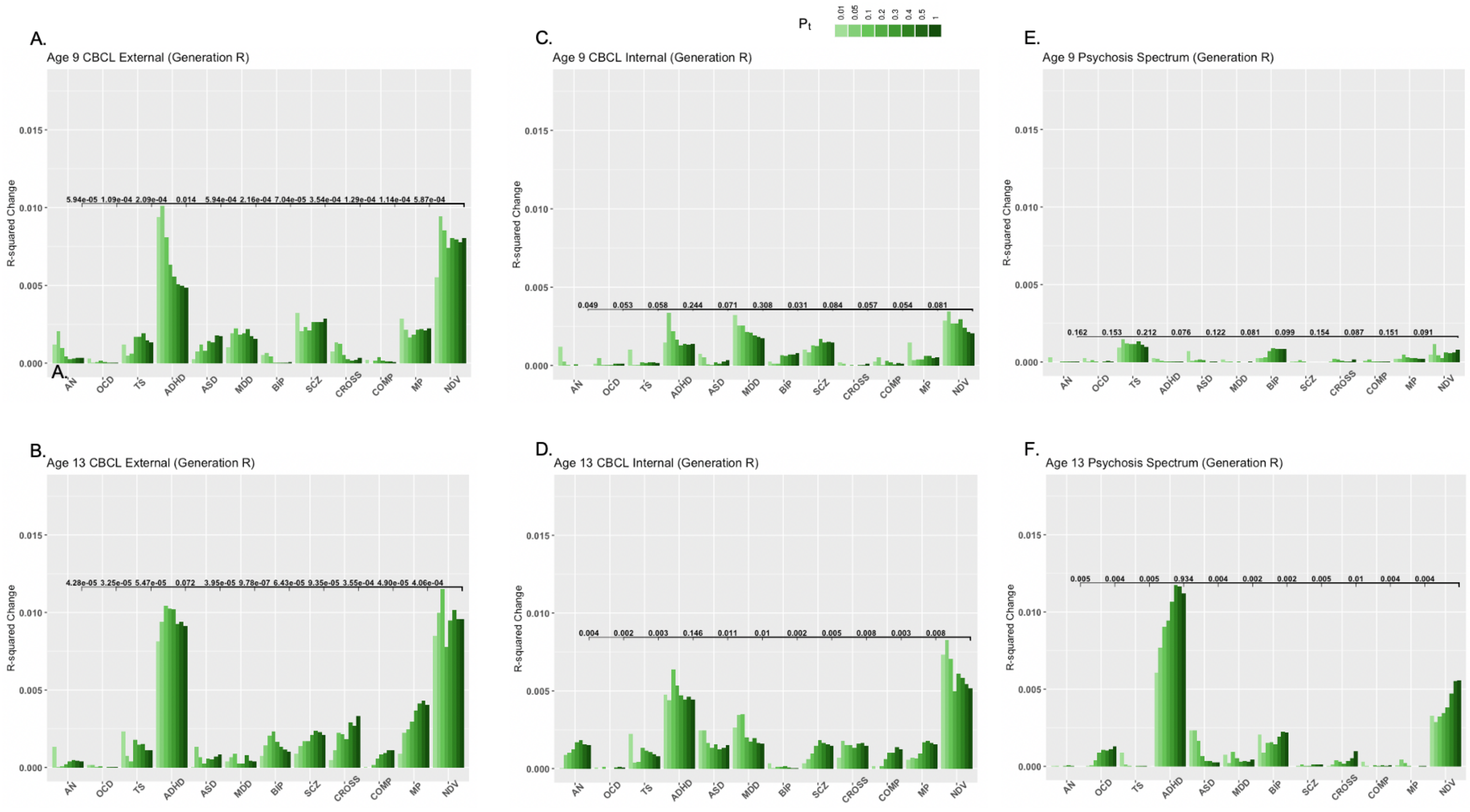

**Supplemental Figure 3. Mood/psychotic gene spatial expression and ontology results.** (**A**) SynGO expression profile of mood/psychotic genes. (**B**) Results from one-sided MAGMA gene property analysis showing significance levels (uncorrected, log-transformed, y-axis) of each region tested (x-axis; n = 17,029 genes across 53 tissue types). Horizontal dotted line indicates Bonferroni corrected significance threshold (0.05/53).

**A.**

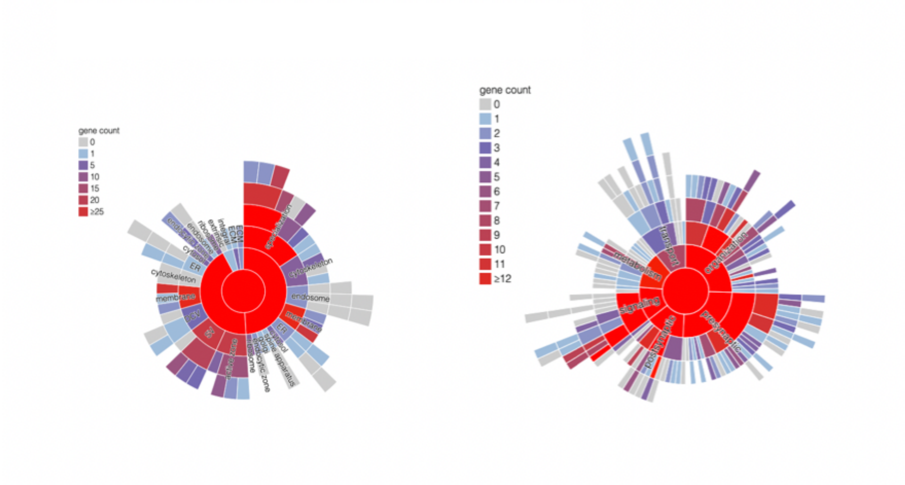


**B.**

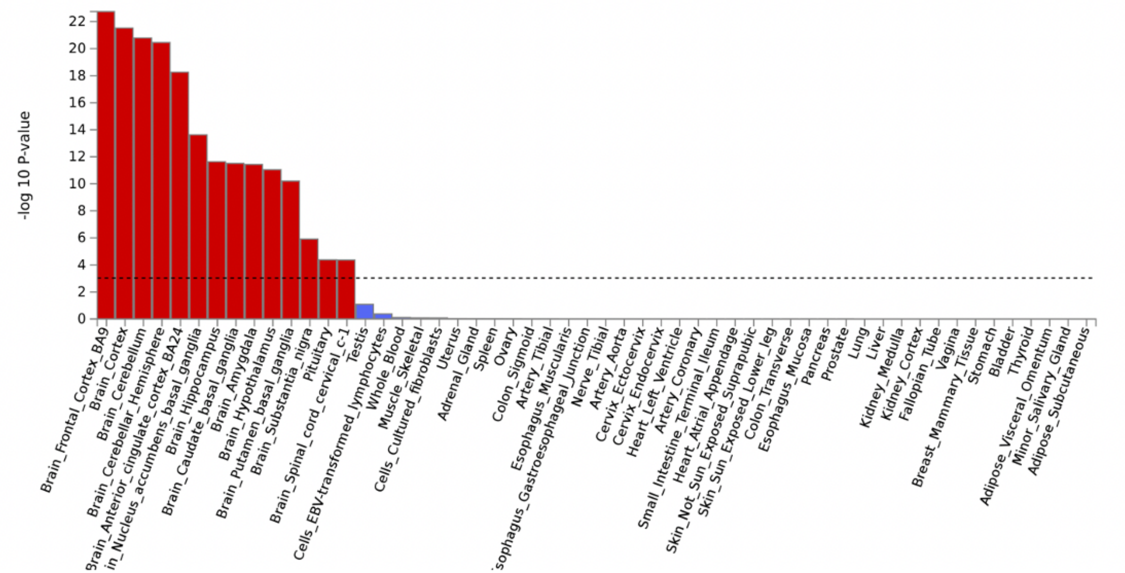

**Supplemental Figure 4. Residual scatter plots with line of best fit showing the relationship between cerebellar left lobules I-V volume and psychopathology.** (**A**) depicts CBCL Externalizing residuals on the y-axis and (**B**) depicts PQ-BC, which are the two most significant relationships among all psychopathology on cerebellar subfield models. Residuals are derived from linear mixed effects regressions adjusted for age, sex, surface holes (Euler), and intracranial volume as fixed effects, and site, scanner, and family ID as random effects.

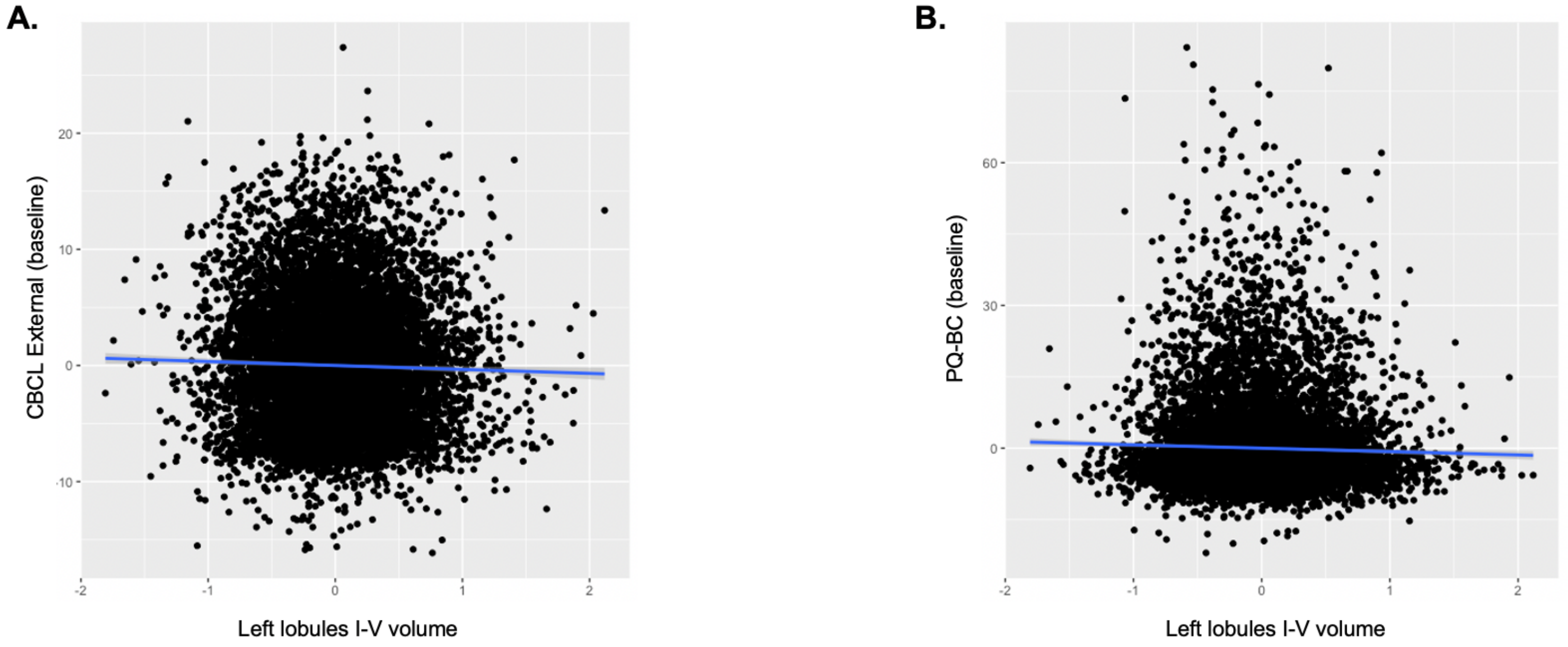

**Supplemental Figure 5. Relationship between GWAS sample sizes and corresponding PGS-CBCL Total effect sizes.** This graph plots GWAS sample size on the x-axis and ß coefficient estimates from models regressing CBCL Total on PGS on the y-axis. $R^2$ and p-value of this relationship, derived from a linear regression of effect size (CBCL Total on PGS) on GWAS sample size, is included. Error bars represent standard error around the effect estimate.

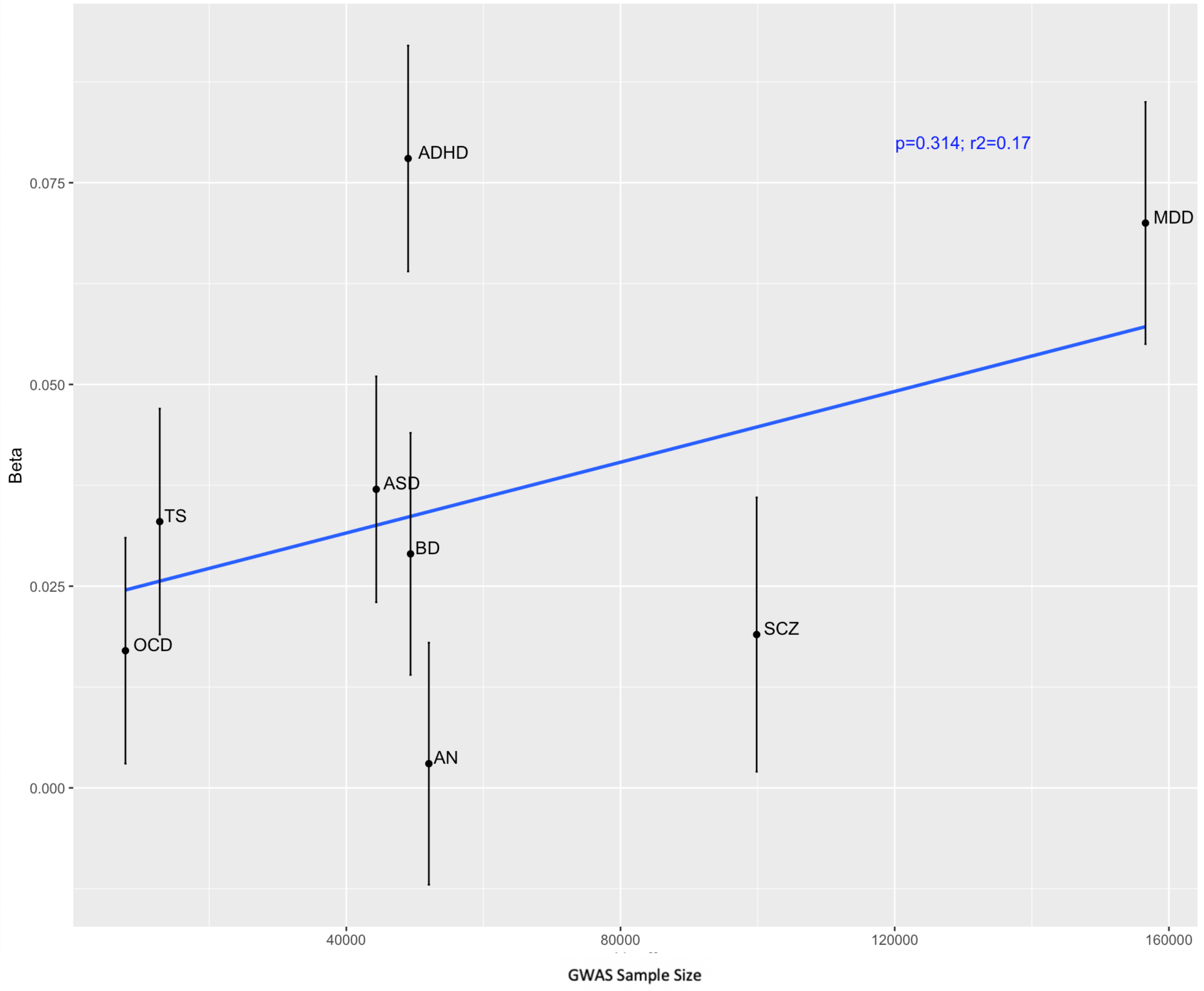

## SUPPLEMENTAL TABLE 1. Factor loadings and correlations from genomic structural equation modeling (gSEM)

Factor loadings and correlations from current analysis (**A**) and comparable results from Lee et al. 2019 (**B**)

NB: Factors are listed in order of proportion variance accounted for. As such, the order of factors (Factor 1, 2, 3) differ between ABCD and Lee et al., but they are analogous in composition (e.g., ABCD Factor 1 comprises ADHD, ASD, MDD, TS, as does Lee et al. Factor 3). Blank cells indicate positive standardized loading of <.2, per Lee et al.

**A) Results from current ABCD analysis**

| | Factor 1 (NDV) | Factor 2 (COMP) | Factor 3 (MP) |
|---|---|---|---|
| ADHD | 1 | | |
| AN | | 0.43 | |
| ASD | 0.39 | | |
| BIP | | | 0.78 |
| MDD | 0.6 | | 0.21 |
| OCD | | 1.04 | |
| SCZ | | | 0.85 |
| TS | 0.24 | 0.5 | |

FACTOR CORRELATIONS

| | Factor 1 | Factor 2 | Factor 3 |
|---|---|---|---|
| Factor 1 | | | |
| Factor 2 | 0.336 | | |
| Factor 3 | -0.428 | -0.39 | |

**B) Comparable results from Lee et al. 2019**

| | Factor 1 (COMP) | Factor 2 (MP) | Factor 3 (NDV) |
|---|---|---|---|
| ADHD | | | 0.82 |
| AN | 0.45 | | |
| ASD | | | 0.48 |
| BIP | | 0.82 | |
| MDD | | 0.24 | 0.51 |

| | | | |
|---|---|---|---|
| OCD | 1 | | |
| SCZ | | 0.81 | |
| TS | 0.45 | | 0.42 |

FACTOR CORRELATIONS

| | Factor 1 | Factor 2 | Factor 3 |
|---|---|---|---|
| Factor 1 | | | |
| Factor 2 | 0.39 | | |
| Factor 3 | -0.15 | -0.27 | |

## SUPPLEMENTAL TABLE 2. Effect sizes, and p- and q-values from CBCL-PGS mixed models at baseline (ABCD, age 9-10)

p-values in red reflect significance after FDR correction
All statistics shown are derived from two-sided linear mixed effects regressions
**N = 4459**

**Included covariates**
  Fixed effects: age, sex, top 5 principal components
  Random effects: site

FDR corrects for 12 scales x 12 PGS = 144 comparisons

| | *Beta* | *p (uncorrected)* | *p (FDR)* |
|---|---|---|---|
| **CBCL Total** | | | |
| AN | 0.003 | 0.819 | 0.867 |
| OCD | 0.017 | 0.238 | 0.357 |
| TS | 0.033 | 0.021 | 0.056 |
| ADHD | 0.078 | 4.07E-08 | 6.94E-07 |
| ASD | 0.037 | 0.009 | 0.030 |
| MDD | 0.07 | 2.07E-06 | 2.98E-05 |
| BIP | 0.029 | 0.051 | 0.117 |
| SCZ | 0.019 | 0.263 | 0.379 |
| CROSS | 0.054 | 1.92E-04 | 0.001 |
| COMP | 0.011 | 0.459 | 0.555 |
| MP | 0.045 | 0.008 | 0.027 |
| NDV | 0.09 | 3.10E-10 | 4.47E-08 |
| **CBCL External** | | | |
| AN | -0.001 | 0.947 | 0.951 |
| OCD | 0.016 | 0.280 | 0.391 |
| TS | 0.017 | 0.252 | 0.366 |
| ADHD | 0.084 | 6.84E-09 | 2.09E-07 |
| ASD | 0.027 | 0.071 | 0.140 |
| MDD | 0.055 | 3.03E-04 | 0.002 |
| BIP | 0.04 | 0.008 | 0.027 |
| SCZ | 0.038 | 0.030 | 0.074 |
| CROSS | 0.053 | 3.83E-04 | 0.002 |
| COMP | 0.003 | 0.849 | 0.886 |
| MP | 0.055 | 0.001 | 0.006 |
| NDV | 0.087 | 3.36E-09 | 1.61E-07 |
| **CBCL Internal** | | | |

| | | | |
|---|---|---|---|
| AN | 0.035 | 0.022 | 0.057 |
| OCD | 0.024 | 0.109 | 0.204 |
| TS | 0.035 | 0.019 | 0.053 |
| ADHD | 0.03 | 0.041 | 0.095 |
| ASD | 0.023 | 0.122 | 0.216 |
| MDD | 0.063 | 4.48E-05 | 3.69E-04 |
| BIP | 0.023 | 0.138 | 0.236 |
| SCZ | 0.017 | 0.351 | 0.455 |
| CROSS | 0.048 | 0.001 | 0.006 |
| COMP | 0.028 | 0.057 | 0.123 |
| MP | 0.042 | 0.016 | 0.047 |
| NDV | 0.053 | 3.97E-04 | 0.002 |
| **CBCL Attention** | | | |
| AN | -0.023 | 0.133 | 0.231 |
| OCD | 0.002 | 0.911 | 0.930 |
| TS | 0.022 | 0.129 | 0.227 |
| ADHD | 0.084 | 7.26E-09 | 2.09E-07 |
| ASD | 0.034 | 0.020 | 0.053 |
| MDD | 0.046 | 0.002 | 0.010 |
| BIP | 0.009 | 0.566 | 0.652 |
| SCZ | -0.003 | 0.848 | 0.886 |
| CROSS | 0.037 | 0.014 | 0.043 |
| COMP | -0.017 | 0.238 | 0.357 |
| MP | 0.02 | 0.250 | 0.366 |
| NDV | 0.082 | 3.04E-08 | 6.25E-07 |
| **CBCL Rulebreak** | | | |
| AN | -0.022 | 0.120 | 0.216 |
| OCD | -0.002 | 0.862 | 0.894 |
| TS | 0.014 | 0.308 | 0.414 |
| ADHD | 0.074 | 4.33E-08 | 6.94E-07 |
| ASD | 0.018 | 0.198 | 0.309 |
| MDD | 0.053 | 1.84E-04 | 0.001 |
| BIP | 0.031 | 0.030 | 0.074 |
| SCZ | 0.043 | 0.008 | 0.028 |
| CROSS | 0.048 | 0.001 | 0.003 |
| COMP | -0.008 | 0.556 | 0.646 |
| MP | 0.058 | 2.95E-04 | 0.002 |
| NDV | 0.083 | 1.44E-09 | 1.04E-07 |
| **CBCL Aggressive** | | | |
| AN | -0.002 | 0.870 | 0.895 |
| OCD | 0.016 | 0.279 | 0.391 |
| TS | 0.021 | 0.150 | 0.255 |
| ADHD | 0.059 | 3.96E-05 | 3.56E-04 |
| ASD | 0.012 | 0.405 | 0.508 |
| MDD | 0.028 | 0.064 | 0.133 |

| | | | |
|---|---|---|---|
| BIP | 0.028 | 0.064 | 0.133 |
| SCZ | 0.022 | 0.196 | 0.309 |
| CROSS | 0.048 | 0.001 | 0.005 |
| COMP | 0.007 | 0.645 | 0.720 |
| MP | 0.033 | 0.053 | 0.118 |
| NDV | 0.06 | 3.60E-05 | 3.45E-04 |
| **CBCL Anx/Dep** | | | |
| AN | 0.016 | 0.326 | 0.430 |
| OCD | 0.034 | 0.031 | 0.074 |
| TS | 0.039 | 0.014 | 0.043 |
| ADHD | 0.011 | 0.476 | 0.567 |
| ASD | 0.014 | 0.373 | 0.476 |
| MDD | 0.038 | 0.020 | 0.053 |
| BIP | 0.025 | 0.120 | 0.216 |
| SCZ | 0.032 | 0.083 | 0.162 |
| CROSS | 0.043 | 0.006 | 0.023 |
| COMP | 0.038 | 0.015 | 0.045 |
| MP | 0.052 | 0.005 | 0.018 |
| NDV | 0.03 | 0.054 | 0.118 |
| **CBCL With/Dep** | | | |
| AN | -0.006 | 0.695 | 0.752 |
| OCD | 0.027 | 0.068 | 0.138 |
| TS | 0.01 | 0.499 | 0.589 |
| ADHD | 0.011 | 0.448 | 0.547 |
| ASD | 0.027 | 0.065 | 0.134 |
| MDD | 0.043 | 0.004 | 0.016 |
| BIP | 0.001 | 0.931 | 0.945 |
| SCZ | 0.007 | 0.699 | 0.752 |
| CROSS | 0.027 | 0.069 | 0.138 |
| COMP | 0.018 | 0.215 | 0.332 |
| MP | 0.017 | 0.310 | 0.414 |
| NDV | 0.037 | 0.013 | 0.040 |
| **CBCL Somatic** | | | |
| AN | 0.021 | 0.174 | 0.284 |
| OCD | 0.008 | 0.611 | 0.692 |
| TS | 0.029 | 0.054 | 0.118 |
| ADHD | 0.045 | 0.002 | 0.010 |
| ASD | 0.018 | 0.234 | 0.357 |
| MDD | 0.07 | 4.92E-06 | 6.33E-05 |
| BIP | 0.006 | 0.685 | 0.752 |
| SCZ | -0.001 | 0.951 | 0.951 |
| CROSS | 0.044 | 0.003 | 0.013 |
| COMP | 0.006 | 0.700 | 0.752 |
| MP | 0.028 | 0.105 | 0.199 |
| NDV | 0.06 | 5.52E-05 | 4.18E-04 |

| | | | |
|---|---|---|---|
| **CBCL Social** | | | |
| AN | -0.014 | 0.367 | 0.472 |
| OCD | 0.008 | 0.571 | 0.653 |
| TS | 0.023 | 0.118 | 0.216 |
| ADHD | 0.053 | 1.92E-04 | 0.001 |
| ASD | 0.017 | 0.242 | 0.359 |
| MDD | 0.06 | 4.61E-05 | 3.69E-04 |
| BIP | 0.016 | 0.293 | 0.397 |
| SCZ | -0.014 | 0.426 | 0.529 |
| CROSS | 0.031 | 0.031 | 0.074 |
| COMP | 0.004 | 0.759 | 0.810 |
| MP | 0.011 | 0.512 | 0.600 |
| NDV | 0.066 | 5.27E-06 | 6.33E-05 |
| **CBCL Thought** | | | |
| AN | -0.022 | 0.174 | 0.284 |
| OCD | 0.014 | 0.350 | 0.455 |
| TS | 0.035 | 0.024 | 0.060 |
| ADHD | 0.062 | 3.31E-05 | 3.40E-04 |
| ASD | 0.026 | 0.094 | 0.180 |
| MDD | 0.038 | 0.016 | 0.047 |
| BIP | 0.017 | 0.271 | 0.387 |
| SCZ | -0.009 | 0.638 | 0.718 |
| CROSS | 0.05 | 0.001 | 0.005 |
| COMP | 0.013 | 0.382 | 0.483 |
| MP | 0.019 | 0.291 | 0.397 |
| NDV | 0.061 | 6.33E-05 | 4.56E-04 |
| **Psychosis Spectrum** | | | |
| AN | -0.01 | 0.442 | 0.544 |
| OCD | 0.005 | 0.677 | 0.749 |
| TS | 0.03 | 0.019 | 0.053 |
| ADHD | 0.043 | 0.001 | 0.003 |
| ASD | 0.018 | 0.171 | 0.284 |
| MDD | 0.059 | 7.85E-06 | 8.70E-05 |
| BIP | -0.017 | 0.190 | 0.304 |
| SCZ | 0.011 | 0.474 | 0.567 |
| CROSS | 0.038 | 0.003 | 0.013 |
| COMP | 0.014 | 0.285 | 0.394 |
| MP | 0.02 | 0.181 | 0.293 |
| NDV | 0.072 | 2.46E-08 | 5.90E-07 |

**SUPPLEMENTAL TABLE 3. Effect sizes, and p- and q-values of PGS-CBCL mixed models at year 2 (ABCD, age 11-12)**

p-values in red reflect significance after FDR correction

All statistics shown are derived from two-sided linear mixed effects regressions

**N = 3360**

**Included covariates**

Fixed effects: age, sex, top 5 principal components

Random effects: site

FDR corrects for 12 scales x 12 PGS = 144 comparisons

| | *Beta* | *p (uncorrected)* | *p (FDR)* |
|---|---|---|---|
| **CBCL Total** | | | |
| AN | -0.015 | 0.395 | 0.555 |
| OCD | -0.001 | 0.944 | 0.958 |
| TS | 0.029 | 0.086 | 0.175 |
| ADHD | 0.072 | 1.63E-05 | 1.95E-04 |
| ASD | 0.05 | 0.003 | 0.015 |
| MDD | 0.07 | 5.19E-05 | 0.001 |
| BIP | 0.035 | 0.042 | 0.101 |
| SCZ | 0.016 | 0.415 | 0.564 |
| CROSS | 0.053 | 0.002 | 0.009 |
| COMP | -0.016 | 0.348 | 0.501 |
| MP | 0.052 | 0.008 | 0.031 |
| NDV | 0.092 | 3.19E-08 | 1.56E-06 |
| **CBCL External** | | | |
| AN | -0.026 | 0.150 | 0.266 |
| OCD | -0.018 | 0.284 | 0.431 |
| TS | 0.02 | 0.236 | 0.387 |
| ADHD | 0.074 | 1.03E-05 | 1.48E-04 |
| ASD | 0.019 | 0.270 | 0.425 |
| MDD | 0.039 | 0.027 | 0.074 |
| BIP | 0.053 | 0.003 | 0.012 |
| SCZ | 0.048 | 0.020 | 0.061 |
| CROSS | 0.047 | 0.006 | 0.025 |
| COMP | -0.033 | 0.050 | 0.116 |
| MP | 0.074 | 2.15E-04 | 0.002 |
| NDV | 0.079 | 3.54E-06 | 8.50E-05 |
| **CBCL Internal** | | | |
| AN | 0.023 | 0.197 | 0.338 |
| OCD | 0.01 | 0.559 | 0.700 |
| TS | 0.019 | 0.275 | 0.426 |

| | | | |
|---|---|---|---|
| ADHD | 0.033 | 0.052 | 0.118 |
| ASD | 0.044 | 0.011 | 0.038 |
| MDD | 0.076 | 1.39E-05 | 1.82E-04 |
| BIP | 0.009 | 0.634 | 0.730 |
| SCZ | 0.006 | 0.775 | 0.845 |
| CROSS | 0.019 | 0.271 | 0.425 |
| COMP | 0.007 | 0.683 | 0.762 |
| MP | 0.025 | 0.208 | 0.349 |
| NDV | 0.065 | 1.63E-04 | 0.001 |
| **CBCL Attention** | | | |
| AN | -0.042 | 0.024 | 0.068 |
| OCD | -0.004 | 0.828 | 0.883 |
| TS | 0.015 | 0.411 | 0.564 |
| ADHD | 0.096 | 4.34E-08 | 1.56E-06 |
| ASD | 0.05 | 0.005 | 0.021 |
| MDD | 0.063 | 0.001 | 0.003 |
| BIP | 0.018 | 0.329 | 0.480 |
| SCZ | -0.005 | 0.825 | 0.883 |
| CROSS | 0.057 | 0.001 | 0.008 |
| COMP | -0.036 | 0.038 | 0.092 |
| MP | 0.029 | 0.172 | 0.298 |
| NDV | 0.097 | 3.34E-08 | 1.56E-06 |
| **CBCL Rulebreak** | | | |
| AN | -0.036 | 0.033 | 0.083 |
| OCD | -0.018 | 0.256 | 0.410 |
| TS | 0.008 | 0.610 | 0.724 |
| ADHD | 0.072 | 7.27E-06 | 1.31E-04 |
| ASD | -0.002 | 0.890 | 0.929 |
| MDD | 0.04 | 0.016 | 0.054 |
| BIP | 0.033 | 0.049 | 0.115 |
| SCZ | 0.05 | 0.010 | 0.035 |
| CROSS | 0.06 | 2.20E-04 | 0.002 |
| COMP | -0.029 | 0.064 | 0.136 |
| MP | 0.075 | 7.91E-05 | 0.001 |
| NDV | 0.072 | 7.15E-06 | 1.31E-04 |
| **CBCL Aggressive** | | | |
| AN | -0.013 | 0.451 | 0.596 |
| OCD | -0.008 | 0.630 | 0.730 |
| TS | 0.026 | 0.121 | 0.229 |
| ADHD | 0.065 | 1.08E-04 | 0.001 |
| ASD | 0.011 | 0.522 | 0.678 |
| MDD | 0.027 | 0.118 | 0.226 |
| BIP | 0.05 | 0.005 | 0.021 |
| SCZ | 0.048 | 0.018 | 0.056 |
| CROSS | 0.065 | 1.42E-04 | 0.001 |

| | | | |
|---|---|---|---|
| COMP | -0.018 | 0.281 | 0.430 |
| MP | 0.056 | 0.005 | 0.021 |
| NDV | 0.062 | 2.45E-04 | 0.002 |
| **CBCL Anx/Dep** | | | |
| AN | 0.009 | 0.646 | 0.738 |
| OCD | 0.027 | 0.135 | 0.249 |
| TS | 0.018 | 0.330 | 0.480 |
| ADHD | 0.026 | 0.145 | 0.264 |
| ASD | 0.014 | 0.442 | 0.594 |
| MDD | 0.066 | 4.02E-04 | 0.003 |
| BIP | -0.009 | 0.621 | 0.727 |
| SCZ | 0.006 | 0.783 | 0.847 |
| CROSS | 0.01 | 0.594 | 0.719 |
| COMP | 0.023 | 0.205 | 0.347 |
| MP | 0.011 | 0.613 | 0.724 |
| NDV | 0.049 | 0.007 | 0.026 |
| **CBCL With/Dep** | | | |
| AN | -0.008 | 0.669 | 0.759 |
| OCD | 0.013 | 0.447 | 0.596 |
| TS | 0.009 | 0.603 | 0.724 |
| ADHD | 0.032 | 0.064 | 0.136 |
| ASD | 0.045 | 0.009 | 0.033 |
| MDD | 0.051 | 0.004 | 0.016 |
| BIP | -0.01 | 0.583 | 0.712 |
| SCZ | 0 | 0.984 | 0.984 |
| CROSS | 0.018 | 0.293 | 0.439 |
| COMP | -0.002 | 0.903 | 0.936 |
| MP | 0.011 | 0.574 | 0.712 |
| NDV | 0.065 | 1.49E-04 | 0.001 |
| **CBCL Somatic** | | | |
| AN | 0.01 | 0.584 | 0.712 |
| OCD | -0.004 | 0.838 | 0.884 |
| TS | 0.037 | 0.030 | 0.080 |
| ADHD | 0.04 | 0.018 | 0.058 |
| ASD | 0.032 | 0.064 | 0.136 |
| MDD | 0.046 | 0.009 | 0.033 |
| BIP | -0.007 | 0.715 | 0.792 |
| SCZ | -0.004 | 0.841 | 0.884 |
| CROSS | 0.033 | 0.062 | 0.136 |
| COMP | -0.011 | 0.535 | 0.681 |
| MP | 0.002 | 0.922 | 0.948 |
| NDV | 0.037 | 0.030 | 0.080 |
| **CBCL Social** | | | |
| AN | -0.026 | 0.150 | 0.266 |
| OCD | -0.015 | 0.388 | 0.554 |

| | | | |
|---|---|---|---|
| TS | 0.007 | 0.677 | 0.761 |
| ADHD | 0.057 | 0.001 | 0.004 |
| ASD | 0.031 | 0.075 | 0.154 |
| MDD | 0.055 | 0.002 | 0.008 |
| BIP | 0.027 | 0.134 | 0.249 |
| SCZ | 0.012 | 0.547 | 0.691 |
| CROSS | 0.036 | 0.035 | 0.087 |
| COMP | -0.03 | 0.074 | 0.154 |
| MP | 0.039 | 0.051 | 0.117 |
| NDV | 0.083 | 1.10E-06 | 3.18E-05 |
| **CBCL Thought** | | | |
| AN | -0.03 | 0.108 | 0.211 |
| OCD | 0.015 | 0.400 | 0.555 |
| TS | 0.038 | 0.033 | 0.083 |
| ADHD | 0.04 | 0.023 | 0.066 |
| ASD | 0.025 | 0.160 | 0.281 |
| MDD | 0.042 | 0.022 | 0.065 |
| BIP | 0.016 | 0.401 | 0.555 |
| SCZ | -0.022 | 0.311 | 0.462 |
| CROSS | 0.041 | 0.023 | 0.066 |
| COMP | -0.006 | 0.729 | 0.801 |
| MP | 0.013 | 0.531 | 0.681 |
| NDV | 0.052 | 0.004 | 0.016 |
| **Psychosis Spectrum** | | | |
| AN | 0.009 | 0.519 | 0.678 |
| OCD | 0 | 0.979 | 0.984 |
| TS | 0.021 | 0.101 | 0.199 |
| ADHD | 0.052 | 3.83E-05 | 4.24E-04 |
| ASD | 0.03 | 0.020 | 0.061 |
| MDD | 0.059 | 8.24E-06 | 1.32E-04 |
| BIP | 0.016 | 0.240 | 0.389 |
| SCZ | 0.026 | 0.095 | 0.190 |
| CROSS | 0.015 | 0.236 | 0.387 |
| COMP | -0.001 | 0.936 | 0.956 |
| MP | 0.049 | 0.001 | 0.006 |
| NDV | 0.072 | 2.36E-08 | 1.56E-06 |

## SUPPLEMENTAL TABLE 4. Results from R-squared change models in ABCD

Model 1: covariates only
Model 2: covariates + PGS of interest
Model 3: covariates + PGS of interest + NDV PGS

**Included covariates**
Fixed effects: age, sex, top 5 principal components
Random effects: site

| | R2 (*Model 1*) | R2 (*Model 2*) | P (*Model 2*) | R2 (*Model 3*) | P (*Model 3*) |
|---|---|---|---|---|---|
| **Baseline CBCL Total** | | | | | |
| AN | 0.030 | 0.030 | 0.819 | 0.038 | 2.67E-10 |
| OCD | 0.030 | 0.030 | 0.238 | 0.038 | 3.47E-10 |
| TS | 0.030 | 0.030 | 0.021 | 0.038 | 1.09E-09 |
| ADHD | 0.030 | 0.036 | 4.07E-08 | 0.038 | 4.78E-04 |
| ASD | 0.030 | 0.031 | 0.009 | 0.038 | 9.29E-09 |
| MDD | 0.030 | 0.034 | 2.07E-06 | 0.038 | 2.39E-05 |
| BIP | 0.030 | 0.030 | 0.051 | 0.038 | 8.71E-10 |
| SCZ | 0.030 | 0.030 | 0.075 | 0.038 | 2.20E-10 |
| CROSS | 0.030 | 0.032 | 0.000 | 0.038 | 7.80E-08 |
| COMPULS | 0.030 | 0.030 | 0.459 | 0.038 | 4.04E-10 |
| MOODPSYCH | 0.030 | 0.031 | 0.008 | 0.038 | 6.21E-09 |
| | | | | | |
| **Baseline CBCL External** | | | | | |
| AN | 0.018 | 0.018 | 0.947 | 0.026 | 2.35E-09 |
| OCD | 0.018 | 0.019 | 0.280 | 0.026 | 3.71E-09 |
| TS | 0.018 | 0.018 | 0.252 | 0.025 | 5.65E-09 |
| ADHD | 0.018 | 0.026 | 6.84E-09 | 0.027 | 0.007 |
| ASD | 0.018 | 0.019 | 0.071 | 0.026 | 9.22E-09 |
| MDD | 0.018 | 0.021 | 0.000 | 0.025 | 2.88E-06 |
| BIP | 0.018 | 0.020 | 0.008 | 0.026 | 1.34E-08 |
| SCZ | 0.018 | 0.018 | 0.604 | 0.026 | 3.05E-09 |
| CROSS | 0.018 | 0.021 | 0.000 | 0.026 | 4.89E-07 |
| COMPULS | 0.018 | 0.018 | 0.849 | 0.025 | 3.28E-09 |
| MOODPSYCH | 0.018 | 0.020 | 0.001 | 0.026 | 1.31E-07 |
| | | | | | |
| **Baseline CBCL Internal** | | | | | |
| AN | 0.025 | 0.026 | 0.022 | 0.028 | 0.001 |
| OCD | 0.025 | 0.026 | 0.109 | 0.028 | 4.34E-04 |
| TS | 0.025 | 0.025 | 0.019 | 0.028 | 8.76E-04 |
| ADHD | 0.025 | 0.026 | 0.041 | 0.027 | 0.003 |
| ASD | 0.025 | 0.025 | 0.122 | 0.027 | 0.001 |
| MDD | 0.025 | 0.028 | 0.000 | 0.029 | 0.230 |

| | | | | | |
|---|---|---|---|---|---|
| BIP | 0.025 | 0.025 | 0.138 | 0.027 | 6.43E-04 |
| SCZ | 0.025 | 0.025 | 0.248 | 0.028 | 3.50E-04 |
| CROSS | 0.025 | 0.027 | 0.001 | 0.028 | 0.008 |
| COMPULS | 0.025 | 0.026 | 0.057 | 0.028 | 6.86E-04 |
| MOODPSYCH | 0.025 | 0.026 | 0.016 | 0.028 | 0.002 |
| | | | | | |
| **Baseline PQ-BC** | | | | | |
| AN | 0.041 | 0.041 | 0.442 | 0.048 | 1.02E-08 |
| OCD | 0.041 | 0.041 | 0.677 | 0.047 | 2.55E-08 |
| TS | 0.041 | 0.042 | 0.019 | 0.048 | 7.89E-08 |
| ADHD | 0.041 | 0.043 | 0.001 | 0.047 | 9.42E-06 |
| ASD | 0.041 | 0.041 | 0.171 | 0.048 | 2.15E-08 |
| MDD | 0.041 | 0.045 | 7.85E-06 | 0.047 | 4.25E-04 |
| BIP | 0.041 | 0.041 | 0.190 | 0.048 | 9.73E-09 |
| SCZ | 0.041 | 0.041 | 0.479 | 0.047 | 2.17E-08 |
| CROSS | 0.041 | 0.042 | 0.003 | 0.047 | 1.03E-06 |
| COMPULS | 0.041 | 0.041 | 0.285 | 0.047 | 3.69E-08 |
| MOODPSYCH | 0.041 | 0.041 | 0.181 | 0.047 | 6.24E-08 |
| | | | | | |
| **Year 2 CBCL Total** | | | | | |
| AN | 0.028 | 0.028 | 0.395 | 0.035 | 1.37E-08 |
| OCD | 0.028 | 0.028 | 0.944 | 0.035 | 3.15E-08 |
| TS | 0.028 | 0.028 | 0.086 | 0.035 | 6.97E-08 |
| ADHD | 0.028 | 0.032 | 1.63E-05 | 0.035 | 4.07E-04 |
| ASD | 0.028 | 0.030 | 0.003 | 0.035 | 2.74E-06 |
| MDD | 0.028 | 0.031 | 0.000 | 0.035 | 1.38E-04 |
| BIP | 0.028 | 0.028 | 0.042 | 0.035 | 8.92E-08 |
| SCZ | 0.028 | 0.028 | 0.152 | 0.035 | 2.24E-08 |
| CROSS | 0.028 | 0.030 | 0.002 | 0.035 | 1.87E-06 |
| COMPULS | 0.028 | 0.028 | 0.348 | 0.035 | 1.76E-08 |
| MOODPSYCH | 0.028 | 0.029 | 0.008 | 0.035 | 5.49E-07 |
| | | | | | |
| **Year 2 CBCL External** | | | | | |
| AN | 0.016 | 0.016 | 0.150 | 0.022 | 1.25E-06 |
| OCD | 0.016 | 0.016 | 0.284 | 0.021 | 3.09E-06 |
| TS | 0.016 | 0.016 | 0.236 | 0.021 | 5.51E-06 |
| ADHD | 0.016 | 0.020 | 1.03E-05 | 0.021 | 0.026 |
| ASD | 0.016 | 0.016 | 0.270 | 0.021 | 2.85E-06 |
| MDD | 0.016 | 0.016 | 0.027 | 0.021 | 2.71E-05 |
| BIP | 0.016 | 0.018 | 0.003 | 0.022 | 1.37E-05 |
| SCZ | 0.016 | 0.016 | 0.255 | 0.021 | 2.80E-06 |
| CROSS | 0.016 | 0.018 | 0.006 | 0.021 | 7.53E-05 |
| COMPULS | 0.016 | 0.017 | 0.050 | 0.022 | 1.38E-06 |
| MOODPSYCH | 0.016 | 0.019 | 0.000 | 0.022 | 1.47E-04 |
| | | | | | |

| | | | | | |
|---|---|---|---|---|---|
| **Year 2 CBCL Internal** | | | | | |
| AN | 0.021 | 0.022 | 0.197 | 0.025 | 2.71E-04 |
| OCD | 0.021 | 0.021 | 0.559 | 0.024 | 1.72E-04 |
| TS | 0.021 | 0.021 | 0.275 | 0.024 | 2.30E-04 |
| ADHD | 0.021 | 0.022 | 0.052 | 0.025 | 0.001 |
| ASD | 0.021 | 0.023 | 0.011 | 0.025 | 0.004 |
| MDD | 0.021 | 0.026 | 0.000 | 0.026 | 0.205 |
| BIP | 0.021 | 0.021 | 0.634 | 0.024 | 1.84E-04 |
| SCZ | 0.021 | 0.022 | 0.237 | 0.025 | 1.33E-04 |
| CROSS | 0.021 | 0.022 | 0.271 | 0.024 | 3.08E-04 |
| COMPULS | 0.021 | 0.021 | 0.683 | 0.024 | 1.78E-04 |
| MOODPSYCH | 0.021 | 0.022 | 0.208 | 0.024 | 3.66E-04 |
| | | | | | |
| **Year 2 PQ-BC** | | | | | |
| AN | 0.012 | 0.013 | 0.519 | 0.020 | 2.93E-08 |
| OCD | 0.012 | 0.012 | 0.979 | 0.020 | 2.37E-08 |
| TS | 0.012 | 0.013 | 0.101 | 0.020 | 5.30E-08 |
| ADHD | 0.012 | 0.016 | 3.83E-05 | 0.020 | 1.51E-04 |
| ASD | 0.012 | 0.014 | 0.020 | 0.020 | 3.58E-07 |
| MDD | 0.012 | 0.017 | 8.24E-06 | 0.020 | 3.98E-04 |
| BIP | 0.012 | 0.013 | 0.240 | 0.020 | 3.87E-08 |
| SCZ | 0.012 | 0.013 | 0.404 | 0.020 | 2.04E-08 |
| CROSS | 0.012 | 0.013 | 0.236 | 0.020 | 3.89E-08 |
| COMPULS | 0.012 | 0.012 | 0.936 | 0.020 | 2.02E-08 |
| MOODPSYCH | 0.012 | 0.015 | 0.001 | 0.021 | 8.29E-07 |

**SUPPLEMENTAL TABLE 5. Effect sizes, and p- and q-values from CBCL-PGS mixed models at baseline (ABCD, age 9-10, using PRSCS method)**

p-values in red reflect significance after FDR correction

This sheet reports results from PGS-psychopathology analyses using PGS generated via PRS-CS

All statistics shown are derived from two-sided linear mixed effects regressions

**N = 4459**

**Included covariates**

Fixed effects: age, sex, top 5 principal components

Random effects: site

FDR corrects for 12 scales x 12 PGS = 144 comparisons

| | *Beta* | *p (uncorrected)* | *p (FDR)* |
|---|---|---|---|
| **CBCL Total** | | | |
| AN | -0.001 | 0.947 | 0.947 |
| OCD | 0.022 | 0.121 | 0.192 |
| TS | 0.027 | 0.066 | 0.119 |
| ADHD | 0.104 | 1.94E-13 | 5.59E-12 |
| ASD | 0.053 | 2.28E-04 | 0.001 |
| MDD | 0.074 | 7.00E-07 | 5.60E-06 |
| BIP | 0.013 | 0.381 | 0.485 |
| SCZ | 0.03 | 0.069 | 0.122 |
| CROSS | 0.069 | 1.81E-05 | 0.000 |
| COMP | 0.025 | 0.081 | 0.137 |
| MP | 0.035 | 0.028 | 0.061 |
| NDV | 0.119 | 4.44E-16 | 6.39E-14 |
| **CBCL External** | | | |
| AN | 0.009 | 0.581 | 0.675 |
| OCD | 0.021 | 0.153 | 0.233 |
| TS | 0.006 | 0.673 | 0.763 |
| ADHD | 0.113 | 4.44E-15 | 3.20E-13 |
| ASD | 0.046 | 0.002 | 0.006 |
| MDD | 0.068 | 6.84E-06 | 4.92E-05 |
| BIP | 0.025 | 0.098 | 0.163 |
| SCZ | 0.038 | 0.025 | 0.054 |
| CROSS | 0.066 | 5.56E-05 | 2.86E-04 |
| COMP | 0.017 | 0.242 | 0.338 |
| MP | 0.046 | 0.005 | 0.015 |

| | | | |
|---|---|---|---|
| NDV | 0.116 | 8.88E-15 | 4.26E-13 |
| **CBCL Internal** | | | |
| AN | -0.041 | 0.010 | 0.026 |
| OCD | 0.025 | 0.094 | 0.160 |
| TS | 0.032 | 0.035 | 0.069 |
| ADHD | 0.049 | 0.001 | 0.004 |
| ASD | 0.043 | 0.004 | 0.014 |
| MDD | 0.063 | 4.87E-05 | 2.60E-04 |
| BIP | 0.018 | 0.241 | 0.334 |
| SCZ | 0.031 | 0.066 | 0.119 |
| CROSS | 0.069 | 3.20E-05 | 1.84E-04 |
| COMP | 0.041 | 0.005 | 0.015 |
| MP | 0.037 | 0.025 | 0.056 |
| NDV | 0.078 | 3.50E-07 | 3.36E-06 |
| **CBCL Attention** | | | |
| AN | 0.013 | 0.403 | 0.504 |
| OCD | 0.005 | 0.713 | 0.786 |
| TS | 0.025 | 0.089 | 0.152 |
| ADHD | 0.108 | 1.23E-13 | 4.48E-12 |
| ASD | 0.042 | 0.005 | 0.016 |
| MDD | 0.04 | 0.008 | 0.023 |
| BIP | -0.003 | 0.861 | 0.879 |
| SCZ | 0.014 | 0.394 | 0.497 |
| CROSS | 0.052 | 0.002 | 0.006 |
| COMP | 0.004 | 0.785 | 0.850 |
| MP | 0.011 | 0.500 | 0.590 |
| NDV | 0.098 | 6.57E-11 | 1.18E-09 |
| **CBCL Rulebreak** | | | |
| AN | 0.028 | 0.059 | 0.110 |
| OCD | 0.005 | 0.715 | 0.786 |
| TS | 0.008 | 0.589 | 0.678 |
| ADHD | 0.094 | 4.66E-12 | 1.12E-10 |
| ASD | 0.029 | 0.038 | 0.074 |
| MDD | 0.059 | 4.12E-05 | 2.28E-04 |
| BIP | 0.021 | 0.132 | 0.207 |
| SCZ | 0.038 | 0.017 | 0.041 |
| CROSS | 0.048 | 0.002 | 0.006 |
| COMP | -0.003 | 0.844 | 0.874 |
| MP | 0.045 | 0.003 | 0.010 |
| NDV | 0.095 | 1.41E-11 | 2.89E-10 |
| **CBCL Aggressive** | | | |
| AN | 0.002 | 0.874 | 0.887 |
| OCD | 0.021 | 0.142 | 0.219 |
| TS | 0.015 | 0.300 | 0.409 |
| ADHD | 0.09 | 3.02E-10 | 5.44E-09 |

| | | | |
|---|---|---|---|
| ASD | 0.039 | 0.007 | 0.021 |
| MDD | 0.046 | 0.002 | 0.008 |
| BIP | 0.011 | 0.442 | 0.544 |
| SCZ | 0.016 | 0.345 | 0.443 |
| CROSS | 0.049 | 0.003 | 0.009 |
| COMP | 0.019 | 0.180 | 0.270 |
| MP | 0.021 | 0.199 | 0.292 |
| NDV | 0.089 | 1.93E-09 | 2.78E-08 |
| **CBCL Anx/Dep** | | | |
| AN | -0.036 | 0.031 | 0.063 |
| OCD | 0.036 | 0.021 | 0.049 |
| TS | 0.041 | 0.009 | 0.024 |
| ADHD | 0.035 | 0.024 | 0.054 |
| ASD | 0.036 | 0.023 | 0.053 |
| MDD | 0.038 | 0.019 | 0.046 |
| BIP | 0.02 | 0.218 | 0.311 |
| SCZ | 0.035 | 0.051 | 0.097 |
| CROSS | 0.077 | 9.51E-06 | 6.52E-05 |
| COMP | 0.052 | 8.16E-04 | 0.003 |
| MP | 0.036 | 0.039 | 0.075 |
| NDV | 0.056 | 4.56E-04 | 0.00199 |
| **CBCL With/Dep** | | | |
| AN | -0.003 | 0.835 | 0.865 |
| OCD | 0.027 | 0.058 | 0.110 |
| TS | 0.004 | 0.799 | 0.844 |
| ADHD | 0.024 | 0.100 | 0.166 |
| ASD | 0.039 | 0.008 | 0.023 |
| MDD | 0.052 | 5.84E-04 | 0.002 |
| BIP | 0.004 | 0.809 | 0.844 |
| SCZ | 0.01 | 0.563 | 0.660 |
| CROSS | 0.037 | 0.024 | 0.054 |
| COMP | 0.025 | 0.078 | 0.134 |
| MP | 0.013 | 0.417 | 0.517 |
| NDV | 0.053 | 4.09E-04 | 0.00184 |
| **CBCL Somatic** | | | |
| AN | -0.015 | 0.336 | 0.440 |
| OCD | 0.004 | 0.804 | 0.844 |
| TS | 0.027 | 0.072 | 0.126 |
| ADHD | 0.048 | 0.001 | 0.005 |
| ASD | 0.033 | 0.027 | 0.058 |
| MDD | 0.062 | 6.77E-05 | 3.25E-04 |
| BIP | 0.002 | 0.917 | 0.924 |
| SCZ | 0.013 | 0.440 | 0.544 |
| CROSS | 0.045 | 0.006 | 0.019 |
| COMP | 0.013 | 0.367 | 0.476 |

| | | | |
|---|---|---|---|
| MP | 0.015 | 0.363 | 0.475 |
| NDV | 0.075 | 1.02E-06 | 8.16E-06 |
| **CBCL Social** | | | |
| AN | 0.015 | 0.333 | 0.439 |
| OCD | 0.017 | 0.232 | 0.324 |
| TS | 0.021 | 0.157 | 0.236 |
| ADHD | 0.071 | 5.29E-07 | 4.48E-06 |
| ASD | 0.03 | 0.037 | 0.073 |
| MDD | 0.065 | 1.41E-05 | 8.80E-05 |
| BIP | -0.005 | 0.714 | 0.786 |
| SCZ | -0.008 | 0.630 | 0.720 |
| CROSS | 0.035 | 0.028 | 0.059 |
| COMP | 0.017 | 0.228 | 0.329 |
| MP | -0.003 | 0.858 | 0.879 |
| NDV | 0.084 | 1.21E-08 | 1.45E-07 |
| **CBCL Thought** | | | |
| AN | 0.004 | 0.798 | 0.844 |
| OCD | 0.028 | 0.066 | 0.119 |
| TS | 0.033 | 0.033 | 0.067 |
| ADHD | 0.085 | 1.77E-08 | 2.32E-07 |
| ASD | 0.048 | 0.002 | 0.007 |
| MDD | 0.034 | 0.030 | 0.062 |
| BIP | 0.015 | 0.339 | 0.440 |
| SCZ | 0.006 | 0.711 | 0.786 |
| CROSS | 0.068 | 6.30E-05 | 3.13E-04 |
| COMP | 0.031 | 0.039 | 0.075 |
| MP | 0.013 | 0.440 | 0.535 |
| NDV | 0.081 | 2.49E-07 | 2.56E-06 |
| **Psychosis Spectrum** | | | |
| AN | 0.004 | 0.766 | 0.829 |
| OCD | 0.016 | 0.196 | 0.282 |
| TS | 0.021 | 0.105 | 0.172 |
| ADHD | 0.06 | 2.13E-06 | 1.62E-05 |
| ASD | 0.033 | 0.011 | 0.028 |
| MDD | 0.058 | 1.32E-05 | 8.65E-05 |
| BIP | -0.009 | 0.498 | 0.598 |
| SCZ | 0.01 | 0.496 | 0.598 |
| CROSS | 0.04 | 0.005 | 0.015 |
| COMP | 0.014 | 0.253 | 0.350 |
| MP | 0.013 | 0.370 | 0.476 |
| NDV | 0.078 | 3.01E-09 | 3.94E-08 |

## SUPPLEMENTAL TABLE 6. Effect sizes, and p- and q-values from PGS-psychopathology relationships (Generation R, age 9)

p-values in red reflect significance after FDR correction

All statistics shown are derived from two-sided linear mixed effects regressions

**N = 1850**

**Included covariates**

Fixed effects: age, sex, top 5 principal components

FDR corrects for 12 scales x 3 PGS = 36 comparisons

| | *Beta* | *p (uncorrected)* | *p (FDR)* |
|---|---|---|---|
| **CBCL Total** | | | |
| AN | -0.021 | 0.367 | 0.660 |
| OCD | 0.006 | 0.787 | 0.895 |
| TS | 0.040 | 0.087 | 0.296 |
| ADHD | 0.096 | 3.60E-05 | 0.001 |
| ASD | 0.037 | 0.116 | 0.334 |
| MDD | 0.056 | 0.016 | 0.097 |
| BIP | -0.024 | 0.312 | 0.607 |
| SCZ | 0.063 | 0.008 | 0.066 |
| CROSS | 0.020 | 0.399 | 0.685 |
| COMP | 0.012 | 0.609 | 0.811 |
| MP | 0.049 | 0.041 | 0.197 |
| NDV | 0.106 | 5.80E-06 | 2.78E-04 |
| **CBCL External** | | | |
| AN | -0.018 | 0.433 | 0.704 |
| OCD | 0.006 | 0.809 | 0.901 |
| TS | 0.037 | 0.112 | 0.334 |
| ADHD | 0.070 | 0.003 | 0.032 |
| ASD | 0.042 | 0.071 | 0.269 |
| MDD | 0.040 | 0.090 | 0.296 |
| BIP | -0.009 | 0.698 | 0.872 |
| SCZ | 0.055 | 0.021 | 0.117 |
| CROSS | 0.018 | 0.438 | 0.704 |
| COMP | 0.008 | 0.726 | 0.874 |
| MP | 0.049 | 0.041 | 0.197 |
| NDV | 0.090 | 1.00E-04 | 0.002 |
| **CBCL Internal** | | | |
| AN | 0.001 | 0.965 | 0.974 |
| OCD | 0.010 | 0.666 | 0.860 |

| | | | |
|---|---|---|---|
| TS | 0.013 | 0.568 | 0.795 |
| ADHD | 0.037 | 0.112 | 0.334 |
| ASD | 0.018 | 0.432 | 0.704 |
| MDD | 0.042 | 0.075 | 0.275 |
| BIP | -0.029 | 0.223 | 0.493 |
| SCZ | 0.039 | 0.101 | 0.315 |
| CROSS | 0.011 | 0.639 | 0.837 |
| COMP | 0.010 | 0.673 | 0.860 |
| MP | 0.024 | 0.320 | 0.607 |
| NDV | 0.046 | 0.051 | 0.219 |
| **CBCL Attention** | | | |
| AN | -0.058 | 0.012 | 0.078 |
| OCD | -0.001 | 0.962 | 0.974 |
| TS | 0.032 | 0.165 | 0.395 |
| ADHD | 0.118 | 3.00E-07 | 3.74E-05 |
| ASD | 0.034 | 0.148 | 0.380 |
| MDD | 0.042 | 0.070 | 0.269 |
| BIP | -0.014 | 0.557 | 0.795 |
| SCZ | 0.060 | 0.012 | 0.078 |
| CROSS | 0.015 | 0.532 | 0.773 |
| COMP | -0.005 | 0.828 | 0.902 |
| MP | 0.043 | 0.067 | 0.269 |
| NDV | 0.116 | 5.20E-07 | 3.74E-05 |
| **CBCL Rulebreak** | | | |
| AN | -0.027 | 0.244 | 0.525 |
| OCD | 0.021 | 0.371 | 0.660 |
| TS | 0.039 | 0.089 | 0.296 |
| ADHD | 0.061 | 0.009 | 0.069 |
| ASD | 0.006 | 0.795 | 0.895 |
| MDD | 0.040 | 0.085 | 0.296 |
| BIP | -0.019 | 0.419 | 0.703 |
| SCZ | 0.019 | 0.420 | 0.703 |
| CROSS | 0.009 | 0.712 | 0.874 |
| COMP | 0.015 | 0.527 | 0.773 |
| MP | 0.017 | 0.480 | 0.735 |
| NDV | 0.067 | 0.004 | 0.042 |
| **CBCL Aggressive** | | | |
| AN | -0.013 | 0.576 | 0.796 |
| OCD | 0.000 | 0.988 | 0.988 |
| TS | 0.032 | 0.160 | 0.391 |
| ADHD | 0.067 | 0.004 | 0.042 |
| ASD | 0.052 | 0.027 | 0.136 |
| MDD | 0.036 | 0.130 | 0.347 |
| BIP | -0.005 | 0.839 | 0.902 |
| SCZ | 0.063 | 0.008 | 0.066 |

| | | | |
|---|---|---|---|
| CROSS | 0.020 | 0.391 | 0.679 |
| COMP | 0.005 | 0.827 | 0.902 |
| MP | 0.056 | 0.019 | 0.111 |
| NDV | 0.091 | 1.00E-04 | 0.002 |
| **CBCL Anx/Dep** | | | |
| AN | 0.023 | 0.320 | 0.607 |
| OCD | 0.022 | 0.358 | 0.657 |
| TS | 0.026 | 0.257 | 0.537 |
| ADHD | 0.036 | 0.128 | 0.347 |
| ASD | 0.023 | 0.320 | 0.607 |
| MDD | 0.039 | 0.097 | 0.310 |
| BIP | -0.024 | 0.304 | 0.607 |
| SCZ | 0.034 | 0.154 | 0.389 |
| CROSS | 0.013 | 0.593 | 0.806 |
| COMP | 0.031 | 0.179 | 0.413 |
| MP | 0.022 | 0.361 | 0.657 |
| NDV | 0.046 | 0.049 | 0.219 |
| **CBCL With/Dep** | | | |
| AN | -0.010 | 0.678 | 0.860 |
| OCD | -0.015 | 0.515 | 0.773 |
| TS | 0.018 | 0.442 | 0.704 |
| ADHD | -0.008 | 0.740 | 0.874 |
| ASD | 0.014 | 0.558 | 0.795 |
| MDD | 0.008 | 0.735 | 0.874 |
| BIP | -0.032 | 0.174 | 0.410 |
| SCZ | 0.012 | 0.618 | 0.817 |
| CROSS | -0.017 | 0.463 | 0.725 |
| COMP | -0.008 | 0.734 | 0.874 |
| MP | -0.006 | 0.789 | 0.895 |
| NDV | 0.008 | 0.722 | 0.874 |
| **CBCL Somatic** | | | |
| AN | -0.023 | 0.319 | 0.607 |
| OCD | 0.007 | 0.757 | 0.881 |
| TS | -0.020 | 0.391 | 0.679 |
| ADHD | 0.053 | 0.024 | 0.125 |
| ASD | 0.003 | 0.913 | 0.945 |
| MDD | 0.046 | 0.051 | 0.219 |
| BIP | -0.012 | 0.608 | 0.811 |
| SCZ | 0.044 | 0.069 | 0.269 |
| CROSS | 0.025 | 0.287 | 0.591 |
| COMP | -0.015 | 0.531 | 0.773 |
| MP | 0.037 | 0.124 | 0.347 |
| NDV | 0.046 | 0.052 | 0.219 |
| **CBCL Social** | | | |
| AN | -0.001 | 0.967 | 0.974 |

| | | | |
|---|---|---|---|
| OCD | -0.010 | 0.681 | 0.860 |
| TS | 0.058 | 0.012 | 0.078 |
| ADHD | 0.088 | 2.00E-04 | 0.004 |
| ASD | 0.027 | 0.248 | 0.525 |
| MDD | 0.057 | 0.015 | 0.095 |
| BIP | -0.029 | 0.211 | 0.474 |
| SCZ | 0.042 | 0.078 | 0.279 |
| CROSS | 0.007 | 0.758 | 0.881 |
| COMP | 0.009 | 0.702 | 0.872 |
| MP | 0.028 | 0.239 | 0.521 |
| NDV | 0.096 | 4.30E-05 | 0.001 |
| **CBCL Thought** | | | |
| AN | 0.003 | 0.895 | 0.934 |
| OCD | 0.013 | 0.569 | 0.795 |
| TS | 0.031 | 0.181 | 0.413 |
| ADHD | 0.083 | 4.00E-04 | 0.006 |
| ASD | 0.018 | 0.445 | 0.704 |
| MDD | 0.062 | 0.008 | 0.066 |
| BIP | -0.013 | 0.580 | 0.796 |
| SCZ | 0.078 | 0.001 | 0.014 |
| CROSS | 0.036 | 0.128 | 0.347 |
| COMP | 0.022 | 0.344 | 0.643 |
| MP | 0.064 | 0.007 | 0.066 |
| NDV | 0.084 | 3.00E-04 | 0.005 |
| **Psychosis spectrum** | | | |
| AN | 0.011 | 0.833 | 0.902 |
| OCD | -0.014 | 0.793 | 0.895 |
| TS | 0.083 | 0.115 | 0.334 |
| ADHD | 0.013 | 0.814 | 0.901 |
| ASD | 0.009 | 0.872 | 0.917 |
| MDD | 0.010 | 0.858 | 0.908 |
| BIP | -0.080 | 0.141 | 0.368 |
| SCZ | 0.003 | 0.962 | 0.974 |
| CROSS | -0.035 | 0.523 | 0.773 |
| COMP | -0.010 | 0.852 | 0.908 |
| MP | -0.039 | 0.468 | 0.725 |
| NDV | 0.076 | 0.158 | 0.391 |

**SUPPLEMENTAL TABLE 7. Effect sizes, and p- and q-values of PGS-psychopathology relationships (Generation R, age 13)**

p-values in red reflect significance after FDR correction

All statistics shown are derived from two-sided linear mixed effects regressions

**N = 1791**

**Included covariates**

Fixed effects: age, sex, top 5 principal components

FDR corrects for 12 scales x 3 PGS = 36 comparisons

| | *Beta* | *p (uncorrected)* | *p (FDR)* |
|---|---|---|---|
| **CBCL Total** | | | |
| AN | 0.011 | 0.628 | 0.786 |
| OCD | 0.000 | 0.999 | 0.999 |
| TS | 0.035 | 0.134 | 0.302 |
| ADHD | 0.110 | 3.30E-06 | 2.38E-04 |
| ASD | 0.037 | 0.121 | 0.302 |
| MDD | 0.036 | 0.134 | 0.302 |
| BIP | 0.012 | 0.599 | 0.764 |
| SCZ | 0.053 | 0.030 | 0.127 |
| CROSS | 0.047 | 0.050 | 0.174 |
| COMP | 0.027 | 0.251 | 0.441 |
| MP | 0.058 | 0.015 | 0.085 |
| NDV | 0.107 | 5.40E-06 | 2.59E-04 |
| **CBCL External** | | | |
| AN | 0.020 | 0.398 | 0.590 |
| OCD | 0.007 | 0.786 | 0.902 |
| TS | 0.033 | 0.158 | 0.335 |
| ADHD | 0.096 | 5.00E-05 | 9.00E-04 |
| ASD | 0.029 | 0.219 | 0.407 |
| MDD | 0.020 | 0.393 | 0.590 |
| BIP | 0.032 | 0.177 | 0.362 |
| SCZ | 0.047 | 0.052 | 0.179 |
| CROSS | 0.058 | 0.015 | 0.085 |
| COMP | 0.034 | 0.155 | 0.333 |
| MP | 0.065 | 0.007 | 0.045 |
| NDV | 0.098 | 3.10E-05 | 0.001 |
| **CBCL Internal** | | | |
| AN | 0.039 | 0.098 | 0.280 |
| OCD | 0.007 | 0.761 | 0.890 |
| TS | 0.028 | 0.231 | 0.419 |
| ADHD | 0.067 | 0.005 | 0.037 |

| | | | |
|---|---|---|---|
| ASD | 0.039 | 0.099 | 0.280 |
| MDD | 0.041 | 0.089 | 0.266 |
| BIP | 0.005 | 0.830 | 0.908 |
| SCZ | 0.039 | 0.106 | 0.291 |
| CROSS | 0.038 | 0.107 | 0.291 |
| COMP | 0.036 | 0.134 | 0.302 |
| MP | 0.040 | 0.093 | 0.274 |
| NDV | 0.072 | 0.002 | 0.025 |
| **CBCL Attention** | | | |
| AN | -0.052 | 0.025 | 0.114 |
| OCD | 0.002 | 0.944 | 0.969 |
| TS | 0.021 | 0.355 | 0.567 |
| ADHD | 0.117 | 5.40E-07 | 7.78E-05 |
| ASD | 0.018 | 0.436 | 0.598 |
| MDD | 0.018 | 0.435 | 0.598 |
| BIP | 0.002 | 0.915 | 0.969 |
| SCZ | 0.056 | 0.019 | 0.093 |
| CROSS | 0.037 | 0.116 | 0.302 |
| COMP | -0.003 | 0.893 | 0.960 |
| MP | 0.047 | 0.046 | 0.174 |
| NDV | 0.096 | 4.00E-05 | 0.001 |
| **CBCL Rulebreak** | | | |
| AN | 0.001 | 0.957 | 0.971 |
| OCD | -0.006 | 0.791 | 0.902 |
| TS | 0.018 | 0.449 | 0.604 |
| ADHD | 0.067 | 0.005 | 0.037 |
| ASD | -0.005 | 0.820 | 0.908 |
| MDD | 0.002 | 0.949 | 0.969 |
| BIP | 0.032 | 0.182 | 0.365 |
| SCZ | 0.013 | 0.577 | 0.745 |
| CROSS | 0.044 | 0.062 | 0.207 |
| COMP | 0.021 | 0.372 | 0.576 |
| MP | 0.037 | 0.124 | 0.302 |
| NDV | 0.064 | 0.007 | 0.045 |
| **CBCL Aggressive** | | | |
| AN | 0.026 | 0.270 | 0.457 |
| OCD | 0.012 | 0.625 | 0.786 |
| TS | 0.036 | 0.123 | 0.302 |
| ADHD | 0.098 | 3.20E-05 | 0.001 |
| ASD | 0.042 | 0.082 | 0.256 |
| MDD | 0.027 | 0.265 | 0.457 |
| BIP | 0.029 | 0.220 | 0.407 |
| SCZ | 0.057 | 0.019 | 0.093 |
| CROSS | 0.058 | 0.015 | 0.085 |
| COMP | 0.036 | 0.130 | 0.302 |

| | | | |
|---|---|---|---|
| MP | 0.071 | 0.003 | 0.032 |
| NDV | 0.103 | 1.30E-05 | 4.68E-04 |
| **CBCL Anx/Dep** | | | |
| AN | 0.067 | 0.005 | 0.037 |
| OCD | 0.026 | 0.274 | 0.459 |
| TS | 0.020 | 0.386 | 0.585 |
| ADHD | 0.049 | 0.037 | 0.148 |
| ASD | 0.030 | 0.206 | 0.394 |
| MDD | 0.025 | 0.293 | 0.486 |
| BIP | -0.002 | 0.947 | 0.969 |
| SCZ | 0.034 | 0.161 | 0.336 |
| CROSS | 0.028 | 0.233 | 0.419 |
| COMP | 0.064 | 0.008 | 0.047 |
| MP | 0.036 | 0.131 | 0.302 |
| NDV | 0.050 | 0.033 | 0.137 |
| **CBCL With/Dep** | | | |
| AN | 0.010 | 0.671 | 0.832 |
| OCD | 0.021 | 0.383 | 0.585 |
| TS | 0.019 | 0.428 | 0.598 |
| ADHD | 0.020 | 0.407 | 0.591 |
| ASD | 0.019 | 0.434 | 0.598 |
| MDD | 0.021 | 0.371 | 0.576 |
| BIP | 0.008 | 0.749 | 0.884 |
| SCZ | 0.022 | 0.354 | 0.567 |
| CROSS | 0.035 | 0.145 | 0.322 |
| COMP | 0.032 | 0.185 | 0.365 |
| MP | 0.013 | 0.579 | 0.745 |
| NDV | 0.042 | 0.075 | 0.244 |
| **CBCL Somatic** | | | |
| AN | 0.005 | 0.832 | 0.908 |
| OCD | -0.037 | 0.121 | 0.302 |
| TS | 0.029 | 0.208 | 0.394 |
| ADHD | 0.094 | 6.20E-05 | 0.001 |
| ASD | 0.047 | 0.047 | 0.174 |
| MDD | 0.054 | 0.022 | 0.103 |
| BIP | 0.009 | 0.709 | 0.865 |
| SCZ | 0.038 | 0.114 | 0.302 |
| CROSS | 0.030 | 0.204 | 0.394 |
| COMP | -0.022 | 0.365 | 0.576 |
| MP | 0.047 | 0.048 | 0.174 |
| NDV | 0.084 | 4.00E-04 | 0.005 |
| **CBCL Social** | | | |
| AN | 0.008 | 0.732 | 0.880 |
| OCD | -0.018 | 0.464 | 0.618 |
| TS | 0.034 | 0.152 | 0.333 |

| | | | |
|---|---|---|---|
| ADHD | 0.088 | 2.00E-04 | 0.003 |
| ASD | 0.019 | 0.430 | 0.598 |
| MDD | 0.002 | 0.935 | 0.969 |
| BIP | -0.004 | 0.856 | 0.927 |
| SCZ | 0.006 | 0.810 | 0.904 |
| CROSS | 0.008 | 0.733 | 0.880 |
| COMP | 0.013 | 0.580 | 0.745 |
| MP | 0.006 | 0.795 | 0.902 |
| NDV | 0.076 | 0.001 | 0.016 |
| **CBCL Thought** | | | |
| AN | 0.019 | 0.420 | 0.598 |
| OCD | -0.002 | 0.937 | 0.969 |
| TS | 0.024 | 0.314 | 0.514 |
| ADHD | 0.056 | 0.018 | 0.093 |
| ASD | 0.047 | 0.049 | 0.174 |
| MDD | 0.041 | 0.084 | 0.256 |
| BIP | -0.006 | 0.810 | 0.904 |
| SCZ | 0.055 | 0.023 | 0.108 |
| CROSS | 0.020 | 0.402 | 0.590 |
| COMP | 0.026 | 0.270 | 0.457 |
| MP | 0.052 | 0.029 | 0.127 |
| NDV | 0.066 | 0.005 | 0.037 |
| **Psychosis spectrum** | | | |
| AN | 0.000 | 0.996 | 0.999 |
| OCD | -0.103 | 0.179 | 0.362 |
| TS | 0.005 | 0.942 | 0.969 |
| ADHD | 0.302 | 7.40E-05 | 0.001 |
| ASD | 0.046 | 0.545 | 0.720 |
| MDD | 0.057 | 0.445 | 0.604 |
| BIP | -0.131 | 0.080 | 0.254 |
| SCZ | 0.029 | 0.703 | 0.865 |
| CROSS | 0.090 | 0.236 | 0.419 |
| COMP | -0.024 | 0.749 | 0.884 |
| MP | 0.020 | 0.794 | 0.902 |
| NDV | 0.212 | 0.005 | 0.037 |

## SUPPLEMENTAL TABLE 8. Results from R-squared change models in Generation R

Model 1: covariates only

Model 2: covariates + PGS of interest

Model 3: covariates + PGS of interest + NDV PGS

**Included covariates**

Fixed effects: age, sex, top 5 principal components

| | R2 (*Model 1* | R2 (*Model 2* | P (*Model 2* ) | R2 (*Model 3* | P (*Model 3* ) |
|---|---|---|---|---|---|
| **Age 9 CBCL Total** | | | | | |
| AN | 0.012 | 0.013 | 0.367 | 0.025 | 2.66E-06 |
| OCD | 0.012 | 0.012 | 0.787 | 0.023 | 6.02E-06 |
| TS | 0.012 | 0.014 | 0.087 | 0.024 | 1.33E-05 |
| ADHD | 0.012 | 0.021 | 3.59E-05 | 0.024 | 2.41E-02 |
| ASD | 0.012 | 0.014 | 0.116 | 0.024 | 1.46E-05 |
| MDD | 0.012 | 0.015 | 1.61E-02 | 0.024 | 8.14E-05 |
| BIP | 0.012 | 0.013 | 0.312 | 0.025 | 2.20E-06 |
| SCZ | 0.012 | 0.016 | 0.008 | 0.025 | 2.82E-05 |
| CROSS | 0.012 | 0.013 | 0.399 | 0.024 | 6.79E-06 |
| COMPULS | 0.012 | 0.012 | 0.608 | 0.023 | 6.63E-06 |
| MOODPSYCH | 0.012 | 0.015 | 0.041 | 0.024 | 3.74E-05 |
| | | | | | |
| **Age 9 CBCL External** | | | | | |
| AN | 0.012 | 0.012 | 0.433 | 0.021 | 5.94E-05 |
| OCD | 0.012 | 0.012 | 0.809 | 0.020 | 1.09E-04 |
| TS | 0.012 | 0.013 | 0.112 | 0.021 | 2.09E-04 |
| ADHD | 0.012 | 0.017 | 2.66E-03 | 0.020 | 0.014 |
| ASD | 0.012 | 0.014 | 0.071 | 0.020 | 5.94E-04 |
| MDD | 0.012 | 0.013 | 0.090 | 0.021 | 2.16E-04 |
| BIP | 0.012 | 0.012 | 0.698 | 0.020 | 7.04E-05 |
| SCZ | 0.012 | 0.015 | 0.021 | 0.022 | 3.54E-04 |
| CROSS | 0.012 | 0.012 | 0.438 | 0.020 | 1.29E-04 |
| COMPULS | 0.012 | 0.012 | 0.726 | 0.020 | 1.14E-04 |
| MOODPSYCH | 0.012 | 0.014 | 0.041 | 0.020 | 5.87E-04 |
| | | | | | |
| **Age 9 CBCL Internal** | | | | | |
| AN | 0.002 | 0.002 | 0.965 | 0.004 | 0.049 |
| OCD | 0.002 | 0.002 | 0.666 | 0.004 | 5.27E-02 |
| TS | 0.002 | 0.002 | 0.568 | 0.004 | 5.78E-02 |
| ADHD | 0.002 | 0.004 | 0.112 | 0.004 | 0.244 |
| ASD | 0.002 | 0.003 | 0.432 | 0.004 | 0.071 |
| MDD | 0.002 | 0.004 | 0.075 | 0.005 | 0.308 |

| | | | | | |
|---|---|---|---|---|---|
| BIP | 0.002 | 0.003 | 0.223 | 0.006 | 3.11E-02 |
| SCZ | 0.002 | 0.004 | 0.101 | 0.005 | 8.41E-02 |
| CROSS | 0.002 | 0.002 | 0.639 | 0.004 | 0.057 |
| COMPULS | 0.002 | 0.002 | 0.673 | 0.004 | 5.44E-02 |
| MOODPSYCH | 0.002 | 0.003 | 0.320 | 0.004 | 0.081 |
| | | | | | |
| **Age 9 Psychosis Spectrum** | | | | | |
| AN | 0.004 | 0.004 | 0.833 | 0.005 | 1.62E-01 |
| OCD | 0.004 | 0.004 | 0.793 | 0.005 | 1.53E-01 |
| TS | 0.004 | 0.005 | 0.114 | 0.006 | 2.12E-01 |
| ADHD | 0.004 | 0.004 | 0.814 | 0.006 | 7.58E-02 |
| ASD | 0.004 | 0.004 | 0.872 | 0.005 | 1.22E-01 |
| MDD | 0.004 | 0.004 | 8.58E-01 | 0.006 | 8.12E-02 |
| BIP | 0.004 | 0.005 | 0.140 | 0.006 | 9.85E-02 |
| SCZ | 0.004 | 0.004 | 0.962 | 0.005 | 1.54E-01 |
| CROSS | 0.004 | 0.004 | 0.523 | 0.006 | 8.73E-02 |
| COMPULS | 0.004 | 0.004 | 0.852 | 0.005 | 1.51E-01 |
| MOODPSYCH | 0.004 | 0.005 | 0.468 | 0.006 | 9.10E-02 |
| | | | | | |
| **Age 13 CBCL Total** | | | | | |
| AN | 0.006 | 0.007 | 0.628 | 0.018 | 6.09E-06 |
| OCD | 0.006 | 0.006 | 0.998 | 0.018 | 5.32E-06 |
| TS | 0.006 | 0.008 | 0.134 | 0.018 | 1.01E-05 |
| ADHD | 0.006 | 0.018 | 3.25E-06 | 0.020 | 8.24E-02 |
| ASD | 0.006 | 0.008 | 0.121 | 0.018 | 1.25E-05 |
| MDD | 0.006 | 0.008 | 0.134 | 0.021 | 1.41E-06 |
| BIP | 0.006 | 0.007 | 0.599 | 0.018 | 6.08E-06 |
| SCZ | 0.006 | 0.009 | 0.030 | 0.019 | 2.02E-05 |
| CROSS | 0.006 | 0.009 | 0.049 | 0.018 | 3.50E-05 |
| COMPULS | 0.006 | 0.007 | 0.251 | 0.018 | 7.80E-06 |
| MOODPSYCH | 0.006 | 0.010 | 0.015 | 0.019 | 5.75E-05 |
| | | | | | |
| **Age 13 CBCL External** | | | | | |
| AN | 0.009 | 0.010 | 0.398 | 0.019 | 4.28E-05 |
| OCD | 0.009 | 0.009 | 0.786 | 0.019 | 3.25E-05 |
| TS | 0.009 | 0.010 | 0.158 | 0.019 | 5.47E-05 |
| ADHD | 0.009 | 0.018 | 4.98E-05 | 0.020 | 0.072 |
| ASD | 0.009 | 0.010 | 0.219 | 0.020 | 3.95E-05 |
| MDD | 0.009 | 0.010 | 0.393 | 0.023 | 9.78E-07 |
| BIP | 0.009 | 0.010 | 0.176 | 0.019 | 6.43E-05 |
| SCZ | 0.009 | 0.011 | 0.052 | 0.020 | 9.35E-05 |
| CROSS | 0.009 | 0.013 | 0.015 | 0.020 | 3.55E-04 |
| COMPULS | 0.009 | 0.010 | 0.155 | 0.020 | 4.90E-05 |
| MOODPSYCH | 0.009 | 0.013 | 0.007 | 0.020 | 4.06E-04 |
| | | | | | |

| | | | | | |
|---|---|---|---|---|---|
| **Age 13 CBCL Internal** | | | | | |
| AN | 0.005 | 0.006 | 0.098 | 0.011 | 4.12E-03 |
| OCD | 0.005 | 0.005 | 0.761 | 0.010 | 2.43E-03 |
| TS | 0.005 | 0.006 | 0.231 | 0.010 | 3.40E-03 |
| ADHD | 0.005 | 0.009 | 0.005 | 0.010 | 0.146 |
| ASD | 0.005 | 0.006 | 0.099 | 0.010 | 0.011 |
| MDD | 0.005 | 0.006 | 0.089 | 0.010 | 0.010 |
| BIP | 0.005 | 0.005 | 0.830 | 0.010 | 2.31E-03 |
| SCZ | 0.005 | 0.006 | 0.106 | 0.011 | 4.77E-03 |
| CROSS | 0.005 | 0.006 | 0.107 | 0.010 | 7.69E-03 |
| COMPULS | 0.005 | 0.006 | 0.134 | 0.011 | 3.40E-03 |
| MOODPSYCH | 0.005 | 0.006 | 0.093 | 0.010 | 7.56E-03 |
| | | | | | |
| **Age 13 Psychosis Spectrum** | | | | | |
| AN | 0.002 | 0.002 | 0.998 | 0.007 | 4.58E-03 |
| OCD | 0.002 | 0.003 | 0.179 | 0.009 | 4.31E-03 |
| TS | 0.002 | 0.002 | 0.942 | 0.007 | 4.70E-03 |
| ADHD | 0.002 | 0.013 | 6.64E-05 | 0.013 | 9.34E-01 |
| ASD | 0.002 | 0.002 | 0.543 | 0.008 | 3.65E-03 |
| MDD | 0.002 | 0.002 | 4.45E-01 | 0.009 | 1.69E-03 |
| BIP | 0.002 | 0.004 | 0.079 | 0.011 | 1.68E-03 |
| SCZ | 0.002 | 0.002 | 0.701 | 0.007 | 5.26E-03 |
| CROSS | 0.002 | 0.003 | 0.236 | 0.007 | 1.01E-02 |
| COMPULS | 0.002 | 0.002 | 0.750 | 0.008 | 4.38E-03 |
| MOODPSYCH | 0.002 | 0.002 | 0.794 | 0.008 | 4.21E-03 |

## SUPPLEMENTAL TABLE 9. Effect sizes, and p- and q-values of PGS-GFP relationships (ABCD, age 9-10)

p-values in red reflect significance after FDR correction

All statistics shown are derived from two-sided linear mixed effects regressions

**N = 4459**

**Included covariates**

Fixed effects: age, sex, top 5 principal components

Random effects: site

***GFP2:*** bifactor model with one "p" factor and 8 orthogonal subfactors

FDR corrects for 9 scales x 12 PGS = 108 comparisons

***GFP3:*** bifactor model with one "p" factor and 2 orthogonal subfactors

FDR corrects for 3 scales x 12 PGS = 36 comparisons

| ***GFP2*** | *Beta* | *p (uncorrected)* | *p (FDR)* |
|---|---|---|---|
| **General Factor** | | | |
| AN | -0.027 | 0.060 | 0.335 |
| OCD | 0.003 | 0.817 | 0.926 |
| TS | 0.009 | 0.516 | 0.826 |
| ADHD | 0.049 | 2.49E-04 | 0.009 |
| ASD | 0.012 | 0.398 | 0.755 |
| MDD | 0.048 | 0.001 | 0.015 |
| BIP | 0.02 | 0.158 | 0.504 |
| SCZ | 0.031 | 0.063 | 0.335 |
| CROSS | 0.032 | 0.021 | 0.175 |
| COMP | -0.007 | 0.595 | 0.876 |
| MP | 0.041 | 0.011 | 0.140 |
| NDV | 0.06 | *1.40E-05 | 0.001 |
| | **R2 change for (other PRS only) vs. (other PRS + NDV): NDV vs. ADHD, p=.012 ; NDV vs. MDD, p=.005* | | |
| **Withdrawn** | | | |
| AN | 0.002 | 0.920 | 0.967 |
| OCD | 0.019 | 0.213 | 0.568 |
| TS | -0.01 | 0.495 | 0.818 |
| ADHD | -0.017 | 0.262 | 0.618 |
| ASD | 0.019 | 0.220 | 0.569 |
| MDD | -0.003 | 0.848 | 0.926 |

| | | | |
|---|---|---|---|
| BIP | -0.003 | 0.852 | 0.926 |
| SCZ | -0.013 | 0.472 | 0.818 |
| CROSS | 0.003 | 0.843 | 0.926 |
| COMP | 0.008 | 0.575 | 0.862 |
| MP | -0.009 | 0.626 | 0.882 |
| NDV | -0.011 | 0.485 | 0.818 |
| **Somatic Complaints** | | | |
| AN | 0.031 | 0.048 | 0.312 |
| OCD | 0.006 | 0.674 | 0.899 |
| TS | 0.017 | 0.253 | 0.607 |
| ADHD | 0.022 | 0.133 | 0.481 |
| ASD | 0.027 | 0.076 | 0.365 |
| MDD | 0.038 | 0.015 | 0.145 |
| BIP | -0.021 | 0.181 | 0.518 |
| SCZ | -0.011 | 0.538 | 0.833 |
| CROSS | 0.046 | 0.003 | 0.055 |
| COMP | 0.006 | 0.678 | 0.899 |
| MP | -0.001 | 0.977 | 0.991 |
| NDV | 0.032 | 0.036 | 0.249 |
| **Anxious/Depressed** | | | |
| AN | 0.04 | 0.017 | 0.151 |
| OCD | 0.023 | 0.148 | 0.495 |
| TS | 0.011 | 0.507 | 0.820 |
| ADHD | -0.026 | 0.095 | 0.405 |
| ASD | -0.005 | 0.777 | 0.926 |
| MDD | 0.004 | 0.816 | 0.926 |
| BIP | 0.015 | 0.364 | 0.723 |
| SCZ | 0.03 | 0.120 | 0.455 |
| CROSS | 0.015 | 0.367 | 0.723 |
| COMP | 0.041 | 0.010 | 0.140 |
| MP | 0.035 | 0.061 | 0.335 |
| NDV | -0.013 | 0.409 | 0.765 |
| **Social Problems** | | | |
| AN | -0.01 | 0.525 | 0.831 |
| OCD | -0.018 | 0.233 | 0.578 |
| TS | 0.006 | 0.672 | 0.899 |
| ADHD | 0.005 | 0.731 | 0.926 |
| ASD | 0.007 | 0.631 | 0.882 |
| MDD | 0.014 | 0.374 | 0.723 |
| BIP | 0.006 | 0.687 | 0.899 |
| SCZ | -0.029 | 0.103 | 0.415 |
| CROSS | 0.004 | 0.794 | 0.926 |
| COMP | -0.015 | 0.310 | 0.676 |
| MP | -0.016 | 0.355 | 0.723 |
| NDV | 0.008 | 0.570 | 0.862 |

| | | | |
|---|---|---|---|
| **Thought Problems** | | | |
| AN | -0.011 | 0.462 | 0.818 |
| OCD | 0.003 | 0.839 | 0.926 |
| TS | 0.009 | 0.491 | 0.818 |
| ADHD | 0.004 | 0.756 | 0.926 |
| ASD | 0.01 | 0.462 | 0.818 |
| MDD | -0.013 | 0.350 | 0.723 |
| BIP | -0.006 | 0.680 | 0.899 |
| SCZ | -0.004 | 0.802 | 0.926 |
| CROSS | 0.024 | 0.090 | 0.405 |
| COMP | 0.007 | 0.596 | 0.876 |
| MP | 0.003 | 0.835 | 0.926 |
| NDV | 0 | 0.973 | 0.991 |
| **Attention Problems** | | | |
| AN | -0.023 | 0.126 | 0.464 |
| OCD | 0.01 | 0.492 | 0.818 |
| TS | 0.011 | 0.463 | 0.818 |
| ADHD | -0.007 | 0.611 | 0.879 |
| ASD | 0.019 | 0.180 | 0.518 |
| MDD | -0.005 | 0.750 | 0.926 |
| BIP | -0.013 | 0.377 | 0.723 |
| SCZ | 0.006 | 0.748 | 0.926 |
| CROSS | 0.01 | 0.500 | 0.818 |
| COMP | -0.006 | 0.677 | 0.899 |
| MP | 0.01 | 0.571 | 0.862 |
| NDV | 0.002 | 0.892 | 0.955 |
| **Delinquent Behav.** | | | |
| AN | -0.023 | 0.096 | 0.405 |
| OCD | -0.001 | 0.954 | 0.987 |
| TS | 0.002 | 0.853 | 0.926 |
| ADHD | 0.004 | 0.741 | 0.926 |
| ASD | -0.001 | 0.959 | 0.987 |
| MDD | 0.035 | 0.012 | 0.140 |
| BIP | 0.02 | 0.144 | 0.492 |
| SCZ | 0.023 | 0.156 | 0.504 |
| CROSS | 0.018 | 0.187 | 0.518 |
| COMP | -0.004 | 0.736 | 0.926 |
| MP | 0.034 | 0.032 | 0.229 |
| NDV | 0.018 | 0.192 | 0.521 |
| **Aggression** | | | |
| AN | 0.028 | 0.069 | 0.354 |
| OCD | 0.015 | 0.328 | 0.705 |
| TS | 0.008 | 0.608 | 0.879 |
| ADHD | 0.003 | 0.855 | 0.926 |
| ASD | 0 | 0.988 | 0.995 |

| | | | |
|---|---|---|---|
| MDD | 0.004 | 0.796 | 0.926 |
| BIP | -0.002 | 0.908 | 0.961 |
| SCZ | 0.009 | 0.625 | 0.882 |
| CROSS | 0.017 | 0.249 | 0.607 |
| COMP | 0.022 | 0.143 | 0.492 |
| MP | -0.001 | 0.957 | 0.987 |
| NDV | -0.007 | 0.650 | 0.899 |

| ***GFP3*** | Beta | p (uncorrected) | FDR |
|---|---|---|---|
| **General Factor** | | | |
| AN | -0.032 | 0.023 | 0.185 |
| OCD | 0.003 | 0.835 | 0.926 |
| TS | 0.009 | 0.531 | 0.831 |
| ADHD | 0.051 | 1.54E-04 | 0.007 |
| ASD | 0.014 | 0.292 | 0.647 |
| MDD | 0.048 | 0.001 | 0.015 |
| BIP | 0.019 | 0.186 | 0.518 |
| SCZ | 0.027 | 0.104 | 0.415 |
| CROSS | 0.035 | 0.012 | 0.140 |
| COMP | -0.009 | 0.490 | 0.818 |
| MP | 0.039 | 0.014 | 0.141 |
| NDV | 0.061 | **6.77E-06 | 0.001 |
| | ***R2 change for (other PRS only) vs. (other PRS + NDV): NDV vs. ADHD, p=.009 ; NDV vs. MDD, p=.003* | | |
| **Internalizing** | | | |
| AN | 0.046 | 0.005 | 0.082 |
| OCD | 0.027 | 0.086 | 0.398 |
| TS | 0.019 | 0.229 | 0.578 |
| ADHD | -0.022 | 0.163 | 0.510 |
| ASD | 0.015 | 0.355 | 0.723 |
| MDD | 0.02 | 0.221 | 0.569 |
| BIP | -0.003 | 0.848 | 0.926 |
| SCZ | 0.02 | 0.292 | 0.647 |
| CROSS | 0.035 | 0.028 | 0.214 |
| COMP | 0.038 | 0.013 | 0.141 |
| MP | 0.025 | 0.175 | 0.518 |
| NDV | 0 | 0.997 | 0.997 |
| **Externalizing** | | | |
| AN | 0.017 | 0.276 | 0.632 |
| OCD | 0.016 | 0.276 | 0.632 |
| TS | 0.013 | 0.371 | 0.723 |
| ADHD | 0.004 | 0.771 | 0.926 |
| ASD | 0.002 | 0.895 | 0.955 |
| MDD | 0.027 | 0.072 | 0.359 |

| | | | |
|---|---|---|---|
| BIP | 0.012 | 0.435 | 0.804 |
| SCZ | 0.034 | 0.053 | 0.315 |
| CROSS | 0.029 | 0.052 | 0.315 |
| COMP | 0.02 | 0.181 | 0.518 |
| MP | 0.027 | 0.120 | 0.455 |
| NDV | 0.003 | 0.834 | 0.926 |

## SUPPLEMENTAL TABLE 10. List of top FDR significant Neurodevelopmental genes (q<0.05)

List of top NDV genes as annotated by MAGMA
p-values calculated from snp-wise mean models which uses the sum of the squared SNP Z-statistics (from GWAS-derived summary statistics) as the test statistic; H0 = no gene association with phenotype

| *Gene symbol* | *Ensembl ID* | *CHR* | *START* | *STOP* | *NSNPS* | *NPARAM* |
|---|---|---|---|---|---|---|
| SORCS3 | ENSG00000156395 | 10 | 106365859 | 107034993 | 1795 | 46 |
| DUSP6 | ENSG00000139318 | 12 | 89731009 | 89782048 | 134 | 17 |
| SEMA6D | ENSG00000137872 | 15 | 47441298 | 48076420 | 1525 | 56 |
| CUBN | ENSG00000107611 | 10 | 16855963 | 17206830 | 1211 | 79 |
| CCDC71 | ENSG00000177352 | 3 | 49189968 | 49238754 | 60 | 7 |
| SLC30A9 | ENSG00000014824 | 4 | 41957489 | 42102474 | 450 | 12 |
| CCDC36 | ENSG00000173421 | 3 | 49200861 | 49305537 | 121 | 10 |
| C3orf84 | ENSG00000236980 | 3 | 49205065 | 49264291 | 69 | 7 |
| ST3GAL3 | ENSG00000126091 | 1 | 44136495 | 44406831 | 514 | 19 |
| RP11-3B7.1 | ENSG00000225399 | 3 | 49262518 | 49308744 | 51 | 8 |
| KLHDC8B | ENSG00000185909 | 3 | 49174044 | 49223917 | 61 | 8 |
| LAMB2 | ENSG00000172037 | 3 | 49148547 | 49205551 | 53 | 9 |
| FOXP2 | ENSG00000128573 | 7 | 113691382 | 114343827 | 838 | 44 |
| VSIG10 | ENSG00000176834 | 12 | 118491398 | 118608831 | 307 | 38 |
| C3orf62 | ENSG00000188315 | 3 | 49296035 | 49350342 | 61 | 10 |
| PTPN1 | ENSG00000196396 | 20 | 49091891 | 49211299 | 288 | 22 |
| CACNA2D1 | ENSG00000153956 | 7 | 81565760 | 82108114 | 1425 | 92 |
| MPL | ENSG00000117400 | 1 | 43768478 | 43828443 | 59 | 6 |
| TIE1 | ENSG00000066056 | 1 | 43731664 | 43798779 | 96 | 17 |
| C1orf210 | ENSG00000253313 | 1 | 43737554 | 43786288 | 75 | 14 |
| BEND4 | ENSG00000188848 | 4 | 42102955 | 42189895 | 232 | 15 |
| USP19 | ENSG00000172046 | 3 | 49135479 | 49193371 | 50 | 8 |
| PTPRF | ENSG00000142949 | 1 | 43955858 | 44099343 | 296 | 15 |
| BSN | ENSG00000164061 | 3 | 49556922 | 49718978 | 192 | 14 |
| USP4 | ENSG00000114316 | 3 | 49305264 | 49413145 | 134 | 13 |
| PEBP1 | ENSG00000089220 | 12 | 118538663 | 118593389 | 135 | 19 |
| KDM4A | ENSG00000066135 | 1 | 44080829 | 44181186 | 167 | 12 |
| QARS | ENSG00000172053 | 3 | 49123365 | 49177553 | 41 | 9 |
| FAM212A | ENSG00000185614 | 3 | 49805687 | 49852463 | 71 | 10 |
| MANBA | ENSG00000109323 | 4 | 103542660 | 103717151 | 285 | 13 |
| RAD51 | ENSG00000051180 | 15 | 40951972 | 41034354 | 163 | 12 |
| CDC20 | ENSG00000117399 | 1 | 43789626 | 43838874 | 42 | 6 |
| FEZF1 | ENSG00000128610 | 7 | 121931448 | 121985745 | 133 | 16 |
| CHRM2 | ENSG00000181072 | 7 | 136518416 | 136715002 | 509 | 32 |
| ELOVL1 | ENSG00000066322 | 1 | 43819068 | 43868696 | 63 | 9 |
| NRCAM | ENSG00000091129 | 7 | 107778068 | 108132161 | 1131 | 46 |
| C11orf31 | ENSG00000211450 | 11 | 57473825 | 57520986 | 51 | 6 |

| | | | | | | |
|---|---|---|---|---|---|---|
| PCLO | ENSG00000186472 | 7 | 82373329 | 82827246 | 1085 | 43 |
| MED19 | ENSG00000156603 | 11 | 57461186 | 57514693 | 66 | 7 |
| GCHFR | ENSG00000137880 | 15 | 41021218 | 41069906 | 69 | 10 |
| TMX2 | ENSG00000213593 | 11 | 57445072 | 57518445 | 92 | 9 |
| CDHR4 | ENSG00000187492 | 3 | 49818165 | 49872268 | 69 | 9 |
| MAP7D1 | ENSG00000116871 | 1 | 36586180 | 36656450 | 73 | 6 |
| RP11-691N7.6 | ENSG00000254732 | 11 | 57474635 | 57570715 | 108 | 9 |
| UBA7 | ENSG00000182179 | 3 | 49832640 | 49886379 | 56 | 7 |
| AMT | ENSG00000145020 | 3 | 49444211 | 49495186 | 65 | 7 |
| RHCE | ENSG00000188672 | 1 | 25678740 | 25791683 | 107 | 3 |
| TMX2-CTNND | ENSG00000254462 | 11 | 57445077 | 57569058 | 148 | 12 |
| TRAPPC3 | ENSG00000054116 | 1 | 36592173 | 36650098 | 55 | 4 |
| TMEM50A | ENSG00000183726 | 1 | 25629408 | 25698852 | 21 | 3 |
| WDR11 | ENSG00000120008 | 10 | 122575687 | 122679036 | 351 | 22 |
| C1orf63 | ENSG00000117616 | 1 | 25558728 | 25699704 | 40 | 7 |
| OLFM4 | ENSG00000102837 | 13 | 53567894 | 53636192 | 164 | 33 |
| NICN1 | ENSG00000145029 | 3 | 49450379 | 49501759 | 65 | 7 |
| ASTN2 | ENSG00000148219 | 9 | 119177504 | 120212348 | 2855 | 136 |
| RMDN3 | ENSG00000137824 | 15 | 41018082 | 41083049 | 86 | 10 |
| RHOA | ENSG00000067560 | 3 | 49386578 | 49485431 | 148 | 11 |
| FBXL17 | ENSG00000145743 | 5 | 107184736 | 107752799 | 1187 | 37 |
| MED8 | ENSG00000159479 | 1 | 43839588 | 43890479 | 75 | 7 |
| CTNND1 | ENSG00000198561 | 11 | 57485715 | 57597018 | 115 | 10 |
| FOXP1 | ENSG00000114861 | 3 | 70993844 | 71668140 | 1153 | 93 |
| RBPJ | ENSG00000168214 | 4 | 26130077 | 26446541 | 434 | 40 |
| BTBD18 | ENSG00000233436 | 11 | 57500986 | 57554253 | 53 | 8 |
| TMEM57 | ENSG00000204178 | 1 | 25722388 | 25836700 | 115 | 2 |
| ESR2 | ENSG00000140009 | 14 | 64540950 | 64839830 | 673 | 40 |
| SZT2 | ENSG00000198198 | 1 | 43820553 | 43928321 | 138 | 10 |
| TCTA | ENSG00000145022 | 3 | 49414639 | 49463908 | 78 | 8 |
| AC092687.4 | ENSG00000232056 | 2 | 10952703 | 11005081 | 271 | 23 |

| ***Gene symbol*** | ***N*** | ***ZSTAT*** | ***P*** | ***FDR*** |
|---|---|---|---|---|
| SORCS3 | 286955 | 6.2635 | 1.88E-10 | 3.59E-06 |
| DUSP6 | 286955 | 5.7099 | 5.65E-09 | 5.38E-05 |
| SEMA6D | 286955 | 5.0972 | 1.72E-07 | 0.00109435 |
| CUBN | 286955 | 4.9553 | 3.61E-07 | 0.00171997 |
| CCDC71 | 286955 | 4.6794 | 1.44E-06 | 0.00486736 |
| SLC30A9 | 286955 | 4.6249 | 1.87E-06 | 0.00486736 |
| CCDC36 | 286955 | 4.5926 | 2.19E-06 | 0.00486736 |
| C3orf84 | 286955 | 4.5874 | 2.24E-06 | 0.00486736 |
| ST3GAL3 | 286955 | 4.5823 | 2.30E-06 | 0.00486736 |
| RP11-3B7.1 | 286955 | 4.5357 | 2.87E-06 | 0.00547021 |
| KLHDC8B | 286955 | 4.5127 | 3.20E-06 | 0.00554361 |
| LAMB2 | 286955 | 4.3992 | 5.43E-06 | 0.00797619 |
| FOXP2 | 286955 | 4.3988 | 5.44E-06 | 0.00797619 |
| VSIG10 | 286955 | 4.3639 | 6.39E-06 | 0.00823701 |
| C3orf62 | 286955 | 4.3537 | 6.69E-06 | 0.00823701 |
| PTPN1 | 286955 | 4.3465 | 6.92E-06 | 0.00823701 |
| CACNA2D1 | 286955 | 4.2239 | 1.20E-05 | 0.01345407 |
| MPL | 286955 | 4.1729 | 1.50E-05 | 0.01591583 |
| TIE1 | 286955 | 4.1339 | 1.78E-05 | 0.01732589 |
| C1orf210 | 286955 | 4.1294 | 1.82E-05 | 0.01732589 |
| BEND4 | 286955 | 4.1042 | 2.03E-05 | 0.0184006 |
| USP19 | 286955 | 4.0859 | 2.20E-05 | 0.01901476 |
| PTPRF | 286955 | 4.0676 | 2.38E-05 | 0.01938462 |
| BSN | 286955 | 4.0611 | 2.44E-05 | 0.01938462 |
| USP4 | 286955 | 4.0373 | 2.70E-05 | 0.02012404 |
| PEBP1 | 286955 | 4.0336 | 2.75E-05 | 0.02012404 |
| KDM4A | 286955 | 4.0171 | 2.95E-05 | 0.02078926 |
| QARS | 286955 | 4.0014 | 3.15E-05 | 0.02142329 |
| FAM212A | 286955 | 3.9881 | 3.33E-05 | 0.02188286 |
| MANBA | 286955 | 3.9665 | 3.65E-05 | 0.02281692 |
| RAD51 | 286955 | 3.9622 | 3.71E-05 | 0.02281692 |
| CDC20 | 286955 | 3.9415 | 4.05E-05 | 0.02366694 |
| FEZF1 | 286955 | 3.933 | 4.19E-05 | 0.02366694 |
| CHRM2 | 286955 | 3.9287 | 4.27E-05 | 0.02366694 |
| ELOVL1 | 286955 | 3.9244 | 4.35E-05 | 0.02366694 |
| NRCAM | 286955 | 3.9076 | 4.66E-05 | 0.02466334 |
| C11orf31 | 286955 | 3.8998 | 4.81E-05 | 0.02478305 |

| | | | | |
|---|---|---|---|---|
| PCLO | 286955 | 3.8723 | 5.39E-05 | 0.02680616 |
| MED19 | 286955 | 3.868 | 5.49E-05 | 0.02680616 |
| GCHFR | 286955 | 3.8087 | 6.99E-05 | 0.0327792 |
| TMX2 | 286955 | 3.8063 | 7.05E-05 | 0.0327792 |
| CDHR4 | 286955 | 3.7952 | 7.37E-05 | 0.03342297 |
| MAP7D1 | 286955 | 3.7896 | 7.54E-05 | 0.03342297 |
| RP11-691N7.6 | 286955 | 3.7804 | 7.83E-05 | 0.03389957 |
| UBA7 | 286955 | 3.7616 | 8.44E-05 | 0.03573605 |
| AMT | 286955 | 3.7521 | 8.77E-05 | 0.03631311 |
| RHCE | 286955 | 3.7387 | 9.25E-05 | 0.03749474 |
| TMX2-CTNND1 | 286955 | 3.7272 | 9.68E-05 | 0.03842907 |
| TRAPPC3 | 286955 | 3.7141 | 0.00010196 | 0.03888894 |
| TMEM50A | 286955 | 3.7139 | 0.00010206 | 0.03888894 |
| WDR11 | 286955 | 3.7025 | 0.00010676 | 0.03988219 |
| C1orf63 | 286955 | 3.6933 | 0.00011067 | 0.04054779 |
| OLFM4 | 286955 | 3.6856 | 0.00011408 | 0.04100853 |
| NICN1 | 286955 | 3.6799 | 0.00011665 | 0.04115585 |
| ASTN2 | 286955 | 3.6669 | 0.00012275 | 0.0425206 |
| RMDN3 | 286955 | 3.6593 | 0.00012643 | 0.04301329 |
| RHOA | 286955 | 3.629 | 0.00014225 | 0.04715052 |
| FBXL17 | 286955 | 3.6185 | 0.00014814 | 0.04715052 |
| MED8 | 286955 | 3.6182 | 0.00014832 | 0.04715052 |
| CTNND1 | 286955 | 3.6179 | 0.00014849 | 0.04715052 |
| FOXP1 | 286955 | 3.6048 | 0.00015617 | 0.04877624 |
| RBPJ | 286955 | 3.5969 | 0.00016104 | 0.04948603 |
| BTBD18 | 286955 | 3.5921 | 0.00016402 | 0.04960173 |
| TMEM57 | 286955 | 3.5878 | 0.00016677 | 0.04964534 |
| ESR2 | 286955 | 3.5794 | 0.0001722 | 0.04973413 |
| SZT2 | 286955 | 3.5772 | 0.00017363 | 0.04973413 |
| TCTA | 286955 | 3.5736 | 0.00017608 | 0.04973413 |
| AC092687.4 | 286955 | 3.5714 | 0.00017751 | 0.04973413 |

**SUPPLEMENTAL TABLE 11. Gene ontology terms of top 5% most significant Neurodevelopmental genes**

Results from Panther DB's overrepresentation test (Fisher's Exact test): cellular component, molecular function, biological process

# of genes input = 952, # of genes mapped = 881

| *GO cellular component complete* | *Homo sapiens REF #* | *NDV #* | *expected* |
|---|---|---|---|
| dendritic spine (GO:0043197) | 173 | 23 | 7.54 |
| neuron spine (GO:0044309) | 175 | 23 | 7.62 |
| postsynaptic membrane (GO:0045211) | 275 | 27 | 11.98 |
| postsynapse (GO:0098794) | 620 | 53 | 27.01 |
| dendrite (GO:0030425) | 619 | 50 | 26.97 |
| dendritic tree (GO:0097447) | 621 | 50 | 27.06 |
| somatodendritic compartment (GO:0036477) | 845 | 63 | 36.81 |
| neuron projection (GO:0043005) | 1366 | 91 | 59.51 |
| intracellular anatomical structure (GO:0005622) | 14901 | 702 | 649.19 |

| *GO molecular function complete* | *Homo sapiens REF #* | *NDV #* | *expected* |
|---|---|---|---|
| protein binding (GO:0005515) | 14421 | 696 | 627.58 |
| binding (GO:0005488) | 16572 | 791 | 721.19 |
| molecular_function (GO:0003674) | 18282 | 847 | 795.6 |
| *Unclassified (UNCLASSIFIED)* | *2307* | 49 | 100.4 |

| *GO biological process complete* | *Homo sapiens REF #* | *NDV #* | *expected* |
|---|---|---|---|
| regulation of cellular process (GO:0050794) | 11275 | 566 | 490.67 |
| regulation of biological process (GO:0050789) | 11735 | 584 | 510.69 |
| biological regulation (GO:0065007) | 12469 | 612 | 542.63 |

**Homo sapiens REF #:** # of genes in reference set (homo sapiens) that map to GO term
**NDV #:** # of genes in NDV set that map to GO term
**expected:** expected # of genes given number of mapped ref genes (column 2)
**over/under:** over (+) vs under (-) representation
**Fold Enrichment**: NDV # / expected
**raw p-value**: significance value from Fisher's exact test
**FDR**: fdr corrected p-value

| ***over/under*** | ***cell. comp. repeated*** | ***Fold Enrichme*** | ***raw P-value*** | ***FDR*** |
|---|---|---|---|---|
| + | dendritic spine (GO:0043197) | 3.05 | 7.99E-06 | 1.63E-02 |
| + | neuron spine (GO:0044309) | 3.02 | 9.46E-06 | 9.65E-03 |
| + | postsynaptic membrane (GO:004521 | 2.25 | 2.11E-04 | 4.78E-02 |
| + | postsynapse (GO:0098794) | 1.96 | 1.24E-05 | 8.42E-03 |
| + | dendrite (GO:0030425) | 1.85 | 7.21E-05 | 3.68E-02 |
| + | dendritic tree (GO:0097447) | 1.85 | 7.52E-05 | 3.07E-02 |
| + | somatodendritic compartment (GO:( | 1.71 | 8.42E-05 | 2.86E-02 |
| + | neuron projection (GO:0043005) | 1.53 | 1.04E-04 | 2.66E-02 |
| + | intracellular anatomical structure (G | 1.08 | 9.34E-05 | 2.72E-02 |

| ***over/under*** | ***mol. func. repeated*** | ***Fold Enrichme*** | ***raw P-value*** | ***FDR*** |
|---|---|---|---|---|
| + | protein binding (GO:0005515) | 1.11 | 6.30E-07 | 7.73E-04 |
| + | binding (GO:0005488) | 1.1 | 1.10E-09 | 5.41E-06 |
| + | molecular_function (GO:0003674) | 1.06 | 7.72E-09 | 1.89E-05 |
| - | *Unclassified (UNCLASSIFIED)* | 0.49 | 7.72E-09 | 1.26E-05 |

| ***over/under*** | ***bio. proc. repeated*** | ***Fold Enrichme*** | ***raw P-value*** | ***FDR*** |
|---|---|---|---|---|
| + | regulation of cellular process (GO:0C | 1.15 | 6.29E-07 | 9.85E-03 |
| + | regulation of biological process (GO: | 1.14 | 1.10E-06 | 8.60E-03 |
| + | biological regulation (GO:0065007) | 1.13 | 2.70E-06 | 1.41E-02 |

## SUPPLEMENTAL TABLE 12. Abridged version of gene ontology results comparing top 2%, 5%, and 10% most significant Neurodevelopmental genes

Results from Panther DB's overrepresentation test (Fisher's Exact test) at three thresholds of gene inclusion: cellular component, molecular function, biological process

**Fold Enrichment**: NDV # / expected (values < 1 indicate underrepresentation)

**raw p-value**: significance value from Fisher's exact test

**FDR**: fdr corrected p-value

**Top 5%** (copied from Supplemental Table 10)

| *GO cellular component complete* | *Fold Enrichment* | *raw P-value* | *FDR* |
|---|---|---|---|
| dendritic spine (GO:0043197) | 3.05 | 7.99E-06 | 1.63E-02 |
| neuron spine (GO:0044309) | 3.02 | 9.46E-06 | 9.65E-03 |
| postsynaptic membrane (GO:0045211) | 2.25 | 2.11E-04 | 4.78E-02 |
| postsynapse (GO:0098794) | 1.96 | 1.24E-05 | 8.42E-03 |
| dendrite (GO:0030425) | 1.85 | 7.21E-05 | 3.68E-02 |
| dendritic tree (GO:0097447) | 1.85 | 7.52E-05 | 3.07E-02 |
| somatodendritic compartment (GO:0036477) | 1.71 | 8.42E-05 | 2.86E-02 |
| neuron projection (GO:0043005) | 1.53 | 1.04E-04 | 2.66E-02 |
| intracellular anatomical structure (GO:0005622) | 1.08 | 9.34E-05 | 2.72E-02 |
| | | | |
| *GO molecular function complete* | *Fold Enrichment* | *raw P-value* | *FDR* |
| protein binding (GO:0005515) | 1.11 | 6.30E-07 | 7.73E-04 |
| binding (GO:0005488) | 1.1 | 1.10E-09 | 5.41E-06 |
| molecular_function (GO:0003674) | 1.06 | 7.72E-09 | 1.89E-05 |
| *Unclassified (UNCLASSIFIED)* | 0.49 | 7.72E-09 | 1.26E-05 |
| | | | |
| *GO biological process complete* | *Fold Enrichment* | *raw P-value* | *FDR* |
| regulation of cellular process (GO:0050794) | 1.15 | 6.29E-07 | 9.85E-03 |
| regulation of biological process (GO:0050789) | 1.14 | 1.10E-06 | 8.60E-03 |
| biological regulation (GO:0065007) | 1.13 | 2.70E-06 | 1.41E-02 |

**Top 2% NDV GO**

| *GO cellular component complete* | *Fold Enrichment* | *raw P-value* | *FDR* |
|---|---|---|---|
| voltage-gated calcium channel complex (GO:0005891) | 7.73 | 2.15E-04 | 4.89E-02 |
| calcium channel complex (GO:0034704) | 6.53 | 5.76E-05 | 3.93E-02 |
| asymmetric synapse (GO:0032279) | 2.97 | 1.05E-04 | 3.08E-02 |
| postsynaptic density (GO:0014069) | 2.85 | 2.64E-04 | 4.90E-02 |
| neuron to neuron synapse (GO:0098984) | 2.76 | 2.40E-04 | 4.90E-02 |
| postsynapse (GO:0098794) | 2.32 | 1.47E-04 | 3.76E-02 |
| somatodendritic compartment (GO:0036477) | 2.16 | 8.35E-05 | 3.41E-02 |

| | Fold Enrichment | raw P-value | FDR |
|---|---|---|---|
| intracellular membrane-bounded organelle (GO:0043231) | 1.18 | 3.28E-05 | 3.35E-02 |
| membrane-bounded organelle (GO:0043227) | 1.17 | 1.53E-05 | 3.12E-02 |
| intracellular organelle (GO:0043229) | 1.16 | 8.47E-05 | 2.88E-02 |
| organelle (GO:0043226) | 1.14 | 6.66E-05 | 3.40E-02 |
| | | | |
| ***GO molecular function complete*** | ***Fold Enrichment*** | ***raw P-value*** | ***FDR*** |
| protein binding (GO:0005515) | 1.18 | 3.89E-08 | 1.94E-04 |
| binding (GO:0005488) | 1.12 | 8.20E-07 | 2.04E-03 |
| molecular_function (GO:0003674) | 1.07 | 1.64E-05 | 2.73E-02 |
| Unclassified (UNCLASSIFIED) | 0.4 | 1.64E-05 | 2.04E-02 |
| | | | |
| ***GO biological process complete*** | ***Fold Enrichment*** | ***raw P-value*** | ***FDR*** |
| nervous system development (GO:0007399) | 1.83 | 1.07E-06 | 1.68E-02 |

**Top 10% NDV GO**

| ***GO cellular component complete*** | ***Fold Enrichment*** | ***raw P-value*** | ***FDR*** |
|---|---|---|---|
| postsynapse (GO:0098794) | 1.58 | 1.11E-04 | 5.66E-02 |
| membrane-bounded organelle (GO:0043227) | 1.07 | 1.17E-04 | 4.79E-02 |
| organelle (GO:0043226) | 1.07 | 8.04E-05 | 8.21E-02 |
| intracellular anatomical structure (GO:0005622) | 1.06 | 1.91E-04 | 4.89E-02 |
| cellular_component (GO:0005575) | 1.03 | 1.38E-04 | 4.03E-02 |
| Unclassified (UNCLASSIFIED) | 0.7 | 1.38E-04 | 4.70E-02 |
| T cell receptor complex (GO:0042101) | 0.08 | 1.08E-04 | 7.37E-02 |
| immunoglobulin complex (GO:0019814) | < 0.01 | 2.36E-07 | 4.82E-04 |
| | | | |
| ***GO molecular function complete*** | ***Fold Enrichment*** | ***raw P-value*** | ***FDR*** |
| regulation of cellular process (GO:0050794) | 1.12 | 2.49E-07 | 3.90E-03 |
| regulation of biological process (GO:0050789) | 1.11 | 5.08E-07 | 3.99E-03 |
| biological regulation (GO:0065007) | 1.1 | 1.12E-06 | 5.86E-03 |
| cellular process (GO:0009987) | 1.07 | 6.59E-06 | 1.72E-02 |
| biological_process (GO:0008150) | 1.04 | 1.60E-06 | 6.26E-03 |
| Unclassified (UNCLASSIFIED) | 0.71 | 1.60E-06 | 5.01E-03 |
| | | | |
| ***GO biological process complete*** | ***Fold Enrichment*** | ***raw P-value*** | ***FDR*** |
| protein binding (GO:0005515) | 1.08 | 7.96E-08 | 9.90E-05 |
| binding (GO:0005488) | 1.08 | 2.97E-12 | 4.92E-09 |
| molecular_function (GO:0003674) | 1.06 | 3.14E-13 | 7.82E-10 |
| Unclassified (UNCLASSIFIED) | 0.54 | 3.14E-13 | 1.56E-09 |

## SUPPLEMENTAL TABLE 13. Pre- vs postnatal expression differences of Neurodevelopmental and Mood/Psychotic genes across BrainSpan ROIs

Estimates are reported from two-sided mixed effects regressions estimating differences in pre- and postnatal expression allowing gene and donor each their own intercept. Greater (positive) estimates reflect greater expression during prenatal than postnatal life

p-values in red reflect significance after FDR correction

| | **# of top genes (q < 0.05)** | **Proportion of genes in BrainSpan** |
|---|---|---|
| **NDV** | 68 | 56/68 |
| **MP** | 2751 | 2312/2751 |

| | | **NDV** | | | **MP** |
|---|---|---|---|---|---|
| **Region** | *Beta* | *p (uncorr.)* | | *Beta* | *p (uncorr.)* |
| CBC | 0.24327038 | 8.68E-08 | | 0.01191962 | 0.55522583 |
| AMY | 0.0723061 | 0.05987456 | | 0.10131696 | 2.07E-06 |
| MDTHAL | 0.12651768 | 0.00034897 | | 0.07500367 | 0.00011746 |
| HIPP | 0.00730111 | 0.8358202 | | 0.0684607 | 3.99E-05 |
| STR | 0.14866148 | 8.39E-05 | | 0.07984391 | 7.34E-05 |
| NCX | -0.0096959 | 0.68672466 | | 0.0520456 | 0.00117136 |

## SUPPLEMENTAL TABLE 14. Effects of partitioned Neurodevelopmental PGS on measures of psychopathology

Continuous pPGS represent effects of SNPs which map onto genes that are expressed similarly across pre- and postnatal timepoints

Prenatal and postnatal pPGS represent effects of SNPs which map onto genes that are preferentially expressed during and after fetal life respectively

p-values in red reflect significance after FDR correction

All statistics shown are derived from two-sided linear mixed effects regressions

**N = 4459**

**Included covariates**

Fixed effects: age, sex, top 5 principal components

Random effects: site

FDR corrects for 12 scales x 3 PGS = 36 comparisons

| | **Continuous NDV pPGS** | | |
|---|---|---|---|
| **Scale** | *Beta* | *p (uncorr.)* | *p (FDR)* |
| Anx/Dep | 0.037 | 0.01648162 | 0.02966691 |
| With/Dep | 0.029 | 0.04632464 | 0.06706358 |
| Somatic | 0.052 | 0.00036955 | 0.00120943 |
| Social | 0.059 | 3.24E-05 | 0.00014569 |
| Thought | 0.049 | 0.00101668 | 0.00305004 |
| Attention | 0.074 | 2.91E-07 | 5.23E-06 |
| Rulebreak | 0.041 | 0.00276405 | 0.00585328 |
| Aggressive | 0.046 | 0.00121475 | 0.00336391 |
| Internal | 0.054 | 0.0002357 | 0.00084852 |
| External | 0.057 | 8.65E-05 | 0.00034593 |
| Total | 0.076 | 7.64E-08 | 2.75E-06 |
| PQB | 0.058 | 5.03E-06 | 3.02E-05 |
| | | | |
| | **Prenatal NDV PGS** | | |
| **Scale** | *Beta* | *p (uncorr.)* | *p (FDR)* |
| Anx/Dep | 0.015 | 0.32164789 | 0.37187961 |
| With/Dep | 0.014 | 0.32718787 | 0.37187961 |
| Somatic | 0.044 | 0.00264421 | 0.00585328 |
| Social | 0.044 | 0.00175431 | 0.00421033 |
| Thought | 0.044 | 0.00378284 | 0.00756569 |
| Attention | 0.067 | 4.00E-06 | 2.88E-05 |

| | | | |
|---|---|---|---|
| Rulebreak | 0.059 | 1.18E-05 | 6.05E-05 |
| Aggressive | 0.045 | 0.00159482 | 0.00410097 |
| Internal | 0.038 | 0.00999991 | 0.0189472 |
| External | 0.068 | 2.26E-06 | 2.55E-05 |
| Total | 0.066 | 2.83E-06 | 2.55E-05 |
| PQB | 0.026 | 0.03723793 | 0.06093479 |
| | | | |
| | **Postnatal NDV PGS** | | |
| **Scale** | *Beta* | *p (uncorr.)* | *p (FDR)* |
| Anx/Dep | 0.014 | 0.36270838 | 0.39568187 |
| With/Dep | 0.006 | 0.6657531 | 0.68477462 |
| Somatic | 0.03 | 0.04049915 | 0.06338997 |
| Social | 0.007 | 0.63761984 | 0.67512689 |
| Thought | 0.018 | 0.22884977 | 0.28408937 |
| Attention | 0.031 | 0.03536446 | 0.06062479 |
| Rulebreak | 0.024 | 0.07746273 | 0.10328364 |
| Aggressive | -0.001 | 0.92458137 | 0.92458137 |
| Internal | 0.021 | 0.15971134 | 0.20534315 |
| External | 0.014 | 0.33055965 | 0.37187961 |
| Total | 0.026 | 0.06857235 | 0.09494634 |
| PQB | 0.025 | 0.04657193 | 0.06706358 |

## SUPPLEMENTAL TABLE 15. Effects of Neurodevelopmental PGS on global brain measures

p-values in red reflect significance after FDR correction

All statistics shown are derived from two-sided linear mixed effects regressions

**N = 3878**

**Included covariates**

Fixed effects: age, sex, top 5 principal components, ICV, Euler number (surface holes)

Random effects: site, scanner

**FDR** corrects for 4 regions x 4 scales = 16 comparisons

| | **NDV PGS (non-partitioned)** | | |
|---|---|---|---|
| **Region** | *Beta* | *p (uncorr.)* | *p (FDR)* |
| Total Brain Gray Volume | -0.0108264 | 0.10012137 | 0.26699031 |
| Cerebellum Total Gray Volume | -0.0005582 | 0.96183619 | 0.99070632 |
| Cortical Gray Volume | -0.0126001 | 0.08380874 | 0.26699031 |
| Subcortical Gray Volume | -0.0001147 | 0.99070632 | 0.99070632 |
| | | | |
| | **Continuous NDV pPGS** | | |
| | *Beta* | *p (uncorr.)* | *p (FDR)* |
| Total Brain Gray Volume | -0.0120427 | 0.06354014 | 0.25416055 |
| Cerebellum Total Gray Volume | 0.00789618 | 0.49246922 | 0.87550083 |
| Cortical Gray Volume | -0.015331 | 0.03281819 | 0.17503033 |
| Subcortical Gray Volume | 0.00141857 | 0.88384735 | 0.99070632 |
| | | | |
| | **Prenatal NDV pPGS** | | |
| | *Beta* | *p (uncorr.)* | *p (FDR)* |
| Total Brain Gray Volume | -0.0026592 | 0.68099284 | 0.99070632 |
| Cerebellum Total Gray Volume | -0.0045266 | 0.69285341 | 0.99070632 |
| Cortical Gray Volume | -0.0017184 | 0.8103128 | 0.99070632 |
| Subcortical Gray Volume | 0.00034996 | 0.97113748 | 0.99070632 |
| | | | |
| | **Postnatal NDV pPGS** | | |
| | *Beta* | *p (uncorr.)* | *p (FDR)* |
| Total Brain Gray Volume | -0.0141401 | 0.02922331 | 0.17503033 |
| Cerebellum Total Gray Volume | 0.01033894 | 0.36836927 | 0.73673855 |
| Cortical Gray Volume | -0.0193153 | 0.00710554 | 0.11368858 |
| Subcortical Gray Volume | 0.01036102 | 0.28551963 | 0.6526163 |

**SUPPLEMENTAL TABLE 16. Effects of global measures of brain volume on CBCL broadband and PQ-BC scores**

p-values in red reflect significance after FDR correction

All statistics shown are derived from two-sided linear mixed effects regressions

**N (CBCL models) = 10040**

**N (PQB model) = 10048**

**Included covariates**

Fixed effects: age, sex, ICV, Euler number (surface holes)

Random effects: site, scanner, family ID

**FDR** corrects for 4 regions x 4 scales = 16 comparisons

| | **CBCL Total** | | |
|---|---|---|---|
| **Region** | *Beta* | *p (uncorr.)* | *p (FDR)* |
| Total Brain Gray Volume | -1.7365392 | 1.02E-11 | 5.46E-11 |
| Cerebellum Total Gray Volume | -0.3874186 | 0.01224221 | 0.01506733 |
| Cortical Gray Volume | -1.4641491 | 2.91E-10 | 1.16E-09 |
| Subcortical Gray Volume | -0.5595139 | 0.00203422 | 0.00361639 |
| | | | |
| | **CBCL Internalizing** | | |
| | *Beta* | *p (uncorr.)* | *p (FDR)* |
| Total Brain Gray Volume | -0.6089481 | 0.01219232 | 0.01506733 |
| Cerebellum Total Gray Volume | 0.03996863 | 0.78656377 | 0.78656377 |
| Cortical Gray Volume | -0.5851269 | 0.00816875 | 0.01188182 |
| Subcortical Gray Volume | -0.1469999 | 0.39559254 | 0.42196537 |
| | | | |
| | **CBCL Externalizing** | | |
| | *Beta* | *p (uncorr.)* | *p (FDR)* |
| Total Brain Gray Volume | -1.9560749 | 2.22E-16 | 3.55E-15 |
| Cerebellum Total Gray Volume | -0.5487186 | 0.00012337 | 0.00028198 |
| Cortical Gray Volume | -1.6165688 | 9.24E-14 | 7.39E-13 |
| Subcortical Gray Volume | -0.570748 | 0.00072128 | 0.00144255 |
| | | | |
| | **PQ-BC** | | |
| | *Beta* | *p (uncorr.)* | *p (FDR)* |
| Total Brain Gray Volume | -1.6208816 | 4.11E-08 | 1.31E-07 |
| Cerebellum Total Gray Volume | -0.4925723 | 0.00485335 | 0.00776535 |
| Cortical Gray Volume | -1.2392613 | 3.81E-06 | 1.01E-05 |
| Subcortical Gray Volume | -0.500219 | 0.01602817 | 0.0183179 |

## SUPPLEMENTAL TABLE 17. Effects of cerebellar subfield volumes on CBCL broadband and PQ-BC

p-values in red reflect significance after FDR correction
All statistics shown are derived from two-sided linear mixed effects regressions
**N (CBCL models) = 10040**
**N (PQB model) = 10048**

**Included covariates**
Fixed effects: age, sex, ICV, Euler number (surface holes)
Random effects: site, scanner, family ID

**FDR** corrects for 17 regions x 4 scales = 68 comparisons

| | **CBCL Total** | | |
|---|---|---|---|
| **Region** | *Beta* | *p (uncorr.)* | *p (FDR)* |
| Right lobules I-V | -0.0323378 | 0.79995897 | 0.8499564 |
| Right lobule VI | -0.032803 | 0.78429142 | 0.84653677 |
| Right lobule VIIf | -0.1904116 | 0.12962276 | 0.32645731 |
| Right lobules VIIt-VIIE | -0.3491086 | 0.00630455 | 0.06151314 |
| Right lobule VIII | -0.1095904 | 0.40144365 | 0.56871183 |
| Right lobule IX | -0.1274133 | 0.2788423 | 0.51246692 |
| Right lobule X | -0.0979672 | 0.43389678 | 0.59009962 |
| Left lobules I-V | -0.276821 | 0.04045786 | 0.22926121 |
| Left lobule VI | 0.02243531 | 0.84855364 | 0.88738361 |
| Left lobule VIIf | -0.1916537 | 0.12735276 | 0.32645731 |
| Left lobules VIIt-VIIB | -0.2462851 | 0.05712805 | 0.25898048 |
| Left lobule VIII | -0.3264021 | 0.0131264 | 0.09835944 |
| Left lobule IX | -0.072125 | 0.5360047 | 0.67564146 |
| Left lobule X | -0.1040661 | 0.38130002 | 0.5636609 |
| Vermis I-V | -0.2288867 | 0.08013449 | 0.26474253 |
| Vermis VI-VII | -0.0701171 | 0.54647471 | 0.67564146 |
| Vermis VIII-X | -0.0741994 | 0.56595882 | 0.68723571 |
| | | | |
| | **CBCL Internalizing** | | |
| | *Beta* | *p (uncorr.)* | *p (FDR)* |
| Right lobules I-V | 0.17629928 | 0.14978513 | 0.36376389 |
| Right lobule VI | 0.10543793 | 0.35791961 | 0.5636609 |
| Right lobule VIIf | -0.0210333 | 0.86128409 | 0.88738361 |
| Right lobules VIIt-VIIE | -0.1723808 | 0.15892714 | 0.37116962 |
| Right lobule VIII | 0.17410196 | 0.1637513 | 0.37116962 |
| Right lobule IX | 0.04503169 | 0.68908568 | 0.78096377 |
| Right lobule X | 0.0596052 | 0.61920858 | 0.73870497 |
| Left lobules I-V | 0.06026965 | 0.64199262 | 0.7399237 |
| Left lobule VI | 0.20132187 | 0.07393039 | 0.26474253 |

| | | | |
|---|---|---|---|
| Left lobule VIIf | -0.0757294 | 0.52965575 | 0.67564146 |
| Left lobules VIIt-VIIB | -0.1047439 | 0.39783633 | 0.56871183 |
| Left lobule VIII | 0.03720626 | 0.76810199 | 0.84243444 |
| Left lobule IX | 0.05225129 | 0.63921098 | 0.7399237 |
| Left lobule X | 0.01129064 | 0.92118754 | 0.93493661 |
| Vermis I-V | 0.07609257 | 0.54375721 | 0.67564146 |
| Vermis VI-VII | 0.00518324 | 0.96288199 | 0.96288199 |
| Vermis VIII-X | 0.10993762 | 0.37475711 | 0.5636609 |
| | | | |
| | **CBCL Externalizing** | | |
| | *Beta* | *p (uncorr.)* | *p (FDR)* |
| Right lobules I-V | -0.2150682 | 0.06996506 | 0.26474253 |
| Right lobule VI | -0.1969888 | 0.07652724 | 0.26474253 |
| Right lobule VIIf | -0.1787337 | 0.12511259 | 0.32645731 |
| Right lobules VIIt-VIIE | -0.3103051 | 0.00879746 | 0.07477841 |
| Right lobule VIII | -0.3364798 | 0.00559981 | 0.06151314 |
| Right lobule IX | -0.1900756 | 0.08120421 | 0.26474253 |
| Right lobule X | -0.1270135 | 0.27477651 | 0.51246692 |
| Left lobules I-V | -0.4292232 | 0.00062517 | 0.01417063 |
| Left lobule VI | -0.2108037 | 0.05367328 | 0.25898048 |
| Left lobule VIIf | -0.103931 | 0.37296175 | 0.5636609 |
| Left lobules VIIt-VIIB | -0.2932683 | 0.01446462 | 0.09835944 |
| Left lobule VIII | -0.4573253 | 0.00018819 | 0.00639848 |
| Left lobule IX | -0.1094581 | 0.31049771 | 0.54138063 |
| Left lobule X | -0.1437411 | 0.19331618 | 0.41079688 |
| Vermis I-V | -0.3837003 | 0.00156698 | 0.02663869 |
| Vermis VI-VII | -0.0650365 | 0.54645718 | 0.67564146 |
| Vermis VIII-X | -0.1829715 | 0.12710206 | 0.32645731 |
| | | | |
| | **PQ-BC** | | |
| | *Beta* | *p (uncorr.)* | *p (FDR)* |
| Right lobules I-V | -0.3990947 | 0.00633224 | 0.06151314 |
| Right lobule VI | -0.1611976 | 0.23811911 | 0.46263141 |
| Right lobule VIIf | -0.2326676 | 0.10389179 | 0.30715833 |
| Right lobules VIIt-VIIE | -0.1903022 | 0.19070384 | 0.41079688 |
| Right lobule VIII | -0.2606347 | 0.08175872 | 0.26474253 |
| Right lobule IX | -0.1422815 | 0.28740731 | 0.51430781 |
| Right lobule X | -0.1357668 | 0.34246971 | 0.5636609 |
| Left lobules I-V | -0.6029836 | 9.47E-05 | 0.00639848 |
| Left lobule VI | -0.1337009 | 0.32058567 | 0.54499564 |
| Left lobule VIIf | -0.3354765 | 0.0192175 | 0.11879911 |
| Left lobules VIIt-VIIB | -0.1288673 | 0.38108585 | 0.5636609 |
| Left lobule VIII | -0.2520116 | 0.09551254 | 0.29522058 |
| Left lobule IX | -0.04333 | 0.74340075 | 0.82870903 |
| Left lobule X | -0.1114403 | 0.41310694 | 0.57329126 |

| | | | |
|---|---|---|---|
| Vermis I-V | -0.2920142 | 0.05017968 | 0.25898048 |
| Vermis VI-VII | 0.1678435 | 0.20541554 | 0.42328051 |
| Vermis VIII-X | -0.1758617 | 0.23268375 | 0.46263141 |

## SUPPLEMENTAL TABLE 18. Effects of cortical volumes on measures of psychopathology

p-values in red reflect significance after FDR correction

All statistics shown are derived from two-sided linear mixed effects regressions

**N (CBCL models) = 10040**

**N (PQB model) = 10048**

**Included covariates**

Fixed effects: age, sex, ICV, Euler number (surface holes)

Random effects: site, scanner, family ID

**FDR** corrects for 68 regions x 4 scales = 272 comparisons

| | **CBCL Total** | | |
|---|---|---|---|
| **Region** | *Beta* | *p (uncorr.)* | *p (FDR)* |
| Left bankssts volume | -0.1549368 | 0.194194864 | 0.31071178 |
| Left caudalanteriorcingulate volume | -0.2086648 | 0.069295417 | 0.16386418 |
| Left caudalmiddlefrontal volume | -0.0896942 | 0.474924624 | 0.60933725 |
| Left cuneus volume | 0.0046167 | 0.969473251 | 0.97937586 |
| Left entorhinal volume | -0.1187474 | 0.300251246 | 0.43907709 |
| Left fusiform volume | -0.3547153 | 0.010111225 | 0.03667004 |
| Left inferiorparietal volume | -0.4751628 | 0.00020098 | 0.00260317 |
| Left inferiortemporal volume | -0.2925734 | 0.032855582 | 0.09407072 |
| Left isthmuscingulate volume | -0.1374723 | 0.292272185 | 0.4297191 |
| Left lateraloccipital volume | -0.1410331 | 0.305462031 | 0.44290326 |
| Left lateralorbitofrontal volume | -0.3085273 | 0.050317036 | 0.13550727 |
| Left lingual volume | -0.2326509 | 0.061347578 | 0.15594898 |
| Left medialorbitofrontal volume | -0.0778698 | 0.579444158 | 0.70965854 |
| Left middletemporal volume | -0.2299928 | 0.109580534 | 0.21756135 |
| Left parahippocampal volume | -0.125901 | 0.270075073 | 0.40570215 |
| Left paracentral volume | -0.322736 | 0.007738854 | 0.02923567 |
| Left parsopercularis volume | 0.00414257 | 0.972174571 | 0.97937586 |
| Left parsorbitalis volume | -0.2807101 | 0.024113363 | 0.07902211 |
| Left parstriangularis volume | -0.0987274 | 0.407306502 | 0.54042619 |
| Left pericalcarine volume | 0.04107366 | 0.731292679 | 0.81521151 |
| Left postcentral volume | -0.412728 | 0.002443421 | 0.01324897 |
| Left posteriorcingulate volume | -0.2048793 | 0.117066606 | 0.22404373 |
| Left precentral volume | -0.5551768 | 9.68E-05 | 0.00138589 |
| Left precuneus volume | -0.4441584 | 0.002220732 | 0.01265815 |
| Left rostralanteriorcingulate volume | -0.282147 | 0.032803829 | 0.09407072 |
| Left rostralmiddlefrontal volume | -0.513654 | 0.000302155 | 0.00328745 |
| Left superiorfrontal volume | -0.5440909 | 0.000266183 | 0.00324308 |
| Left superiorparietal volume | -0.2929796 | 0.024804331 | 0.08031879 |
| Left superiortemporal volume | -0.4953349 | 0.000612147 | 0.00504557 |

| | | | |
|---|---|---|---|
| Left supramarginal volume | -0.4173181 | 0.001266933 | 0.00861514 |
| Left frontalpole volume | -0.0656866 | 0.582318407 | 0.70965854 |
| Left temporalpole volume | -0.1608589 | 0.162329478 | 0.274661 |
| Left transversetemporal volume | -0.1050905 | 0.380294213 | 0.51358458 |
| Left insula volume | -0.6012703 | 2.22E-05 | 0.00050413 |
| Right bankssts volume | -0.2175044 | 0.083671078 | 0.17780104 |
| Right caudalanteriorcingulate volume | -0.322298 | 0.004502053 | 0.01943743 |
| Right caudalmiddlefrontal volume | -0.1817867 | 0.140015421 | 0.2489163 |
| Right cuneus volume | -0.0597752 | 0.62722104 | 0.74902069 |
| Right entorhinal volume | -0.1727647 | 0.135223526 | 0.24358145 |
| Right fusiform volume | -0.4611164 | 0.001663113 | 0.01052016 |
| Right inferiorparietal volume | -0.6432761 | 2.17E-06 | 0.0001182 |
| Right inferiortemporal volume | -0.4340516 | 0.00187807 | 0.01126963 |
| Right isthmuscingulate volume | 0.02978233 | 0.811953699 | 0.87566506 |
| Right lateraloccipital volume | -0.0916294 | 0.510901386 | 0.64039252 |
| Right lateralorbitofrontal volume | -0.4089977 | 0.006684186 | 0.02634926 |
| Right lingual volume | -0.0164378 | 0.894102886 | 0.93834288 |
| Right medialorbitofrontal volume | -0.1495626 | 0.271462465 | 0.40570215 |
| Right middletemporal volume | -0.5366416 | 0.00036565 | 0.00342954 |
| Right parahippocampal volume | 0.05503757 | 0.638134175 | 0.75466302 |
| Right paracentral volume | -0.3655239 | 0.002497286 | 0.01324897 |
| Right parsopercularis volume | 0.04887137 | 0.681698789 | 0.78903009 |
| Right parsorbitalis volume | -0.3166382 | 0.011117629 | 0.03927266 |
| Right parstriangularis volume | -0.042679 | 0.717148398 | 0.81033999 |
| Right pericalcarine volume | 0.01340752 | 0.911691035 | 0.9501148 |
| Right postcentral volume | -0.6244444 | 3.56E-06 | 0.00016116 |
| Right posteriorcingulate volume | -0.378019 | 0.003566721 | 0.01644319 |
| Right precentral volume | -0.6667726 | 8.74E-07 | 5.95E-05 |
| Right precuneus volume | -0.2768271 | 0.064648662 | 0.15985851 |
| Right rostralanteriorcingulate volume | -0.1139338 | 0.346061602 | 0.48771376 |
| Right rostralmiddlefrontal volume | -0.193581 | 0.154643706 | 0.26454772 |
| Right superiorfrontal volume | -0.379983 | 0.008862298 | 0.03277431 |
| Right superiorparietal volume | -0.2636614 | 0.044006783 | 0.12090753 |
| Right superiortemporal volume | -0.4493201 | 0.001905893 | 0.01126963 |
| Right supramarginal volume | -0.4821437 | 0.000178558 | 0.00242839 |
| Right frontalpole volume | -0.1692869 | 0.15087439 | 0.25973313 |
| Right temporalpole volume | -0.1049148 | 0.362670555 | 0.49571051 |
| Right transversetemporal volume | -0.211863 | 0.081243708 | 0.17678631 |
| Right insula volume | -0.4579403 | 0.001193503 | 0.00832392 |
| | | | |
| | **CBCL Internalizing** | | |
| | *Beta* | *p (uncorr.)* | *p (FDR)* |
| Left bankssts volume | -0.0955538 | 0.409500005 | 0.54069904 |
| Left caudalanteriorcingulate volume | -0.0679169 | 0.542837615 | 0.66810783 |
| Left caudalmiddlefrontal volume | -0.0436436 | 0.719639074 | 0.81033999 |

| | | | |
|---|---|---|---|
| Left cuneus volume | 0.15203441 | 0.18855259 | 0.30527562 |
| Left entorhinal volume | 0.00829728 | 0.940442582 | 0.96528446 |
| Left fusiform volume | -0.0727635 | 0.584424678 | 0.70965854 |
| Left inferiorparietal volume | -0.3355414 | 0.006519438 | 0.02607775 |
| Left inferiortemporal volume | -0.028701 | 0.82806148 | 0.88326558 |
| Left isthmuscingulate volume | 0.04404099 | 0.726355359 | 0.81303974 |
| Left lateraloccipital volume | 0.00173603 | 0.989478504 | 0.98959425 |
| Left lateralorbitofrontal volume | -0.0471288 | 0.756208195 | 0.83479628 |
| Left lingual volume | 0.01135317 | 0.924204092 | 0.95711916 |
| Left medialorbitofrontal volume | -0.0990624 | 0.460547026 | 0.59592083 |
| Left middletemporal volume | 0.06642917 | 0.630609327 | 0.74902069 |
| Left parahippocampal volume | 0.02994387 | 0.785897335 | 0.8550563 |
| Left paracentral volume | -0.3206883 | 0.006254732 | 0.02571803 |
| Left parsopercularis volume | 0.15137446 | 0.18771556 | 0.30527562 |
| Left parsorbitalis volume | -0.1588992 | 0.187223477 | 0.30527562 |
| Left parstriangularis volume | -0.0064797 | 0.955186652 | 0.97452866 |
| Left pericalcarine volume | 0.10043259 | 0.381412075 | 0.51358458 |
| Left postcentral volume | -0.377004 | 0.004092737 | 0.01795524 |
| Left posteriorcingulate volume | -0.1198493 | 0.343284768 | 0.48632009 |
| Left precentral volume | -0.2170158 | 0.113634059 | 0.22006972 |
| Left precuneus volume | -0.1293419 | 0.353671035 | 0.49021499 |
| Left rostralanteriorcingulate volume | -0.1952759 | 0.126627726 | 0.2333287 |
| Left rostralmiddlefrontal volume | -0.2962584 | 0.030916537 | 0.09151138 |
| Left superiorfrontal volume | -0.2820405 | 0.051229987 | 0.13575503 |
| Left superiorparietal volume | -0.1226231 | 0.328648884 | 0.47048682 |
| Left superiortemporal volume | -0.2517332 | 0.070654769 | 0.16386418 |
| Left supramarginal volume | -0.1922414 | 0.124938238 | 0.23276165 |
| Left frontalpole volume | 0.10702974 | 0.35456659 | 0.49021499 |
| Left temporalpole volume | -0.1309742 | 0.238602297 | 0.36874901 |
| Left transversetemporal volume | -0.0103137 | 0.928968597 | 0.95711916 |
| Left insula volume | -0.3566322 | 0.008916541 | 0.03277431 |
| Right bankssts volume | -0.1412645 | 0.245581247 | 0.37527022 |
| Right caudalanteriorcingulate volume | -0.1722045 | 0.117787698 | 0.22404373 |
| Right caudalmiddlefrontal volume | -0.1328642 | 0.265539082 | 0.40350073 |
| Right cuneus volume | 0.10254556 | 0.3868477 | 0.51712694 |
| Right entorhinal volume | -0.0823954 | 0.462276817 | 0.59592083 |
| Right fusiform volume | -0.088535 | 0.531859141 | 0.66057391 |
| Right inferiorparietal volume | -0.2535358 | 0.052662755 | 0.13773336 |
| Right inferiortemporal volume | -0.1778791 | 0.185527878 | 0.30527562 |
| Right isthmuscingulate volume | 0.17808051 | 0.139412154 | 0.2489163 |
| Right lateraloccipital volume | 0.12210977 | 0.360165479 | 0.49477278 |
| Right lateralorbitofrontal volume | -0.2489327 | 0.085878216 | 0.17831202 |
| Right lingual volume | 0.02474438 | 0.835140088 | 0.88733634 |
| Right medialorbitofrontal volume | -0.0552129 | 0.673295586 | 0.78599313 |
| Right middletemporal volume | -0.1477287 | 0.306124309 | 0.44290326 |

| | | | |
|---|---|---|---|
| Right parahippocampal volume | 0.14334671 | 0.205323044 | 0.32469691 |
| Right paracentral volume | -0.3191883 | 0.006334955 | 0.02571803 |
| Right parsopercularis volume | 0.04799152 | 0.677467398 | 0.78748347 |
| Right parsorbitalis volume | -0.158174 | 0.189737758 | 0.30537675 |
| Right parstriangularis volume | -0.0277815 | 0.807816785 | 0.87540305 |
| Right pericalcarine volume | 0.10802946 | 0.352171038 | 0.49021499 |
| Right postcentral volume | -0.3786162 | 0.003555813 | 0.01644319 |
| Right posteriorcingulate volume | -0.2293319 | 0.066908983 | 0.16230226 |
| Right precentral volume | -0.3324105 | 0.011008796 | 0.03927266 |
| Right precuneus volume | -0.0503845 | 0.720964254 | 0.81033999 |
| Right rostralanteriorcingulate volume | 0.02747685 | 0.814497277 | 0.87566506 |
| Right rostralmiddlefrontal volume | -0.0392462 | 0.765389444 | 0.83608807 |
| Right superiorfrontal volume | -0.2468254 | 0.077838846 | 0.17213143 |
| Right superiorparietal volume | -0.1015178 | 0.419868027 | 0.55171064 |
| Right superiortemporal volume | -0.2573912 | 0.063913776 | 0.15949126 |
| Right supramarginal volume | -0.2518009 | 0.042535774 | 0.11805847 |
| Right frontalpole volume | 0.01607392 | 0.891361513 | 0.93834288 |
| Right temporalpole volume | -0.0887632 | 0.426378312 | 0.55757164 |
| Right transversetemporal volume | -0.1783292 | 0.127816088 | 0.2333287 |
| Right insula volume | -0.2293146 | 0.091983808 | 0.18811726 |
| | | | |
| | **CBCL Externalizing** | | |
| | *Beta* | *p (uncorr.)* | *p (FDR)* |
| Left bankssts volume | -0.2992444 | 0.007733168 | 0.02923567 |
| Left caudalanteriorcingulate volume | -0.1510809 | 0.162575077 | 0.274661 |
| Left caudalmiddlefrontal volume | -0.092416 | 0.43392299 | 0.56472274 |
| Left cuneus volume | -0.0507578 | 0.651324856 | 0.76386844 |
| Left entorhinal volume | -0.1808075 | 0.092974346 | 0.18872405 |
| Left fusiform volume | -0.46923 | 0.000285462 | 0.00324308 |
| Left inferiorparietal volume | -0.4691662 | 8.99E-05 | 0.00138589 |
| Left inferiortemporal volume | -0.4212931 | 0.00105665 | 0.00776781 |
| Left isthmuscingulate volume | -0.1691753 | 0.166096508 | 0.27887809 |
| Left lateraloccipital volume | -0.2028055 | 0.114080262 | 0.22006972 |
| Left lateralorbitofrontal volume | -0.3973134 | 0.006956283 | 0.02703013 |
| Left lingual volume | -0.3277803 | 0.004700404 | 0.01997672 |
| Left medialorbitofrontal volume | -0.0408219 | 0.758068678 | 0.83479628 |
| Left middletemporal volume | -0.46759 | 0.000501905 | 0.00426619 |
| Left parahippocampal volume | -0.1935597 | 0.070556462 | 0.16386418 |
| Left paracentral volume | -0.252685 | 0.026512165 | 0.08194669 |
| Left parsopercularis volume | -0.1361335 | 0.222022714 | 0.34508673 |
| Left parsorbitalis volume | -0.2473864 | 0.034457204 | 0.09762875 |
| Left parstriangularis volume | -0.1200294 | 0.283183015 | 0.41861837 |
| Left pericalcarine volume | -0.010346 | 0.925887163 | 0.95711916 |
| Left postcentral volume | -0.304142 | 0.017299587 | 0.05809244 |
| Left posteriorcingulate volume | -0.2648173 | 0.03095238 | 0.09151138 |

| | | | |
|---|---|---|---|
| Left precentral volume | -0.5569865 | 2.94E-05 | 0.00061417 |
| Left precuneus volume | -0.4571438 | 0.000744886 | 0.00578883 |
| Left rostralanteriorcingulate volume | -0.2661719 | 0.031988072 | 0.09355651 |
| Left rostralmiddlefrontal volume | -0.4756784 | 0.00035729 | 0.00342954 |
| Left superiorfrontal volume | -0.5774596 | 4.00E-05 | 0.00077766 |
| Left superiorparietal volume | -0.227662 | 0.062122846 | 0.15645754 |
| Left superiortemporal volume | -0.6159472 | 5.22E-06 | 0.00018347 |
| Left supramarginal volume | -0.5275948 | 1.44E-05 | 0.00035681 |
| Left frontalpole volume | -0.137715 | 0.220818415 | 0.34508673 |
| Left temporalpole volume | -0.2408687 | 0.025695121 | 0.08126829 |
| Left transversetemporal volume | -0.1234851 | 0.271018379 | 0.40570215 |
| Left insula volume | -0.5801134 | 1.19E-05 | 0.00034017 |
| Right bankssts volume | -0.3717667 | 0.001629621 | 0.01052016 |
| Right caudalanteriorcingulate volume | -0.3609158 | 0.0007247 | 0.00578883 |
| Right caudalmiddlefrontal volume | -0.2061051 | 0.075222619 | 0.16909547 |
| Right cuneus volume | -0.0739427 | 0.51964787 | 0.64836798 |
| Right entorhinal volume | -0.1804519 | 0.096521068 | 0.19304214 |
| Right fusiform volume | -0.4177016 | 0.002337502 | 0.01297552 |
| Right inferiorparietal volume | -0.7220688 | 1.33E-08 | 1.81E-06 |
| Right inferiortemporal volume | -0.4733828 | 0.000286154 | 0.00324308 |
| Right isthmuscingulate volume | -0.043055 | 0.713239304 | 0.81033999 |
| Right lateraloccipital volume | -0.2244903 | 0.083599203 | 0.17780104 |
| Right lateralorbitofrontal volume | -0.421229 | 0.002943611 | 0.01431741 |
| Right lingual volume | -0.0592795 | 0.607566517 | 0.7312305 |
| Right medialorbitofrontal volume | -0.23224 | 0.068694604 | 0.16386418 |
| Right middletemporal volume | -0.8136906 | 7.02E-09 | 1.81E-06 |
| Right parahippocampal volume | -0.0415684 | 0.704888868 | 0.81033999 |
| Right paracentral volume | -0.2835463 | 0.012637605 | 0.0440696 |
| Right parsopercularis volume | -0.0540118 | 0.629395214 | 0.74902069 |
| Right parsorbitalis volume | -0.2140231 | 0.067427041 | 0.16230226 |
| Right parstriangularis volume | 0.00552836 | 0.960197356 | 0.97452866 |
| Right pericalcarine volume | -0.0014677 | 0.989594251 | 0.98959425 |
| Right postcentral volume | -0.5013115 | 7.29E-05 | 0.00123899 |
| Right posteriorcingulate volume | -0.3952141 | 0.001178577 | 0.00832392 |
| Right precentral volume | -0.6634691 | 1.77E-07 | 1.60E-05 |
| Right precuneus volume | -0.2476353 | 0.071690579 | 0.16386418 |
| Right rostralanteriorcingulate volume | -0.1959219 | 0.085146767 | 0.17815324 |
| Right rostralmiddlefrontal volume | -0.2487417 | 0.051407235 | 0.13575503 |
| Right superiorfrontal volume | -0.4248894 | 0.001805305 | 0.01116007 |
| Right superiorparietal volume | -0.2042172 | 0.09541044 | 0.19223437 |
| Right superiortemporal volume | -0.4826758 | 0.000361741 | 0.00342954 |
| Right supramarginal volume | -0.4709747 | 9.58E-05 | 0.00138589 |
| Right frontalpole volume | -0.1990832 | 0.082399144 | 0.17780104 |
| Right temporalpole volume | -0.1573023 | 0.145962311 | 0.25614031 |
| Right transversetemporal volume | -0.1940361 | 0.08835202 | 0.18205871 |

| | | | |
|---|---|---|---|
| Right insula volume | -0.4721122 | 0.000356846 | 0.00342954 |
| | | | |
| | **PQ-BC** | | |
| | *Beta* | *p (uncorr.)* | *p (FDR)* |
| Left bankssts volume | -0.0210862 | 0.880949035 | 0.93236629 |
| Left caudalanteriorcingulate volume | -0.1963464 | 0.147035173 | 0.25636902 |
| Left caudalmiddlefrontal volume | -0.0496153 | 0.736661201 | 0.81784427 |
| Left cuneus volume | -0.284874 | 0.039803217 | 0.11161315 |
| Left entorhinal volume | -0.2932342 | 0.028838789 | 0.08813652 |
| Left fusiform volume | -0.4850007 | 0.00260359 | 0.01336182 |
| Left inferiorparietal volume | -0.0545891 | 0.714100261 | 0.81033999 |
| Left inferiortemporal volume | -0.4757637 | 0.002947702 | 0.01431741 |
| Left isthmuscingulate volume | -0.2309764 | 0.127782944 | 0.2333287 |
| Left lateraloccipital volume | -0.3713627 | 0.019633554 | 0.06512594 |
| Left lateralorbitofrontal volume | -0.829063 | 5.40E-06 | 0.00018347 |
| Left lingual volume | -0.2585496 | 0.071089442 | 0.16386418 |
| Left medialorbitofrontal volume | -0.261022 | 0.11270421 | 0.22006972 |
| Left middletemporal volume | -0.4571422 | 0.006043245 | 0.02528865 |
| Left parahippocampal volume | -0.2978342 | 0.02512102 | 0.08038727 |
| Left paracentral volume | -0.4726583 | 0.000874707 | 0.0066089 |
| Left parsopercularis volume | -0.0627628 | 0.651534847 | 0.76386844 |
| Left parsorbitalis volume | -0.2554812 | 0.079972632 | 0.17542384 |
| Left parstriangularis volume | 0.09369554 | 0.501499747 | 0.6315182 |
| Left pericalcarine volume | -0.243228 | 0.075970883 | 0.16937771 |
| Left postcentral volume | -0.3533291 | 0.026185975 | 0.0818688 |
| Left posteriorcingulate volume | -0.2427036 | 0.11255022 | 0.22006972 |
| Left precentral volume | -0.6615811 | 6.37E-05 | 0.00115501 |
| Left precuneus volume | -0.3069721 | 0.067069222 | 0.16230226 |
| Left rostralanteriorcingulate volume | -0.4532399 | 0.003372106 | 0.01609145 |
| Left rostralmiddlefrontal volume | -0.1534482 | 0.355045419 | 0.49021499 |
| Left superiorfrontal volume | -0.1699729 | 0.330782632 | 0.47106218 |
| Left superiorparietal volume | -0.2851591 | 0.059309457 | 0.15219031 |
| Left superiortemporal volume | -0.0507021 | 0.762673286 | 0.83608807 |
| Left supramarginal volume | -0.2715891 | 0.073929749 | 0.1675741 |
| Left frontalpole volume | -0.2068212 | 0.141943178 | 0.25070483 |
| Left temporalpole volume | -0.180907 | 0.178253722 | 0.29745406 |
| Left transversetemporal volume | -0.1414856 | 0.310155186 | 0.4463609 |
| Left insula volume | -0.4060734 | 0.013452372 | 0.04631703 |
| Right bankssts volume | -0.1328277 | 0.367171435 | 0.49935315 |
| Right caudalanteriorcingulate volume | 0.09066228 | 0.497134526 | 0.62893298 |
| Right caudalmiddlefrontal volume | 0.0990595 | 0.493715083 | 0.62752571 |
| Right cuneus volume | -0.2444898 | 0.085080218 | 0.17815324 |
| Right entorhinal volume | -0.2092256 | 0.122188534 | 0.22920884 |
| Right fusiform volume | -0.7442942 | 1.25E-05 | 0.00034017 |
| Right inferiorparietal volume | -0.3433921 | 0.029651153 | 0.08961237 |

| | | | |
|---|---|---|---|
| Right inferiortemporal volume | -0.5658917 | 0.000465067 | 0.00421661 |
| Right isthmuscingulate volume | 0.03224322 | 0.824730842 | 0.88317634 |
| Right lateraloccipital volume | -0.5096062 | 0.001501043 | 0.00995814 |
| Right lateralorbitofrontal volume | -0.6128962 | 0.000485851 | 0.00426295 |
| Right lingual volume | -0.2055574 | 0.150683361 | 0.25973313 |
| Right medialorbitofrontal volume | -0.3032857 | 0.056177995 | 0.14552776 |
| Right middletemporal volume | -0.5217058 | 0.002701958 | 0.01360986 |
| Right parahippocampal volume | -0.084275 | 0.536687503 | 0.66354091 |
| Right paracentral volume | -0.1741749 | 0.219518094 | 0.34508673 |
| Right parsopercularis volume | -0.1853835 | 0.184466498 | 0.30527562 |
| Right parsorbitalis volume | -0.1569014 | 0.28181369 | 0.41861837 |
| Right parstriangularis volume | 0.16266766 | 0.24003261 | 0.36886367 |
| Right pericalcarine volume | -0.1198321 | 0.387845202 | 0.51712694 |
| Right postcentral volume | -0.4530097 | 0.003940075 | 0.01786167 |
| Right posteriorcingulate volume | 0.05550641 | 0.714240859 | 0.81033999 |
| Right precentral volume | -0.47703 | 0.002532891 | 0.01324897 |
| Right precuneus volume | -0.3372464 | 0.047322299 | 0.12871665 |
| Right rostralanteriorcingulate volume | -0.10115 | 0.477277736 | 0.60948143 |
| Right rostralmiddlefrontal volume | 0.00836271 | 0.958115373 | 0.97452866 |
| Right superiorfrontal volume | -0.2559764 | 0.130666062 | 0.23694113 |
| Right superiorparietal volume | -0.464001 | 0.002233792 | 0.01265815 |
| Right superiortemporal volume | -0.2125567 | 0.204579678 | 0.32469691 |
| Right supramarginal volume | -0.2346152 | 0.118698559 | 0.22420839 |
| Right frontalpole volume | -0.3484219 | 0.01470405 | 0.04999377 |
| Right temporalpole volume | -0.0727162 | 0.589790647 | 0.71299136 |
| Right transversetemporal volume | -0.0183084 | 0.8969454 | 0.93834288 |
| Right insula volume | -0.4720253 | 0.004045538 | 0.01795524 |

**SUPPLEMENTAL TABLE 19. Effects of subcortical volumes on measures of psychopathology**

p-values in red reflect significance after FDR correction

All statistics shown are derived from two-sided linear mixed effects regressions

**N (CBCL models) = 10040**

**N (PQB model) = 10048**

**Included covariates**

Fixed effects: age, sex, ICV, Euler number (surface holes)

Random effects: site, scanner, family ID

**FDR** corrects for 17 regions x 4 scales = 68 comparisons

| | **CBCL Total** | | |
|---|---|---|---|
| **Region** | *Beta* | *p (uncorr.)* | *p (FDR)* |
| Brain Stem | -0.4666768 | 0.00254085 | 0.02468254 |
| Left Thalamus | -0.3878639 | 0.013123154 | 0.06864419 |
| Left Caudate | -0.3227715 | 0.017428423 | 0.06971369 |
| Left Putamen | -0.1329765 | 0.311936085 | 0.48208304 |
| Left Pallidum | -0.3410716 | 0.015660465 | 0.06971369 |
| Left Hippocampus | -0.3941415 | 0.007063964 | 0.04803495 |
| Left Amygdala | -0.1348189 | 0.320201082 | 0.48385941 |
| Left Accumbens area | -0.2736038 | 0.061448488 | 0.15878331 |
| Left VentralDC | -0.1089143 | 0.466841276 | 0.54281145 |
| Right Thalamus | -0.3972678 | 0.008899938 | 0.05043298 |
| Right Caudate | -0.2672247 | 0.048855399 | 0.14444205 |
| Right Putamen | -0.1362908 | 0.297858074 | 0.47103137 |
| Right Pallidum | -0.2621734 | 0.047196032 | 0.14444205 |
| Right Hippocampus | -0.3391012 | 0.017238408 | 0.06971369 |
| Right Amygdala | -0.1709804 | 0.221058174 | 0.39103081 |
| Right Accumbens area | -0.3538428 | 0.008272999 | 0.05043298 |
| Right VentralDC | -0.0347427 | 0.815562219 | 0.86000906 |
| | | | |
| | **CBCL Internalizing** | | |
| **Region** | *Beta* | *p (uncorr.)* | *p (FDR)* |
| Brain Stem | -0.1429046 | 0.333207615 | 0.49256778 |
| Left Thalamus | -0.0926081 | 0.537316391 | 0.60895858 |
| Left Caudate | -0.2022093 | 0.119409805 | 0.26926605 |
| Left Putamen | 0.00366378 | 0.976738605 | 0.9767386 |
| Left Pallidum | -0.170655 | 0.20921359 | 0.39103081 |
| Left Hippocampus | -0.0645117 | 0.645488645 | 0.6967179 |
| Left Amygdala | -0.1198164 | 0.358404717 | 0.50774002 |
| Left Accumbens area | -0.1005802 | 0.470515227 | 0.54281145 |
| Left VentralDC | 0.1121401 | 0.434639704 | 0.54281145 |

| | | | |
|---|---|---|---|
| Right Thalamus | -0.1048927 | 0.470968757 | 0.54281145 |
| Right Caudate | -0.1691226 | 0.192018654 | 0.38403731 |
| Right Putamen | -0.0281534 | 0.822067481 | 0.86000906 |
| Right Pallidum | -0.1548379 | 0.223818504 | 0.39103081 |
| Right Hippocampus | -0.0139702 | 0.918478876 | 0.93218752 |
| Right Amygdala | -0.0970922 | 0.469244791 | 0.54281145 |
| Right Accumbens area | -0.2712061 | 0.034434197 | 0.11150121 |
| Right VentralDC | 0.22043596 | 0.122753643 | 0.26926605 |
| | | | |
| | **CBCL Externalizing** | | |
| **Region** | *Beta* | *p (uncorr.)* | *p (FDR)* |
| Brain Stem | -0.4047505 | 0.004750882 | 0.03589555 |
| Left Thalamus | -0.4423758 | 0.002427436 | 0.02468254 |
| Left Caudate | -0.2178246 | 0.083299963 | 0.20229991 |
| Left Putamen | -0.088729 | 0.467495141 | 0.54281145 |
| Left Pallidum | -0.2053126 | 0.118875213 | 0.26926605 |
| Left Hippocampus | -0.5751298 | 2.40E-05 | 0.00163008 |
| Left Amygdala | -0.1516024 | 0.232987034 | 0.39607796 |
| Left Accumbens area | -0.310942 | 0.024258321 | 0.08681926 |
| Left VentralDC | -0.260724 | 0.062031539 | 0.15878331 |
| Right Thalamus | -0.5037797 | 0.000377511 | 0.00855692 |
| Right Caudate | -0.1169378 | 0.352110619 | 0.50774002 |
| Right Putamen | -0.0657519 | 0.588787321 | 0.64576674 |
| Right Pallidum | -0.1036087 | 0.401009597 | 0.54281145 |
| Right Hippocampus | -0.4931781 | 0.000197824 | 0.00672601 |
| Right Amygdala | -0.1932476 | 0.139545846 | 0.29653492 |
| Right Accumbens area | -0.3690252 | 0.003314568 | 0.02817383 |
| Right VentralDC | -0.1114598 | 0.422142945 | 0.54281145 |
| | | | |
| | **PQ-BC** | | |
| **Region** | *Beta* | *p (uncorr.)* | *p (FDR)* |
| Brain Stem | -0.1274179 | 0.468846132 | 0.54281145 |
| Left Thalamus | -0.1459593 | 0.418647453 | 0.54281145 |
| Left Caudate | -0.5137114 | 0.000859124 | 0.01435594 |
| Left Putamen | -0.3628301 | 0.015495995 | 0.06971369 |
| Left Pallidum | -0.1916198 | 0.239715153 | 0.39686928 |
| Left Hippocampus | -0.214622 | 0.201314474 | 0.39103081 |
| Left Amygdala | -0.3714402 | 0.018681601 | 0.07057494 |
| Left Accumbens area | 0.32595864 | 0.058975313 | 0.15878331 |
| Left VentralDC | 0.03402766 | 0.84383755 | 0.86940838 |
| Right Thalamus | -0.1341711 | 0.44369869 | 0.54281145 |
| Right Caudate | -0.5040204 | 0.001055584 | 0.01435594 |
| Right Putamen | -0.3333102 | 0.025607929 | 0.08706696 |
| Right Pallidum | -0.1857299 | 0.224267669 | 0.39103081 |
| Right Hippocampus | -0.2179449 | 0.182421005 | 0.37589783 |

| | | | |
|---|---|---|---|
| Right Amygdala | -0.3015989 | 0.063046314 | 0.15878331 |
| Right Accumbens area | -0.1819507 | 0.245125142 | 0.39686928 |
| Right VentralDC | 0.09420436 | 0.582713798 | 0.64576674 |

**SUPPLEMENTAL TABLE 20. Effects of brain volume measures on measures of psychopathology in a subset of the highest quality scans**

p-values in red reflect significance after FDR correction

All statistics shown are derived from two-sided linear mixed effects regressions

*Global volumes*

**N (CBCL models) = 8658**

**N (PQB model) = 8666**

**FDR** corrects for 4 regions x 4 scales = 16 comparisons

*Cerebellar volumes*

**N (CBCL models) = 8658**

**N (PQB model) = 8666**

**FDR** corrects for 17 regions x 4 scales = 68 comparisons

*Cortical volumes*

**N (CBCL models) = 8658**

**N (PQB model) = 8666**

**FDR** corrects for 68 regions x 4 scales = 272 comparisons

*Subcortical volumes*

**N (CBCL models) = 8658**

**N (PQB model) = 8666**

**FDR** corrects for 17 regions x 4 scales = 68 comparisons

**Included covariates**

Fixed effects: age, sex, ICV, Euler number (surface holes)

Random effects: site, scanner, family ID

| | **CBCL Total** | | |
|---|---|---|---|
| ***Global volumes*** | | | |
| | | | |
| **Region** | *Beta* | *p (uncorr.)* | *p (FDR)* |
| Total Brain Gray Volume | -1.517049 | 1.78E-07 | 7.11E-07 |
| Cerebellum Total Gray Volume | -0.2557611 | 0.12585643 | 0.14383592 |
| Cortical Gray Volume | -1.2824539 | 1.07E-06 | 3.42E-06 |
| Subcortical Gray Volume | -0.5757376 | 0.00359617 | 0.00639318 |
| | | | |
| ***Cerebellar volumes*** | | | |
| | | | |
| **Region** | *Beta* | *p (uncorr.)* | *p (FDR)* |
| Right lobules I-V | 0.02441256 | 0.85887874 | 0.8985193 |

| | | | |
|---|---|---|---|
| Right lobule VI | 0.12866247 | 0.31990612 | 0.57915965 |
| Right lobule VIIf | -0.1293673 | 0.33923028 | 0.57915965 |
| Right lobules VIIt-VIIB | -0.3841116 | 0.00519324 | 0.08828506 |
| Right lobule VIII | -0.0252786 | 0.85778803 | 0.8985193 |
| Right lobule IX | -0.050321 | 0.6916125 | 0.84980783 |
| Right lobule X | -0.0927314 | 0.4920328 | 0.72735284 |
| Left lobules I-V | -0.2211138 | 0.12845428 | 0.40205346 |
| Left lobule VI | 0.17991048 | 0.15658548 | 0.40953127 |
| Left lobule VIIf | -0.0902247 | 0.5049593 | 0.73057941 |
| Left lobules VIIt-VIIB | -0.2792825 | 0.04467235 | 0.21559775 |
| Left lobule VIII | -0.2095676 | 0.13980427 | 0.40205346 |
| Left lobule IX | -0.028014 | 0.82300796 | 0.8985193 |
| Left lobule X | -0.0588264 | 0.64548462 | 0.84409527 |
| Vermis I-V | -0.2786206 | 0.04755833 | 0.21559775 |
| Vermis VI-VII | -0.1269213 | 0.31159864 | 0.57915965 |
| Vermis VIII-X | -0.1799246 | 0.19697646 | 0.46495215 |
| | | | |
| ***Cortical analyses*** | | | |
| | | | |
| **Region** | *Beta* | *p (uncorr.)* | *p (FDR)* |
| Left bankssts volume | -0.1132155 | 0.38165576 | 0.57038663 |
| Left caudalanteriorcingulate volume | -0.1624023 | 0.18786005 | 0.35984461 |
| Left caudalmiddlefrontal volume | -0.1244182 | 0.36341318 | 0.56144525 |
| Left cuneus volume | 0.02460864 | 0.84995918 | 0.90308163 |
| Left entorhinal volume | -0.0305981 | 0.80586231 | 0.87677819 |
| Left fusiform volume | -0.2179854 | 0.14728343 | 0.31656705 |
| Left inferiorparietal volume | -0.409923 | 0.00307002 | 0.0225688 |
| Left inferiortemporal volume | -0.2069845 | 0.16413835 | 0.33070839 |
| Left isthmuscingulate volume | -0.0927515 | 0.51527213 | 0.69383178 |
| Left lateraloccipital volume | -0.046914 | 0.75248245 | 0.83545367 |
| Left lateralorbitofrontal volume | -0.018976 | 0.91275501 | 0.93181306 |
| Left lingual volume | -0.178163 | 0.18500766 | 0.35944345 |
| Left medialorbitofrontal volume | 0.17347929 | 0.25990933 | 0.4454199 |
| Left middletemporal volume | -0.176143 | 0.26191167 | 0.4454199 |
| Left parahippocampal volume | -0.0526209 | 0.67082436 | 0.80664904 |
| Left paracentral volume | -0.3181266 | 0.01537837 | 0.0708969 |
| Left parsopercularis volume | 0.14228162 | 0.2710771 | 0.45234951 |
| Left parsorbitalis volume | -0.1092183 | 0.41844834 | 0.60820061 |
| Left parstriangularis volume | 0.00115913 | 0.99279616 | 0.99279616 |
| Left pericalcarine volume | 0.05116956 | 0.69147582 | 0.81267537 |
| Left postcentral volume | -0.4302922 | 0.00373713 | 0.02541249 |
| Left posteriorcingulate volume | -0.2036232 | 0.14936885 | 0.31656705 |
| Left precentral volume | -0.5049323 | 0.0012684 | 0.01380023 |
| Left precuneus volume | -0.3572191 | 0.0249438 | 0.09555933 |
| Left rostralanteriorcingulate volume | -0.2505232 | 0.07974821 | 0.21691514 |

| | | | |
|---|---|---|---|
| Left rostralmiddlefrontal volume | -0.3686506 | 0.01771997 | 0.07588339 |
| Left superiorfrontal volume | -0.4936526 | 0.00267217 | 0.02074618 |
| Left superiorparietal volume | -0.3290831 | 0.02138986 | 0.08683647 |
| Left superiortemporal volume | -0.4416836 | 0.00509018 | 0.03146654 |
| Left supramarginal volume | -0.3999071 | 0.00429304 | 0.02848066 |
| Left frontalpole volume | 0.12494171 | 0.34314654 | 0.53951363 |
| Left temporalpole volume | -0.0773534 | 0.54306871 | 0.7170616 |
| Left transversetemporal volume | -0.0468681 | 0.71649068 | 0.81542035 |
| Left insula volume | -0.4446306 | 0.00360614 | 0.02515049 |
| Right bankssts volume | -0.2013411 | 0.14502829 | 0.31558156 |
| Right caudalanteriorcingulate volume | -0.3587916 | 0.00347195 | 0.02485187 |
| Right caudalmiddlefrontal volume | -0.1526001 | 0.25504236 | 0.44468924 |
| Right cuneus volume | -0.0120419 | 0.92736529 | 0.93770765 |
| Right entorhinal volume | -0.1409798 | 0.26124134 | 0.4454199 |
| Right fusiform volume | -0.3777091 | 0.01762262 | 0.07588339 |
| Right inferiorparietal volume | -0.5456708 | 0.00023657 | 0.00459631 |
| Right inferiortemporal volume | -0.3377525 | 0.02591996 | 0.09657847 |
| Right isthmuscingulate volume | 0.04344871 | 0.75057485 | 0.83545367 |
| Right lateraloccipital volume | -0.0566635 | 0.70771948 | 0.81267537 |
| Right lateralorbitofrontal volume | -0.2988029 | 0.0703522 | 0.19727627 |
| Right lingual volume | 0.05427011 | 0.68373667 | 0.80859294 |
| Right medialorbitofrontal volume | -0.1807129 | 0.22512358 | 0.40725634 |
| Right middletemporal volume | -0.4151041 | 0.01171278 | 0.05868709 |
| Right parahippocampal volume | 0.09626846 | 0.44907542 | 0.62640264 |
| Right paracentral volume | -0.3371549 | 0.01033548 | 0.05406251 |
| Right parsopercularis volume | 0.10370621 | 0.42057515 | 0.60820061 |
| Right parsorbitalis volume | -0.1711623 | 0.20372257 | 0.37695605 |
| Right parstriangularis volume | -0.072601 | 0.57033737 | 0.72466325 |
| Right pericalcarine volume | 0.04066947 | 0.75559413 | 0.83545367 |
| Right postcentral volume | -0.5557638 | 0.00015372 | 0.00408187 |
| Right posteriorcingulate volume | -0.4668521 | 0.00085918 | 0.01016072 |
| Right precentral volume | -0.5346624 | 0.00034623 | 0.00523188 |
| Right precuneus volume | -0.1447425 | 0.36948051 | 0.56144525 |
| Right rostralanteriorcingulate volume | -0.1006363 | 0.43841975 | 0.62187747 |
| Right rostralmiddlefrontal volume | -0.1575969 | 0.2873254 | 0.4679791 |
| Right superiorfrontal volume | -0.2725748 | 0.08618807 | 0.23211046 |
| Right superiorparietal volume | -0.1923395 | 0.17867163 | 0.35216437 |
| Right superiortemporal volume | -0.4939961 | 0.00183137 | 0.01623653 |
| Right supramarginal volume | -0.5373024 | 0.00011863 | 0.00408187 |
| Right frontalpole volume | -0.1938629 | 0.15013658 | 0.31656705 |
| Right temporalpole volume | -0.0467399 | 0.71186893 | 0.81356449 |
| Right transversetemporal volume | -0.1727694 | 0.18742491 | 0.35984461 |
| Right insula volume | -0.3842618 | 0.01208264 | 0.05868709 |
| | | | |
| ***Subcortical analyses*** | | | |

| | | | |
|---|---|---|---|
| **Region** | *Beta* | *p (uncorr.)* | *p (FDR)* |
| Brain Stem | -0.4255996 | 0.0114789 | 0.07271651 |
| Left Thalamus | -0.4143221 | 0.0160565 | 0.0779887 |
| Left Caudate | -0.2814682 | 0.05554601 | 0.17354739 |
| Left Putamen | -0.1926794 | 0.17460182 | 0.29650839 |
| Left Pallidum | -0.3743111 | 0.01432536 | 0.07493263 |
| Left Hippocampus | -0.3043976 | 0.05614768 | 0.17354739 |
| Left Amygdala | -0.1984133 | 0.17267913 | 0.29650839 |
| Left Accumbens area | -0.1627099 | 0.30805382 | 0.42750326 |
| Left VentralDC | 0.01220714 | 0.9401509 | 0.954183 |
| Right Thalamus | -0.4911497 | 0.00316449 | 0.03586419 |
| Right Caudate | -0.2587266 | 0.07825769 | 0.21286092 |
| Right Putamen | -0.209199 | 0.13812105 | 0.29650839 |
| Right Pallidum | -0.2822587 | 0.04699543 | 0.16944973 |
| Right Hippocampus | -0.2778372 | 0.0713573 | 0.20217902 |
| Right Amygdala | -0.2018193 | 0.17981314 | 0.29650839 |
| Right Accumbens area | -0.3215381 | 0.02517169 | 0.10697967 |
| Right VentralDC | 0.04176793 | 0.79523678 | 0.83194002 |

| | **CBCL Internalizing** | | |
|---|---|---|---|
| ***Global volumes*** | | | |
| | | | |
| **Region** | *Beta* | *p (uncorr.)* | *p (FDR)* |
| Total Brain Gray Volume | -0.4995046 | 0.07118776 | 0.0876157 |
| Cerebellum Total Gray Volume | 0.1243072 | 0.43727641 | 0.43727641 |
| Cortical Gray Volume | -0.5067873 | 0.04350674 | 0.05800898 |
| Subcortical Gray Volume | -0.2247498 | 0.23533791 | 0.2510271 |
| | | | |
| ***Cerebellar volumes*** | | | |
| | | | |
| **Region** | *Beta* | *p (uncorr.)* | *p (FDR)* |
| Right lobules I-V | 0.19175759 | 0.14644635 | 0.40205346 |
| Right lobule VI | 0.17962785 | 0.14781377 | 0.40205346 |
| Right lobule VIIf | -0.0021982 | 0.98651923 | 0.9939587 |
| Right lobules VIIt-VIIB | -0.1615348 | 0.22107434 | 0.50110184 |
| Right lobule VIII | 0.24978166 | 0.0651996 | 0.2770983 |
| Right lobule IX | 0.11598896 | 0.34068215 | 0.57915965 |
| Right lobule X | 0.02697403 | 0.83522979 | 0.8985193 |
| Left lobules I-V | 0.09821335 | 0.48260764 | 0.72735284 |
| Left lobule VI | 0.25396875 | 0.0375309 | 0.21559775 |
| Left lobule VIIf | -0.0282967 | 0.82776244 | 0.8985193 |
| Left lobules VIIt-VIIB | -0.1136503 | 0.3949048 | 0.62450062 |
| Left lobule VIII | 0.1576021 | 0.24807292 | 0.52715495 |
| Left lobule IX | 0.10838288 | 0.36703565 | 0.60874205 |

| | | | |
|---|---|---|---|
| Left lobule X | 0.02678464 | 0.82777117 | 0.8985193 |
| Vermis I-V | 0.05304439 | 0.69480718 | 0.84980783 |
| Vermis VI-VII | -0.0323974 | 0.78792712 | 0.8985193 |
| Vermis VIII-X | -0.0010145 | 0.9939587 | 0.9939587 |
| | | | |
| ***Cortical analyses*** | | | |
| | | | |
| **Region** | *Beta* | *p (uncorr.)* | *p (FDR)* |
| Left bankssts volume | -0.0534603 | 0.67097759 | 0.80664904 |
| Left caudalanteriorcingulate volume | -0.0617587 | 0.60681566 | 0.75367059 |
| Left caudalmiddlefrontal volume | -0.1193195 | 0.36898738 | 0.56144525 |
| Left cuneus volume | 0.12260126 | 0.32730845 | 0.52369353 |
| Left entorhinal volume | 0.08860371 | 0.46356422 | 0.64092876 |
| Left fusiform volume | -0.0224244 | 0.87747954 | 0.91845902 |
| Left inferiorparietal volume | -0.2584502 | 0.053583 | 0.16193975 |
| Left inferiortemporal volume | -0.0243394 | 0.86555115 | 0.91251904 |
| Left isthmuscingulate volume | 0.07718129 | 0.57546787 | 0.72466325 |
| Left lateraloccipital volume | 0.11045467 | 0.43897233 | 0.62187747 |
| Left lateralorbitofrontal volume | 0.14240248 | 0.39426419 | 0.58282532 |
| Left lingual volume | -0.0135983 | 0.91628046 | 0.93181306 |
| Left medialorbitofrontal volume | 0.04598731 | 0.75447228 | 0.83545367 |
| Left middletemporal volume | 0.13346923 | 0.37820717 | 0.57038663 |
| Left parahippocampal volume | 0.04194491 | 0.72640587 | 0.81984397 |
| Left paracentral volume | -0.303774 | 0.01710527 | 0.07588339 |
| Left parsopercularis volume | 0.25042022 | 0.0457226 | 0.13973648 |
| Left parsorbitalis volume | -0.0552049 | 0.67319608 | 0.80664904 |
| Left parstriangularis volume | 0.01280406 | 0.91810993 | 0.93181306 |
| Left pericalcarine volume | 0.04785445 | 0.69944816 | 0.81267537 |
| Left postcentral volume | -0.3905829 | 0.00645363 | 0.03703042 |
| Left posteriorcingulate volume | -0.13272 | 0.33238757 | 0.52871005 |
| Left precentral volume | -0.1795895 | 0.23558442 | 0.42157212 |
| Left precuneus volume | -0.0852796 | 0.57842328 | 0.72495606 |
| Left rostralanteriorcingulate volume | -0.1960637 | 0.15711841 | 0.32874006 |
| Left rostralmiddlefrontal volume | -0.1674314 | 0.26581054 | 0.44907122 |
| Left superiorfrontal volume | -0.2711752 | 0.08747646 | 0.23327055 |
| Left superiorparietal volume | -0.192482 | 0.16284577 | 0.33070839 |
| Left superiortemporal volume | -0.2248808 | 0.13938784 | 0.3067195 |
| Left supramarginal volume | -0.2519057 | 0.06371072 | 0.18435444 |
| Left frontalpole volume | 0.21618027 | 0.09073974 | 0.23432967 |
| Left temporalpole volume | -0.0324831 | 0.79175831 | 0.86838008 |
| Left transversetemporal volume | 0.04913883 | 0.69420196 | 0.81267537 |
| Left insula volume | -0.2130836 | 0.14806363 | 0.31656705 |
| Right bankssts volume | -0.1234653 | 0.35664809 | 0.55433304 |
| Right caudalanteriorcingulate volume | -0.2439339 | 0.04087318 | 0.1292733 |
| Right caudalmiddlefrontal volume | -0.1733245 | 0.18294388 | 0.35799089 |

| | | | |
|---|---|---|---|
| Right cuneus volume | 0.09016565 | 0.47974173 | 0.65903914 |
| Right entorhinal volume | -0.0687063 | 0.57316705 | 0.72466325 |
| Right fusiform volume | -0.0314647 | 0.83820512 | 0.89824071 |
| Right inferiorparietal volume | -0.2308679 | 0.10745503 | 0.26331322 |
| Right inferiortemporal volume | -0.0856609 | 0.55841133 | 0.72309315 |
| Right isthmuscingulate volume | 0.20070032 | 0.12842507 | 0.29109683 |
| Right lateraloccipital volume | 0.15860237 | 0.27472107 | 0.45563495 |
| Right lateralorbitofrontal volume | -0.2079322 | 0.19088198 | 0.36307621 |
| Right lingual volume | 0.0177373 | 0.89034489 | 0.92217693 |
| Right medialorbitofrontal volume | -0.0053102 | 0.97055454 | 0.97413592 |
| Right middletemporal volume | -0.0931496 | 0.55653908 | 0.72309315 |
| Right parahippocampal volume | 0.09787146 | 0.4270027 | 0.61128808 |
| Right paracentral volume | -0.2755759 | 0.03055538 | 0.1038883 |
| Right parsopercularis volume | 0.07170345 | 0.56588643 | 0.72309315 |
| Right parsorbitalis volume | -0.0625106 | 0.63193239 | 0.7742595 |
| Right parstriangularis volume | -0.0163905 | 0.89505408 | 0.92217693 |
| Right pericalcarine volume | 0.07423695 | 0.55510153 | 0.72309315 |
| Right postcentral volume | -0.3568461 | 0.01190529 | 0.05868709 |
| Right posteriorcingulate volume | -0.3136522 | 0.02069361 | 0.08528274 |
| Right precentral volume | -0.2894782 | 0.04503203 | 0.13918991 |
| Right precuneus volume | 0.03652493 | 0.8138129 | 0.88190084 |
| Right rostralanteriorcingulate volume | -0.0189055 | 0.8807713 | 0.91845902 |
| Right rostralmiddlefrontal volume | -0.053747 | 0.70810317 | 0.81267537 |
| Right superiorfrontal volume | -0.1997755 | 0.19273254 | 0.36405036 |
| Right superiorparietal volume | -0.047442 | 0.73084833 | 0.82144937 |
| Right superiortemporal volume | -0.306547 | 0.04442497 | 0.13889185 |
| Right supramarginal volume | -0.293744 | 0.02969438 | 0.10223887 |
| Right frontalpole volume | -0.0832468 | 0.52263841 | 0.70028398 |
| Right temporalpole volume | -0.0249834 | 0.83879831 | 0.89824071 |
| Right transversetemporal volume | -0.1767856 | 0.16274269 | 0.33070839 |
| Right insula volume | -0.1129763 | 0.4449877 | 0.62390029 |
| | | | |
| ***Subcortical analyses*** | | | |
| | | | |
| **Region** | *Beta* | *p (uncorr.)* | *p (FDR)* |
| Brain Stem | -0.104033 | 0.519081 | 0.59826285 |
| Left Thalamus | -0.1948715 | 0.2393556 | 0.35383002 |
| Left Caudate | -0.2137469 | 0.12959872 | 0.29650839 |
| Left Putamen | -0.0660591 | 0.62730713 | 0.68801427 |
| Left Pallidum | -0.1907139 | 0.19621879 | 0.29650839 |
| Left Hippocampus | -0.0774194 | 0.61312445 | 0.68801427 |
| Left Amygdala | -0.1231535 | 0.38010534 | 0.49706083 |
| Left Accumbens area | -0.0444386 | 0.76955018 | 0.81764707 |
| Left VentralDC | 0.21195046 | 0.17500029 | 0.29650839 |
| Right Thalamus | -0.2218255 | 0.16563137 | 0.29650839 |
